# Multi-element analysis of unfiltered samples in river water monitoring—digestion and single-run analyses of 67 elements

**DOI:** 10.1007/s00216-024-05270-4

**Published:** 2024-04-06

**Authors:** Nadine Belkouteb, Henning Schroeder, Jan G. Wiederhold, Thomas A. Ternes, Lars Duester

**Affiliations:** Division G – Qualitative Hydrology, Federal Institute of Hydrology, Am Mainzer Tor 1, 56068 Koblenz, Germany

**Keywords:** Acid digestion, Whole water samples, ICP-MS/MS, Multi-element, Temporal trends

## Abstract

Routine analysis of inorganic analytes in whole water samples from rivers (unfiltered river water) is rarely reported in scientific publications. However, this sample type is valuable and often used in long-term monitoring, regulation, and catchment element budgets, as it includes the dissolved, colloidal, and particulate fraction in one sample type. Preservation measures are not needed and solid–liquid partitioning can be disregarded, which simplifies automated sampling and storage procedures. In this study, we provide several digestion protocols for whole water samples from rivers and the subsequent multi-element analysis of 67 major, minor, and trace elements: Li, Be, B, Na, Mg, Al, Si, P, S, K, Ca, Sc, Ti, V, Cr, Mn, Fe, Co, Ni, Cu, Zn, Ga, Ge, As, Se, Br, Rb, Sr, Y, Zr, Nb, Mo, Ru, Ag, Cd, In, Sn, Sb, Te, Cs, Ba, La, Ce, Pr, Nd, Sm, Eu, Gd, Tb, Dy, Ho, Er, Tm, Yb, Lu, Hf, Ta, W, Ir, Pt, Au, Hg, Tl, Pb, Bi, Th, U. In the absence of whole water reference materials for inorganic analytes, we introduce simulated whole water samples by suspending sediment reference materials as quality control measures. The applicability for improved routine water quality monitoring was successfully tested on samples from different rivers revealing variations of the element fingerprints over time.

**Graphical abstract:**

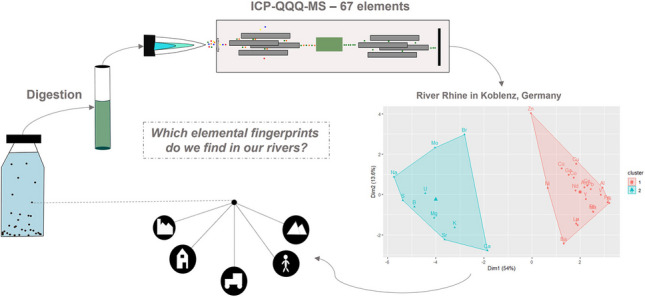

**Supplementary Information:**

The online version contains supplementary material available at 10.1007/s00216-024-05270-4.

## Introduction

“Whole water” samples (i.e., unfiltered) consist of several fractions including particulate, colloidal, and dissolved matter. Chemical compounds are therefore often analysed either in the dissolved (by convention < 0.45 µm or < 0.2 µm) or particulate/non-dissolved fraction (> 0.45 µm) [1]. Different size cut-offs are used to study nanoparticulate and colloidal matter in natural waters [2, 3]. For element analysis, the dissolved sample is commonly used as reference sample, for example according to the EU Water Framework Directive (WFD, Directive 2000/60/EC, [4]), while organic analyses should be conducted on the whole water sample [5–7]. The term “whole water” is here defined as the “synonym for the original water sample and shall mean the water sample when solid matter and the liquid phase have not been separated” according to a WFD guideline by the European Communities (2009) [8]. As an example, the International Commission for the Protection of the Rhine (ICPR) is continuously analysing the whole water fraction [9] in addition to the mandatory dissolved and particulate fraction according to the WFD. This is due to the fact that the longest lasting historical time series of monitoring data are available for this sample type (starting in 1978 decades before the convention to filtered samples was adopted in approximately 2008) and because it provides the opportunity to directly calculate annual element budgets for the river Rhine (https://iksr.bafg.de).

The transportation process of elements in rivers depends next to the discharge on the amount of transported suspended particulate matter (SPM) as well as on the element-specific speciation, fractionation, and hence, mobility [1, 10]. Therefore, to have a comprehensive overview on the element distribution in river water, it is possible to analyse (i) the SPM and the filtered water or (ii) the whole water and the filtered water. As stated by Harhash et al. (2023) for SPM sampling, the best approaches are the continuous flow centrifuge and the vacuum filtration [11]. However, both are not easy-to-handle sampling devices when it comes to field campaigns with several samples and low SPM loads. Moreover, the separation process of SPM from the water is accompanied by a small analyte loss since it is not possible to recover 100% of all SPM even after making use of the best-practice. For the dissolved fraction, water samples have to be filtered and acidified immediately after sampling, to avoid fractionation, changes during storage, and thereby analyte partitioning artefacts between SPM and water [12]. In this case, analysing the whole water sample, containing all fractions, is a unique opportunity, without timely preservation steps, that allows to draw conclusions on element transportation processes even at low SPM concentration. A challenge however is to take a representative whole water sample. One possibility is to collect integrated samples over a certain period of time. For the calculation of element budgets, the whole water sample cannot be replaced for the complete assessment of all river water components, especially at low SPM concentrations and in rural areas.

As an example from the recent literature, Dendievel et al. (2022) compiled data on the chemical composition of sediments and SPM originating from major European rivers in the time period 1945 to 2020 [13]. However, only data for Al, Cr, Fe, Ni, Cu, Zn, Cd, Hg, and Pb were provided and all other elements were not included in the data series. Therefore, the continuous analysis of a larger number of analytes with potential relevance for future impacts is of high interest to close knowledge gaps within catchment area budgets. Next to a fundamental understanding of regional and global element cycles, potential emerging pollutants are of interest. The list of “critical raw materials” by the European Commission is constantly updated. Consequently, analytes such as the so-called technology critical elements (TCE) are of rising concern and not much environmental monitoring data exists for most of them [14]. A fast overview on element “fingerprints” can be gained by applying methods that provide a complete picture of different analytes which is crucial when it comes to the detection of possible adverse changes in rivers. In this regard, multi-element analysis including a high number of analytes and requiring only one single analytical run is a valuable tool for monitoring approaches. An example was the fish kill event in the Oder River in summer 2022 [15, 16]. The multi-element analysis of filtered and whole water samples from the event provided valuable information and helped to narrow down potential causes [17].

From an analytical point of view, whole water samples are more challenging than the filtered counterparts (dissolved fraction, < 0.45 µm). For element analysis, inductively coupled plasma mass spectrometry (ICP-MS) is the analytical method of choice [18–20]. Samples in high-throughput routine processes are introduced as liquids into the ICP-MS and therefore acid digestion is required for whole water samples prior to analysis to ensure complete transfer of a homogeneous solution into the plasma. Current standardised methods for water sample digestions cover only a limited number of analytes of 26 to 31 elements (US EPA 200.2 (1994), US EPA 3015A (2007), or ISO 15587 (2002) [21–23]) while available analytical techniques provide the opportunity to significantly expand the set of analytes. Consequently, a digestion method targeting multi-element applications and suitable for ICP-MS analysis of natural water samples is of very high interest. In a previous study, we introduced a best-practice method for the multi-element analysis of the dissolved fraction of river water samples [17]. In this study, digestion protocols and their advantages and disadvantages in connection with the analysis of 67 elements in river water (Li, Be, B, Na, Mg, Al, Si, P, S, K, Ca, Sc, Ti, V, Cr, Mn, Fe, Co, Ni, Cu, Zn, Ga, Ge, As, Se, Br, Rb, Sr, Y, Zr, Nb, Mo, Ru, Ag, Cd, In, Sn, Sb, Te, Cs, Ba, La, Ce, Pr, Nd, Sm, Eu, Gd, Tb, Dy, Ho, Er, Tm, Yb, Lu, Hf, Ta, W, Ir, Pt, Au, Hg, Tl, Pb, Bi, Th, U) in a single run are presented and critically discussed. One closed-vessel and two open-vessel approaches were compared. Since no reference materials (RMs) for inorganic analytes in whole water samples are available so far, a fast approach is proposed to create simulated whole water samples by preparing a suspension with sediment RMs. Digestion parameters and other relevant factors addressed include the time and temperature of the digestion, the sample-to-reagent-ratio, the acid strength, the composition of the digestion reagent, and the amount of SPM in the sample. Challenges in optimising a whole water digestion protocol for inorganic analysis are firstly that there are no whole water RMs available; secondly, the sediment RMs do not cover the whole range of target analytes and more important; and thirdly, there is a limited number of sediment RMs which have both, aqua regia and total content referenced values. The results are compiled to present a best-practice method for whole water samples which is still compatible with the respective standard method (ISO 15587 (2002), [23]), but which enables the analysis of 38 additional elements. Finally, our goal was to test whether the optimised method was able to provide reliable data in “real life” river monitoring applications with varying hydrological conditions and SPM loads.

## Materials and methods

### Reagents, standard solutions, and materials

For ICP-MS analysis, the following surface water RMs were chosen as quality control standards in the method development to cover as many analytes as possible: SPS-SW1, SPS-SW2 (both Spectrapure Standards AS, Norway), NW-TM-15.3, NW-TM-24.4, NW-TM-35, NW-TMDA-51.5 (Environment and Climate Change Canada, RM Sales, Canada), and NIST SRM 1640a (National Institute of Standards and Technology, USA). For the limited number of elements which are not present in at least one of the RMs (Ge, Br, Zr, Nb, Ru, In, Te, Hf, Ta, W, Ir, Pt, Au, Hg), calibration check solutions were used as quality control standards according to Belkouteb et al. (2023) [17]. For the sediment RMs, please refer to paragraph 2.4.

HNO_3_ (65% w/w, EMSURE®, Merck GmbH, Germany) and HCl (37% w/w, EMSURE®, Merck GmbH, Germany) were further purified by sub-boiling distillation (DST-1000, Savillex, USA). Ultrapure water (≤ 0.055 μS/cm (corresponding to ≥ 18.2 MΩ·cm), Arium mini water purification system, Sartorius, Germany) was used for dilutions and cleaning processes.

Single element standards were purchased from Merck (Merck GmbH, Germany). ^103^Rh and ^185^Re were used as internal standards (ISTD) for ICP-MS analysis. For calibration of bromine, an ion chromatography standard was used (Merck GmbH, Germany).

Solutions were prepared in volumetric flasks (polypropylene (PP), 50 or 100 ml) and centrifuge PP vials (15 ml or 50 ml, VWR catalyst Laboratory Services, USA and SCP Science, Canada). All vessels were filled with 1.3% HNO_3_ for > 24 h. Prior to utilisation, they were rinsed three times with ultrapure water and dried under clean room conditions (laminar flow box, SPETEC GmbH, Germany).

### ICP-QQQ-MS analysis

For ICP-QQQ-MS analysis, an Agilent 8900 instrument (Agilent Technologies, Japan) with the three cell gases He, H_2_, and O_2_ was used. The term ICP-QQQ-MS is used here to describe a triple-quadrupole plasma mass spectrometer which uses two mass filters before and after a collision/reaction cell. Such an instrument can also be described in more general terms as an ICP-MS/MS. The sample introduction system consists of a Peltier-cooled quartz spray chamber, a MicroMist glass concentric nebulizer with 40-µm particle tolerance, a quartz torch with a 2.5-mm injector, a nickel-plated sampler cone, a nickel skimmer cone, x-lenses (Agilent Technologies, Japan), and an autosampler (ESI SC4 DX, ESI Elemental Service & Instruments GmbH, Germany). For tuning purposes, a custom-made Agilent solution containing 10 µg/l of Li, Co, Y, Ce, and Tl was used. The tuning was performed daily before each analysis run including the evaluation of the sensitivity of each analysis mode (He, H_2_, and O_2_), the relative standard deviation (< 3%), the oxide formation rate (^140^Ce^16^O^+^/^140^Ce^+^  < 2%), and the doubly charged ion ratio (^140^Ce^++^/^140^Ce^+^  < 3%).

For analysis of the samples, a multi-element method described earlier [17] was applied and slightly adapted according to the needs of the present study. The published method includes 68 elements: Li, Be, B, C, Na, Mg, Al, Si, P, S, Cl, K, Ca, Sc, Ti, V, Cr, Mn, Fe, Co, Ni, Cu, Zn, Ga, Ge, As, Se, Br, Rb, Sr, Y, Nb, Mo, Ru, Pd, Ag, Cd, In, Sn, Sb, Te, Cs, Ba, La, Ce, Pr, Nd, Sm, Eu, Gd, Tb, Dy, Ho, Er, Tm, Yb, Lu, Hf, Ta, W, Ir, Pt, Hg, Tl, Pb, Bi, Th, U. The decision process to include or exclude certain elements in the method is detailed in Belkouteb et al. (2023) [17]. In the present study, carbon and chlorine were excluded from the analyte list since chlorine is part of the digestion reagent and carbon is lost as CO_2_ during the digestion process. However, Au and Zr were added to the analysis spectrum. As discussed by Belkouteb et al. (2023) [17], Au and Zr need HCl stabilisation, and the method described therein is developed for filtered river water samples using only the matrix HNO_3_. Since for Pd, based on the results obtained in this study, there is an increased background in the real measurements, we assume an interference in the O_2_ mode, which was not yet verified. Therefore, we excluded Pd from the analyte list until further clarification. Therefore, the following 67 analytes were part of the analyte spectrum: Li, Be, B, Na, Mg, Al, Si, P, S, K, Ca, Sc, Ti, V, Cr, Mn, Fe, Co, Ni, Cu, Zn, Ga, Ge, As, Se, Br, Rb, Sr, Y, Zr, Nb, Mo, Ru, Ag, Cd, In, Sn, Sb, Te, Cs, Ba, La, Ce, Pr, Nd, Sm, Eu, Gd, Tb, Dy, Ho, Er, Tm, Yb, Lu, Hf, Ta, W, Ir, Pt, Au, Hg, Tl, Pb, Bi, Th, U. Moreover, the concentration ranges needed to be adapted for several elements to account for the much higher concentrations in the unfiltered river water (e.g., higher Al or Fe concentrations). Briefly, three calibration series were used for (i) HNO_3_ stabilised elements, (ii) HCl stabilised elements, and (iii) non-metals. All analyses were conducted with a 10-point calibration. Detailed compositions of stock and calibration solutions are provided in Table S1. All ICP-MS analyses were quality/performance checked using the three surface water reference materials SPS-SW-1, SPS-SW-2, and TMDA 51.5 as well as calibration check solutions (cf. Section "Reagents, standard solutions, and materials"). For the following elements, only calibration check solutions could be considered due to the lack of surface water CRMs: Ge, Br, Zr, Nb, Ru, In, Te, Hf, Ta, W, Ir, Pt, Au, and Hg. However, they were additionally validated by spike recoveries in Belkouteb et al. (2023). The total analysis time per sample including transfer time and washout is 4 min and 40 s.

### Digestion instrumentation

For closed-vessel digestion approaches, the microwave device MARS 6 (CEM, Germany) was used. For open digestion, an automated pipetting and heating system, the DEENA 3 (Seal Analytical, USA), and a beaker on a hot plate approach were applied. Both the MARS 6 and the DEENA digestions were conducted with polytetrafluoroethylene (PTFE) vessels while for the beaker approach, glass beakers were used on a hot plate. For cleaning purposes, the vessels were cleaned with ultrapure water and a cleaning brush, they were afterwards filled with 1:1 diluted 65% HNO_3_, and then a cleaning digestion run was undertaken. Finally, the vessels were rinsed three times with ultrapure water and left to dry under clean room conditions in a laminar flow box.

### Simulated whole water samples

Even though whole water samples are very common in river water monitoring approaches, no reference materials for inorganic analytes are available. To simulate whole water samples, eight sediment RMs values were used for quality control: CRM-MS-S (High-Purity Standards, USA), Metranal 1 and Metranal 18 (ANALYTIKA, spol. s r.o., Czech Republic), NWHR-1 and NWWQB-1 (Environment and Climate Change Canada), SdAR-M2 (International Association of Geoanalysts, UK), BCR 667 (European Commision – Joint Research Centre, Belgium), and JSd-3 (Geological Survey of Japan). Aqua regia extractable values were available for five RMs (Metranal 1, Metranal 18, NWHR-1, NWWQB-1, SdAR-M2), US EPA 3050B digestion values by using HNO_3_ and H_2_O_2_ as reagents in one RM (CRM-MS-S) as well as total digestion values for Metranal-1, BCR 667, JSd-3, and SdAR-M2. The sediment reference materials were chosen to cover as many analytes as possible, to address a variety in mineralogical compositions and concentration ranges of the elements as well as based on available information about total and aqua regia extractable values (Tables S2 and S3). No aqua regia extractable values were available for Si, S, Ge, Br, Ru, Pr, Nd, Tm, Ta, Ir, Pt and Au. For Si, S, Br, Pr, Nd, Tm, Ta, Pt, and Au, only referenced or indicative values following a total digestion were available (refer to Table S2). All sediment RMs were additionally analysed via energy-dispersive X-ray fluorescence (XRF) spectroscopy (Spectro XEPOS, SPECTRO Analytical Instruments GmbH, Germany) and the results, which were used as a supporting information for the ICP-MS analyses, are presented in Table S4. The focus was not the development or comparison of an XRF method. Simulated whole water samples were produced from the sediment RMs as quality controls within the method development. The sediment RMs were weighed into a 1-l sample bottle (e.g., 0.25 g). Afterwards, 1 l of ultrapure water was added and the suspension was shaken vigorously. A concentration of 0.25 g/l SPM is similar to a high discharge level at the river Rhine in Koblenz (Germany) and was used as reference sample for each digestion run for whole water samples.

All whole water samples were shaken vigorously prior to aliquotation to ensure that all particles were suspended and homogeneously distributed in the bottles. A 25-ml measuring glass pipette was used for aliquotation and 20 ml (minimum volume according to ISO 15587 (2002) [23]) of the water samples was used for all whole water digestions. All samples were available in 1-l bottles. Differences in particle sizes and mineralogical compositions can lead to different digestion efficiencies of some elements [24]. Thus, it is crucial that the aliquotation of whole water samples should be conducted as careful as possible. According to DIN-38402–30 (1998) [25], all water samples having a volume < 5 l can be shaken by hand and a representative aliquot can be taken. This was tested with five replicates (*n* = 5) of simulated whole water samples. Results are available in Section 4 of the SI.

### Digestion parameters

**Fig. 1:**
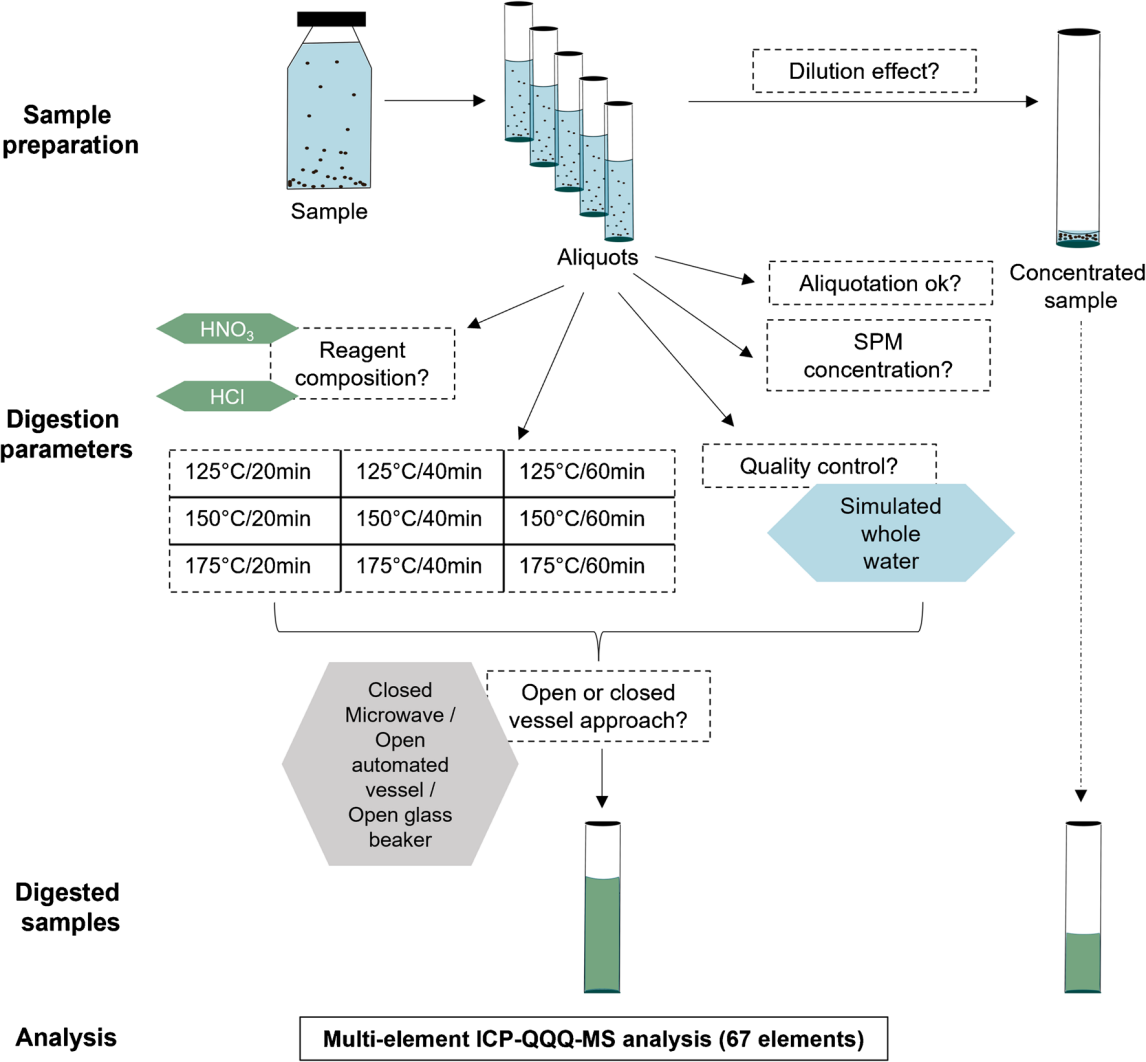
Overview on the tested parameters for the digestion of whole water samples by remaining comparable to ISO 15587 (2002) [23]. Boxes with dashed outline indicate specific aspects of the method development which were addressed in this study

To find an optimal approach for digestion, several parameters, namely the reagent composition, temperature, time, sample volume, reagent volume, and the concentration of the suspended material, were investigated. An overview of the conducted steps can be found in Fig. 1.

#### Digestion reagent

HNO_3_ is an oxidising agent and reacts strongly with organic matter. Aqua regia, a 1:3 mixture of HNO_3_ and HCl, is known to be a digestion agent with higher digestion efficiency due to the formation of nitrosyl chloride which is capable to digest more stable mineral phases such as oxides, sulphides, and sulphates [26, 27]. Refractory minerals such as zircon, chromite, rutile, or barite might however not be digested by this protocol [28]. Since one goal of this study was to only assess the long-term environmentally available fraction, to remain comparable to the ISO 15587 (2002) [23] and the long-term monitoring data series in Germany, hydrofluoric acid (HF) was not considered a possible digestion reagent component [29]. HF is required when the silicate fraction is addressed; however, the formation of poorly soluble or insoluble fluorides (e.g., with Al or Ca) is a common issue [29, 30]. Furthermore, HF is in our case not recommended for routine purposes when analysing hundreds of whole water samples due to the increased safety requirements, long digestion protocols and the necessity of adding boric acid after digestion to avoid insoluble fluorides which would lead to the exclusion of boron from the analyte spectrum. Therefore, different ratios and volumes of HNO_3_ to HCl were evaluated for digestion using the sediment RMs (CRM-MS-S, Metranal-18, Metranal-1, NWHR-1, NWWQB-1, BCR 667, JSd-3). At this stage of the experiments, the RM SdAR-M2 with the highest number of referenced aqua regia extractable values was not yet available. Another initial consideration tested was that a higher amount of HNO_3_ than HCl could be preferable due to the following reasons: (i) HNO_3_ is the oxidising agent. The more organic carbon in the sample, the higher the amount of oxidising agent needed. Especially, in our experience, whole water samples containing algae and bacteria in growth phases may exhibit large amounts of organic carbon. (ii) In routine analyses with ICP-MS, it is preferable to have less HCl included to reduce maintenance efforts.

#### Temperature and time

ISO 15587 (2002) defines a range of temperatures and reaction times in which it is assumed that similar digestion conditions are obtained [23]. To check this assumption and, if necessary, narrow the parameter ranges, different time and temperature programs were evaluated in this study to find an optimised digestion method which should be as short and efficient as possible. Firstly, at the beginning of the different digestion reagents and sample compositions tests, the temperature/time program 150 °C and 20 min, being in the allowed temperature/time-frame of ISO 15587 (2002) [23], was chosen as a starting point. Furthermore, for applying a wider range, the temperatures 125, 150 and 175 °C combined with the hold times 20, 40, and 60 min were included. Out of the 9 tested combinations, the temperature/time-programs 150 °C/20 min, 150 °C/40 min, 150 °C/60 min, and 175 °C/20 min corresponded to the temperature/time-frame proposed by the ISO 15587 [23]. Firstly, the 9 combinations, not all being in the frame of the ISO standard, were conducted for sediment digestions of CRM-MS-S, Metranal-1, NWWQB-1, and BCR 667. Secondly, the temperature/time-programs 150 °C/20 min, 150 °C/60 min, and 175 °C/20 min, being in the frame of the ISO standard, were chosen for further testing on simulated whole water samples of CRM-MS-S, Metranal-1, NWWQB-1, BCR 667 and SdAR-M2.

#### Sample and reagent volumes

The digestion efficiency is known to depend on the sample and reagent volumes. Since we are handling water samples, a dilution of the acid strength is an important aspect that was considered in the method development. Therefore, we tested the digestion efficiency for the RMs Metranal-1, NWHR-1, and NWWQB-1 by weighing 0.3 g of sediment into the digestion vessels and by suspending them in different volumes of ultrapure water (1, 2, 5, 10, 15 ml) prior to the acid digestion for which an aqua regia mixture was added: 6 ml HCl and 2 ml HNO_3_. Results are described in Section 5 of the SI.

#### Concentration of suspended particulate matter

Another parameter that has to be considered when digesting whole water samples is the SPM concentration. To see variabilities between different SPM concentrations, simulated whole water samples with the concentrations 0.1, 0.25 and 1 g/l were analysed. Results are described in Section 6 of the SI.

#### Concentrating the whole water sample

To check if the reduction of the water volume improves the overall digestion efficiency, the samples were concentrated with an additional device, the XpressVap (CEM, Germany), for the microwave MARS 6 made of PTFE. The device allows to concentrate suspensions by partial evaporation prior to or after a digestion run. In this study, it was used to concentrate the sample prior to digestion so that (i) the acids used for digestion were less diluted by the water from the sample, (ii) a better matrix matching to the standards used for ICP-MS by avoiding varying acid strength was reached, and (iii) the overall contribution of this factor to the measurement uncertainty could be tested. Potential disadvantages might be a loss of potentially volatile species (e.g., Hg) and an unsatisfying workload/benefit balance due to the higher effort in sample preparation. Results are described in Section 7 of the SI.

### Case study

To test the applicability and robustness of the optimised digestion protocol and to visualise trends with the method in the river Rhine, unfiltered river water samples from the sampling locations Weil (Upper Rhine, close to the border of Germany to Switzerland), Koblenz (Middle Rhine), and Wesel (Lower Rhine, close to the border of Germany to the Netherlands) from December 2018 were analysed. A scenario shifting from distinct low water flow conditions lasting from August to November 2018 (https://undine.bafg.de/rhein/extremereignisse/rhein_extremereignisse.html) to high water discharge was observed in December 2018. Additionally, samples from the river Moselle in Koblenz prior to its confluence with the river Rhine were analysed to extend the test to another river with different water composition. All investigated samples were daily integrated samples collected by automated water samplers which continuously pump small aliquots of river water into 2.5-l bottles over a 24-h period before moving to the next bottle position in a cooled sampling cupboard containing 36 bottles. These samplers are part of a monitoring network for environmental radioactivity in German rivers [31] and the collected samples are transported monthly to the German Federal Institute of Hydrology in Koblenz. Afterwards, the water samples were stored frozen at − 20 °C until analysis.

### Statistics and graphs

For data analysis and presentation, the R software with the packages “tidyverse”, “corrr”, “stats”, “factoextra”, “cluster”, and “gridExtra” was used (R version 4.2.1, 2022, The R Foundation for Statistical Computing). Recoveries were calculated based on the available reference values of the RMs. LOQs of ICP-QQQ-MS measurements were calculated based on a minimum of ten blank replicates of the respective digestion matrix solution with the following equation: LOQ = x + 10 × SD with x being the mean value and SD being the standard deviation of all blank replicates. Measurement results below LOQ or with a relative standard deviation (RSD), indicating the repeatability precision, higher than 10% were not considered.

## Results and discussion

The results are presented in the same order as in the “Materials and methods” section. After testing the parameters shown in Fig. 1, a final optimised method was identified. Briefly, 8 ml HNO_3_ (65%, sub-boiled) and 4 ml HCl (37%, sub-boiled) are added to a 20-ml water sample for microwave digestion at 175 °C and a hold time of 20 min. It allows for the analysis of 67 elements in whole water samples. In the following, selected results of the parameter optimisation are presented and discussed based on which our optimised digestion procedure was established. As some of our results regarding Sections 2.5.3 to 2.5.5 are only included in the SI, we provide here some key findings (see SI Sections 4 to 7 for further details): In this study, it was sufficient to shake the 1-l bottles by hand prior to aliquotation for 10 s and to take the sample from the centre of the bottle using a pipet with a 3-mm orifice. No reduction in the digestion efficiency was observed with larger water volumes. Concentrating the sample with XpressVap leads to a loss of volatile elements such as Se or Hg. Moreover, regarding the tested SPM concentrations 0.1, 0.25, and 1.0 g/l, no clear pattern was observable; however, the most reproducible results regarding the aqua regia extractable values were achieved with 0.25 g/l.

### Matrix correction prior to ICP-QQQ-MS analysis

To evaluate the multi-element capability of certain digestion approaches, firstly a reliable measurement procedure is needed. The ICP-QQQ-MS method used in this study was adapted from our previous publication focusing on filtered water samples [17]. It is very important to match the matrix of all standard solutions used during ICP-MS analysis (blanks, quality controls, calibration standards, internal standard) to minimise drifts of the internal standard. This is a challenge, since each digested sample has a different composition regarding the acid consumption due to the fact that several chemical reactions are taking place during the digestion. Matrix-matched solutions are an option, to avoid considerable differences between samples. The matrix matching was conducted with diluted digestion matrix solution (i.e., digestion reagent diluted 1:50 with ultrapure water) for the samples, the calibration solutions and the ISTD as well as by adding HNO_3_ (65%, sub-boiled) or/and HCl (37%, sub-boiled) to the dissolved reference materials. Two examples of different analytical runs, with and without matrix matching, are shown in Fig. S5 demonstrating the need for matrix matching to obtain comparable results. The analytical run without matrix matching showed an abrupt decline of the ISTD signal when the first sample was measured while the matrix-matched analytical run exhibited a stable ISTD recovery. An additional advantage next to matrix correction is that the addition of acids such as HCl to the samples after the digestion also helps for stabilisation purposes [32].

### Open vs. closed-vessel

**Fig. 2:**
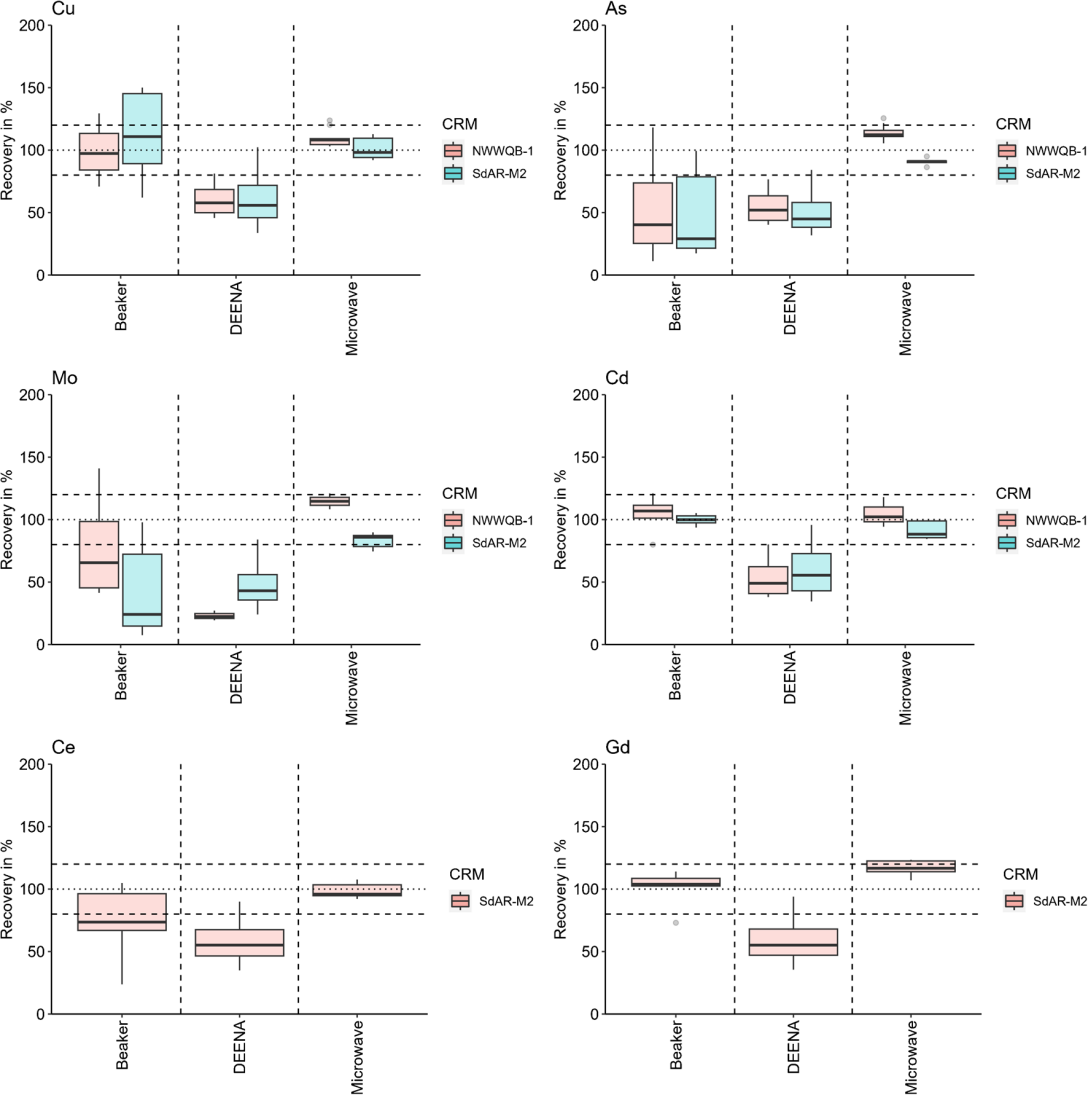
Recoveries of Cu, As, Mo, Cd, Ce, and Gd for selected RMs in three different digestion approaches for simulated whole water samples using aqua regia for the beaker approach (*n* = 6) and the optimised digestion reagent (2HNO_3_:1HCl) for the DEENA (*n* = 4) and microwave digestion (*n* = 10). Results for all other elements are shown in SI Section 8.2

The comparison of the three different digestion methods revealed that for Cu, As, Mo, and Ce, as examples for regularly (Cu and As) and rarely (Mo and Ce) analysed elements, only the microwave approach led to sufficient recoveries in the accepted range of 80 to 120% (Fig. 2). In contrast, the recoveries of the two open vessel digestions (beaker and DEENA) were below 80% and led to highly varying results as reflected by the lower and upper quartile of the boxplots in Fig. 2. Interestingly, Cd and Gd recoveries were similarly satisfactory in the beaker and the microwave approach. The results of the further elements are provided in Sect. 8.2 of the SI. It is often assumed that closed-vessel approaches yield superior digestion results as long as the samples are allowed to cool down with regard to possibly volatile elements such as As, Se, Cr, or Hg depending on whether they are stabilised or not [26, 33]. Advantages of open vessel approaches can be that a high number of samples can be handled in parallel and that the amount of reagent is reduced so that the acid strength is not disturbing further analyses [26]. Disadvantages of open vessel approaches are possible sample contamination or losses by boiling retardation, temperature gradients since some samples are already reduced to dryness while others are still boiling, and the loss of volatile species. For the microwave used in this study, the temperature of each single vessel was monitored during the whole digestion run. The temperature was constant during the hold time due to the automatic variation in the input power by the microwave program. The microwave approach was conducted with closed vessels and therefore boiling retardation was not an issue. For the investigated multi-element method, only the microwave approach provides sufficiently precise data based on the results of our tests and thus we recommend its use in future applications.

### Simulated whole water samples

As stated in Section "Simulated whole water samples", in absence of whole water RMs, these were simulated using sediment RMs suspended in ultrapure water. Recoveries in sediment and whole water digests of the RM SdAR-M2 exhibiting the highest number of aqua regia extractable values (53) including referenced and indicative values (refer to Table S2) are displayed in Table 1. Recoveries were mostly in the acceptable range of between 80 and 120% in relation to the aqua regia extractable values in the suspended RMs for 29 out of 53 elements, except for Na, P, K, Ca, Ti, Sc, Ga, Se, Rb, Sr, Zr, Nb, Ag, Sn, Te, Cs, Ba, Eu, Yb, Lu, Hf, W, and Tl, which exhibited recoveries outside this range in at least some of the tests. It has to be noted that recoveries which refer to the aqua regia extractable values can possibly exceed 100%. This is the case when the total content is higher than the aqua regia extractable content. The reference material data sheet of SdAR-M2 is referring to aqua regia extraction techniques that were undertaken at 85 to 160 °C (mostly at 90 to 110 °C) and using ICP-MS as well as inductively coupled plasma atomic emission spectrometry (ICP-AES) by the participating laboratories. Further differences between the participating laboratories are the mass portion used, the aqua regia composition, or the sample-to-acid ratio which are all components that were tested in this study. Therefore, recoveries > 100% relative to the aqua regia extractable concentration do not necessarily indicate a contamination but rather a potentially higher digestion efficiency which could be the case for Na, K, Ca, Ti, Sc, Ga, Rb, Sr, Nb, Sn, Te, Cs, Ba, Eu, Yb, Lu, Hf, W, and Tl, but not for Se, Zr, and Ag since their recoveries were below 80%. Exceptions in Table 1 for the sediment digestions are Zn, As, Cd, and Bi with recoveries slightly above 120% in relation to the referenced total contents. Reasons for Zn and As are that the referenced value for Zn (772 ± 19 mg/kg) and the indicative value of As (80 ± 3 mg/kg) for the total digestion of SdAR-M2 are slightly below the referenced aqua regia extractable values (792 ± 20 mg/kg Zn and 84 ± 3 mg/kg As) which is due to the uncertainties associated with the respective concentrations. For Zn, another possibility could be contamination through dust, as no clean room facilities were used in this study. However, the simulated whole water digestions for Zn, As, Cd, and Bi were below 120% regarding the total content. Moreover, the elements P, Se, Zr, and Ag exhibited recoveries of below 80% in the simulated whole water samples but not in the sediment digestions, which is possible due to the lower SPM concentration used in the simulated whole water samples in comparison to the sediment digests. It has to be noted that the results for P, Se, Zr, and Ag are all only based on comparison to indicative values. In addition, Zr might be hosted as zircon (ZrSiO_4_) in the SdAR-M2 sediment matrix which needs a stronger digestion procedure to be available in solution. For quality control in all further digestions of the optimised digestion protocol, simulated whole water samples with concentrations of 0.25 g/l of two RMs WQB-1 (*n* = 25) and SdAR-M2 (*n* = 53) were used to cover an element range which is as wide as possible with respect to available aqua regia extractable values.

**Table 1:** Recoveries of 60 elements (aqua regia (53) and total digestion (+ 7) referenced values) in % in aqua regia digestions at 150 °C and 20-min hold time of the RM SdAR-M2 digested either as solid sediment or as suspended “simulated whole water”. No recoveries of Li are shown for the whole water samples, since the concentration was below LOQ. Values in bold font indicate a recovery range of 80 to 120%. Values in italic font are higher than 120%. The numbers 1 and 2 indicate duplicate analyses of the same material/digestion approach

	Aqua regia digestion				Aqua regia digestion			
	Reference is aqua regia extractable value				Reference is total digestion value (as an additional information)			
	Solid SdAR-M2 1	Solid SdAR-M2 2	Water suspended SdAR-M2 1	Water suspended SdAR-M2 2	Solid SdAR-M2 1	Solid SdAR-M2 2	Water suspended SdAR-M2 1	Water suspended SdAR-M2 2
**Li**	**95**	**98**	*/*	*/*	68	70	*/*	*/*
**Be**	**109**	**114**	**102**	**103**	78	**81**	73	74
**B**	**103**	**110**	**82**	71	*/*	*/*	*/*	*/*
**Na**	*133*	*138*	*668*	*606*	3	4	17	16
**Mg**	*/*	*/*	*/*	*/*	79	**82**	71	72
**Al**	*/*	*/*	*/*	*/*	15	16	33	31
**Si**	*/*	*/*	*/*	*/*	0.3	0.3	14	14
**P**	**87**	**89**	67	69	76	78	59	60
**K**	*154*	*168*	*461*	*435*	9	10	28	26
**Ca**	**102**	**105**	*154*	*126*	50	51	75	61
**Sc**	*142*	*152*	*135*	*139*	66	71	63	65
**Ti**	*219*	*227*	*150*	*146*	47	49	32	31
**V**	*144*	*150*	**117**	**117**	**85**	**88**	69	69
**Cr**	*128*	*131*	**105**	**102**	20	20	16	16
**Mn**	**115**	**117**	**100**	**101**	**102**	**103**	**88**	**89**
**Fe**	**116**	**118**	**99**	**99**	**96**	**98**	**82**	**83**
**Co**	**103**	**104**	**94**	**96**	**106**	**108**	**97**	**99**
**Ni**	**116**	**117**	**105**	**107**	**112**	**113**	**101**	**103**
**Cu**	**107**	**110**	**112**	**111**	**110**	**113**	**115**	**113**
**Zn**	*129*	*130*	**109**	**107**	*132*	*133*	**112**	**110**
**Ga**	*197*	*206*	*233*	*238*	36	38	43	43
**As**	*123*	**119**	**92**	**97**	*129*	*125*	**96**	**102**
**Se**	*150*	*159*	76	58	*/*	*/*	*/*	*/*
**Rb**	*163*	*177*	*336*	*328*	17	19	36	35
**Sr**	*133*	*138*	*281*	*264*	17	18	37	34
**Y**	*136*	*142*	**113**	**115**	64	67	53	54
**Zr**	*195*	*227*	71	70	4	4	1	1
**Nb**	*389*	*407*	*301*	*300*	53	56	41	41
**Mo**	**104**	**108**	**89**	**87**	**106**	**111**	**91**	**89**
**Ag**	*132*	*133*	79	75	*/*	*/*	*/*	*/*
**Cd**	*127*	*129*	**113**	**111**	*127*	*129*	**113**	**112**
**In**	**108**	**110**	**98**	**99**	*/*	*/*	*/*	*/*
**Sn**	*129*	*129*	*135*	*131*	66	66	69	67
**Sb**	121	*127*	**114**	**114**	**108**	**113**	**101**	**102**
**Te**	*191*	*191*	*176*	*168*	*/*	*/*	*/*	*/*
**Cs**	*148*	*156*	*159*	*154*	65	69	70	68
**Ba**	*126*	*132*	*303*	*289*	14	15	33	32
**La**	*122*	*124*	**96**	**102**	**107**	**109**	**85**	**90**
**Ce**	121	121	**96**	**101**	**109**	**109**	**86**	**91**
**Pr**	*/*	*/*	*/*	*/*	**106**	**107**	**85**	**90**
**Nd**	*/*	*/*	*/*	*/*	**108**	**109**	**83**	**89**
**Sm**	*127*	*127*	**105**	**113**	**102**	**103**	**85**	**91**
**Eu**	*122*	*124*	*123*	*127*	49	50	50	51
**Gd**	121	121	**100**	**107**	**88**	**89**	73	78
**Tb**	*125*	*127*	**108**	**111**	79	**80**	68	70
**Dy**	*129*	*132*	**111**	**116**	77	78	66	69
**Ho**	*130*	*135*	**115**	**118**	67	69	59	60
**Er**	*129*	*134*	**116**	**117**	66	68	59	60
**Tm**	*/*	*/*	*/*	*/*	61	64	54	54
**Yb**	*141*	*149*	*126*	*128*	63	66	56	57
**Lu**	*149*	*156*	*134*	*134*	58	60	52	52
**Hf**	*236*	*269*	*172*	*168*	5	6	4	3
**Ta**	*/*	*/*	*/*	*/*	1	1	17	10
**W**	*254*	*269*	*252*	*216*	**81**	**86**	**80**	69
**Hg**	**118**	**120**	**96**	**98**	**118**	**120**	**96**	**98**
**Tl**	*129*	*135*	*133*	*129*	**87**	**90**	**89**	**86**
**Pb**	**120**	*123*	**111**	**112**	**119**	*122*	**110**	**111**
**Bi**	*137*	*128*	121	**115**	*135*	*126*	**118**	**113**
**Th**	*122*	*123*	**98**	**100**	**97**	**98**	78	79
**U**	*130*	*130*	**98**	**111**	75	75	57	64

### Digestion reagent

**Fig. 3:**
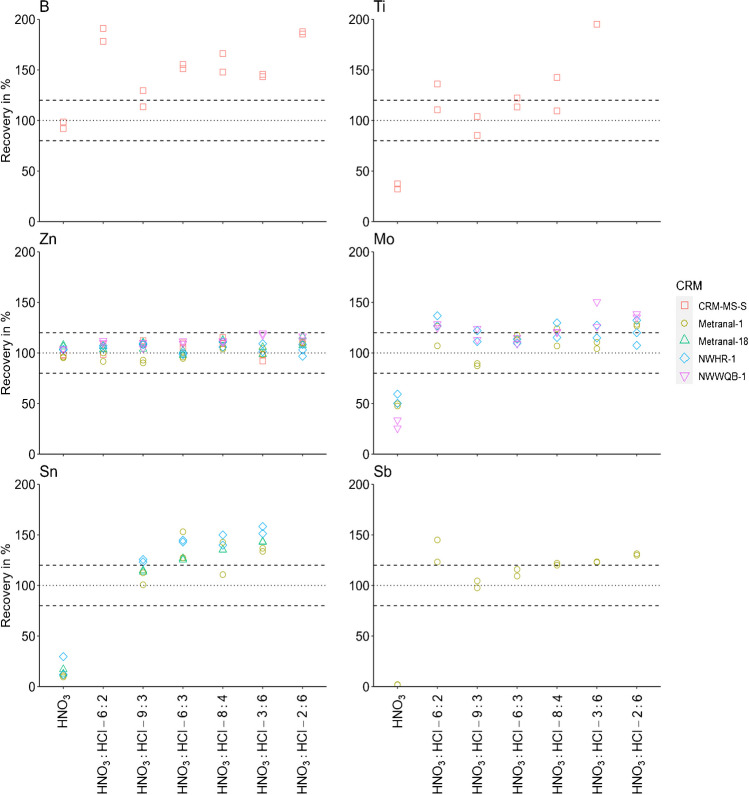
Recoveries of the elements B, Ti, Zn, Mo, Sn, and Sb in sediment digests (0.3 g, duplicates) with different acid ratios (e.g., HNO_3_:HCl 2:6 means 2 ml HNO_3_ and 6 ml HCl) at 150 °C and 20 min. The dotted line corresponds to the referenced aqua regia extractable element contents (100%), which may be lower than the total element content in the RM. Data points which are not shown are < LOQ or the RSD is > 10%. Ti recovery in the digestion HNO_3_:HCl 2:6 was > 200% and is therefore also not depicted

Zr, Ru, Ag, Sn, Sb, Hf, Ir, Pt, Au, and Hg are stabilised by HCl within the matrix, and hence a stabilisation with only HNO_3_ will not yield sufficient recoveries for these elements [17]. This has to be considered also in digestion: One element can be, for instance, sufficiently digested by using only HNO_3_ depending on the mineralogical composition but it needs to be stabilised later in solution. ISO 15587 (2002) describes that, for Sn and Sb, aqua regia needs to be applied for reliable results and for Al, Ba, Be, Cr, Fe, Mg, and V lower recoveries have to be expected using HNO_3_ only [23]. Moreover, it is stated that aqua regia is not sufficient to digest SiO_2_, TiO_2_, and Al_2_O_3_ [23]. Considering the results obtained in this study (selected results for B, Ti, Zn, Mo, Sn, and Sb in Fig. 3 and all further results in SI Sect. 8.3), we conclude that for analytes covered by regulations for river water monitoring within the EU (including German regulations) such as for example Zn but also Fe, Ni, and Cu, good recoveries within the accepted range of 80 to 120% with most of the tested HNO_3_ and HCl ratios were achieved. Consequently, there is no dependency on the HCl amount or on a specific HNO_3_ and HCl ratio. Moreover, it is once again emphasised that Sn and Sb require the presence of HCl for a successful digestion and stabilisation prior to the analysis. Interestingly, Ti and Mo also showed a HCl dependency (Fig. 3). It is evident that HCl is needed for digestion since both digestion runs without HCl showed recoveries below 80% for Ti and Mo, most likely because of the mineral phase composition of the RMs. For B, recoveries > 120% were obtained by adding HCl to the digestion reagent which could be either a possible contamination or a higher digestion efficiency if the total content is below < 120% as explained in Section "Simulated whole water samples". Unfortunately, no total content values are available for CRM-MS-S and no data could be obtained with XRF for B. Therefore, according to the available data for CRM-MS-S, only the HNO_3_ digestion led to recoveries for B in the acceptable range of 80 and 120%. For the RM SdAR-M2 used only at a later stage of the experiments due to its late availability, recoveries of B relative to the aqua regia extractable content were between 80 and 120% also by using aqua regia (refer to Table 1). This indicates that the digestion efficiency and the used reagent is also dependent on the mineralogical compositions of the different RMs. However, unfortunately, no information could be obtained about the mineralogical composition of each RM. All further considered analytes Li, B, Na, Mg, Al, K, Ca, V, Cr, Mn, Fe, Co, Ni, Cu, Zn, As, Se, Sr, Cd, Ba, Hg, Pb, Bi, and U, which were measured > LOQ and with a RSD < 10% in the RMs CRM-MS-S, Metranal-18, Metranal-1, NWHR-1, and NWWQB-1, did not show a clear pattern regarding the different HNO_3_ and HCl ratios. Moreover, Metranal-1, BCR 667, and JSd-3, exhibiting referenced total values, cover more elements (39, Table S10). A higher digestion efficiency was observed for Ga by using HCl in the digestion reagent for JSd-3 (HNO_3_, 34–35% and HNO_3_:HCl-8:4, 58–61%). All further elements reveal recoveries mostly < 120% except for Se (Metranal-1, JSd-3), Ag (Metranal-1, JSd-3), Pr (JSd-3), and Hg (Metranal-1, JSd-3). However, Se and Pr also exhibit referenced total values in BCR 667 with a recovery below 80%. Moreover, the high recovery for Ag is only shown in the first two digestion reagents HNO_3_ and HNO_3_:HCl-6:2. For Hg, there is no clear pattern visible since 7 out of 24 recoveries are randomly slightly above 120%. For the optimised digestion protocol, a digestion reagent using 8 ml HNO_3_ and 4 ml HCl was applied to have a sufficiently high HCl content and a higher HNO_3_ than HCl concentration as stated in Section "Digestion reagent".

### Time and temperature

**Fig. 4:**
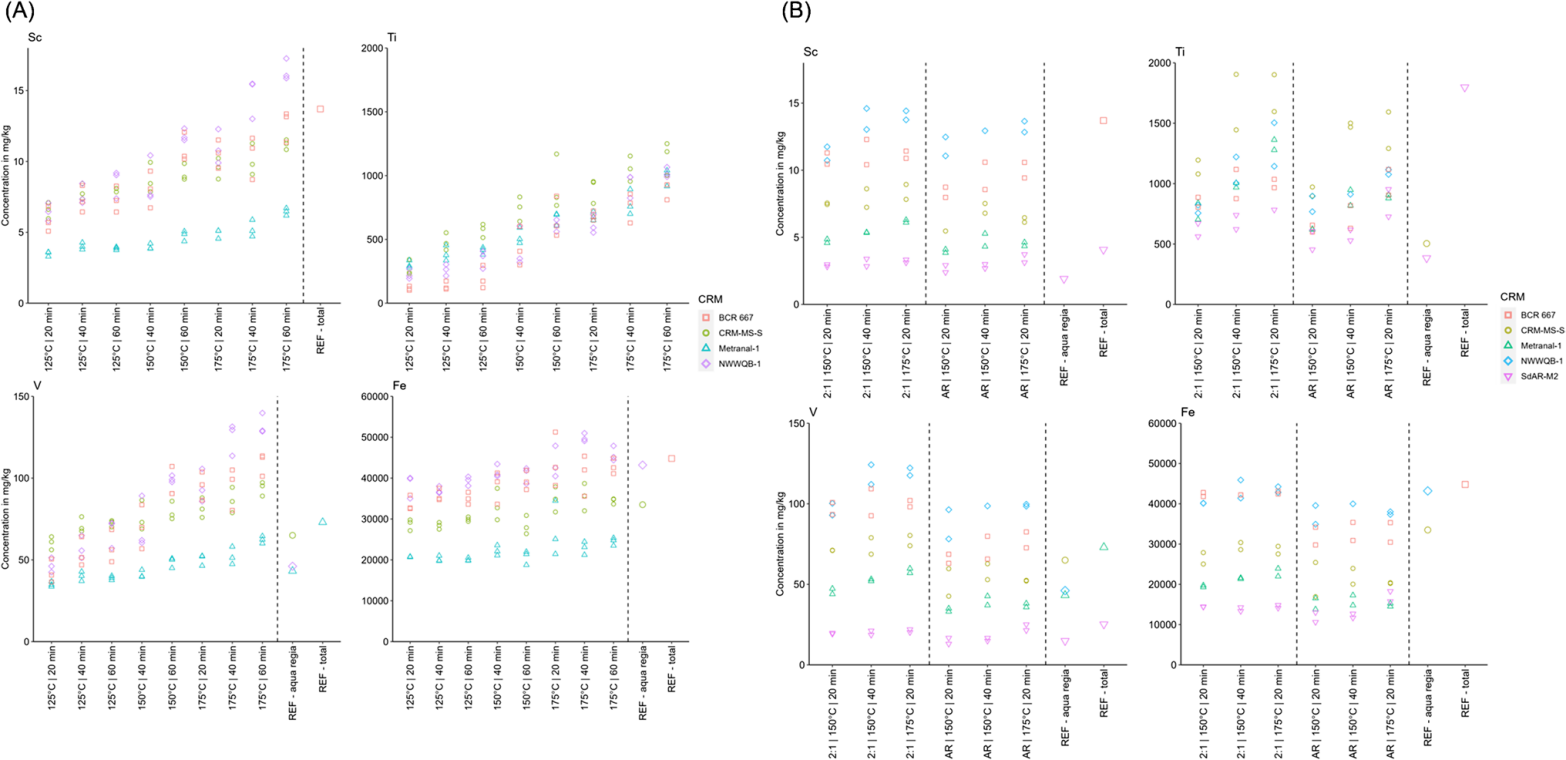
Exemplary results for Sc, Ti, V, and Fe in **A** sediment digests with a 2HNO_3_:1HCl mixture and in **B** simulated whole water samples at different temperature/time programs with a 2HNO_3_:1HCl mixture (shown as 2:1) and an aqua regia mixture (shown as AR). Three (**A**) or two (**B**) replicates were measured and they are all depicted. All digestions were conducted in the microwave. One data point in **A** for Fe at 175 °C | 20 min (51,300 mg/kg) is above the referenced total value (44,800 mg/kg) which is clearly an outlier since the other two concentration points are below 44,800 mg/kg. However, all values are shown for completeness. All further element results can be found in SI Section 8.4

Some of the elements measured showed a clear influence of the digestion duration and the temperature such as Ti (see Fig. 4). Here, instead of recoveries, the concentrations obtained after digestion are shown. In Section "Simulated whole water samples", it is already explained that concentrations higher than the aqua regia extractable content but below the total content could be achieved during the experiments which are not a contamination but rather a higher digestion efficiency as long as the concentration is below the total content. The highest concentrations of Ti in the four sediment RMs BCR 667, CRM-MS-S, Metranal-1, and NWWQB-1 were found at a microwave program with 175 °C and a hold time of 60 min. However, this program is outside of the application window of ISO 15587 (2002) [23]. Therefore, digestions of simulated whole water samples were conducted on three temperature programs which are included in the parameter window of the ISO 15587 (2002) (Part B of Fig. 4) to remain comparable to the standard [23]. For SdAR-M2, for instance, the total reference value of Ti is 1800 (± 18) mg/kg and the aqua regia reference value is 384 (± 30) mg/kg. Our measured value with XRF was 1620 (± 97) mg/kg. As depicted in Fig. 4, both the aqua regia and the 2HNO_3_:1HCl digestion showed higher concentrations than the aqua regia reference value of 384 (± 30.0) mg/kg but below the total reference value and the measured XRF value. In addition to Ti, V and Sc also showed a similar pattern (Fig. 4) of an increasing digestion efficiency with increasing time and temperature. According to ISO 15587 (2002), the same digestion results are to be expected for V if the digestion is conducted under conditions within the given parameter window [23]. However, the results show that the digestion efficiency for V can vary even by digesting within the given parameter window. Ti and Sc are not included in ISO 15587 (2002) [23]. Further results of all elements are found in the SI Section 8.4. To have a short and efficient digestion procedure which is still comparable to standardised ISO 15587 (2002) digestions, the temperature/time-program of 175 °C and 20 min was used in the optimised digestion protocol.

### Optimised digestion procedure

**Fig. 5:**
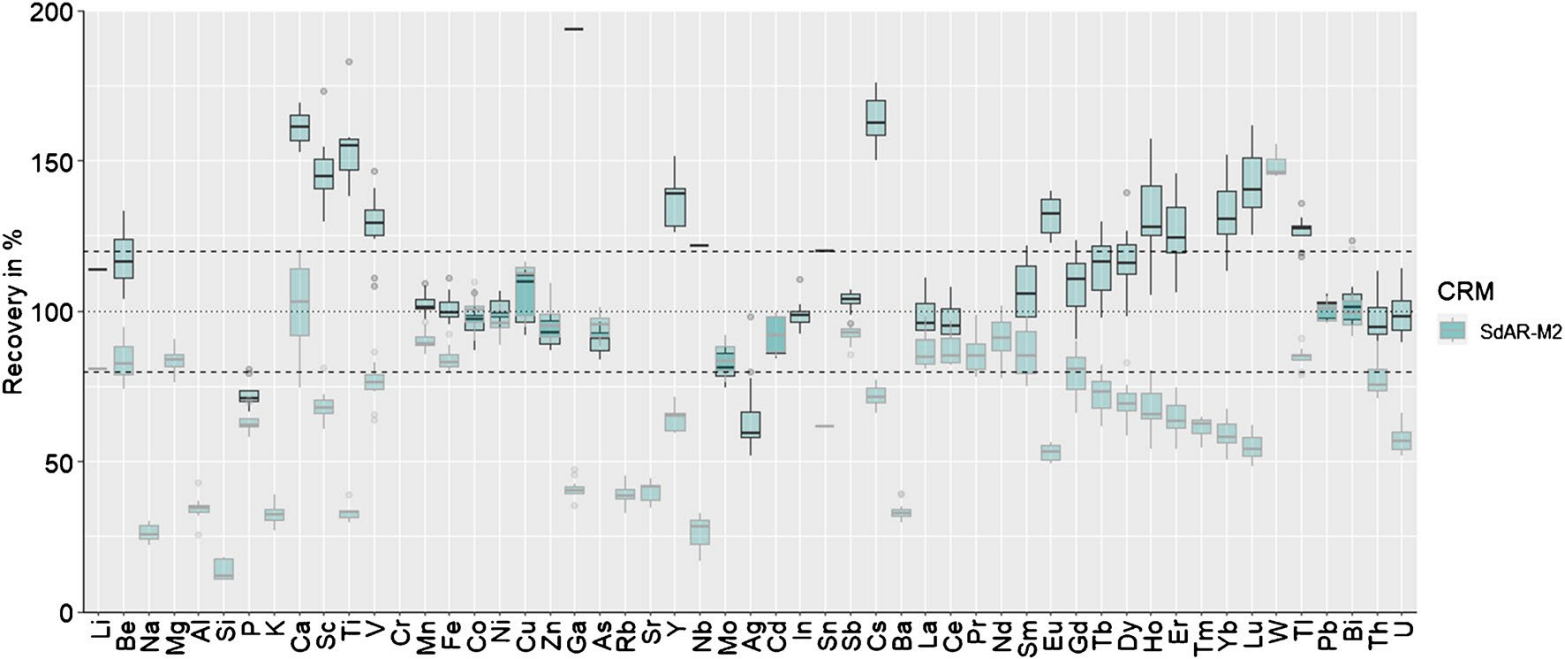
Recoveries of all 53 elements which are available as referenced or indicative values in the RM SdAR-M2 using the optimised digestion protocol (*n* = 15). Boxplots with a black frame refer to the referenced aqua regia extractable values while boxplots with a grey frame refer to the referenced total digestion values. Please note that recoveries > 100% relative to the aqua regia extractable values may indicate a higher digestion efficiency, as long as the values are still below 100% of the total element mass fractions. The following elements exhibited only indicative values for SdAR-M2: Na, P, K, Ti, V, Cr, Fe, Ga, Zr, Nb, Ag, Sb, Eu, Gd, Tb, Dy, Ho, Er, Th

The optimised digestion approach after examining all above-mentioned parameters which was used for the samples of Section "Case study" was as follows: 20 ml of the whole water sample was digested in a PTFE digestion vessel (55 ml) with 8 ml HNO_3_ (65%, sub-boiled) and 4 ml HCl (37%, sub-boiled). The microwave program consists of a ramp time of 20 min to 175 °C and a hold time of 20 min. The microwave power (max. 1800 W) is adjusted per number of vessels and it is optimised by the device during the microwave run as soon as the target temperature is reached. After digestion, the vessels were cooled down over night and then quantitively transferred to 50-ml volumetric flasks with diluted digestion matrix solution (2HNO_3_:1HCl diluted 1:50 with ultrapure water). For ICP-MS analysis, the transferred samples were again diluted in a 1:10 ratio with the diluted matrix solution. All standards used for ICP-MS analysis were matrix matched with the used diluted digestion matrix solution. Three blank replicates (ultrapure water) and three quality control replicates of two different simulated whole water samples (NWWQB-1 and SdAR-M2) were digested in each microwave run. Blank concentrations and recoveries of all microwave runs for the samples of Section 2.6 are included in Section 9 of the SI. Moreover, recoveries of the simulated whole water samples of SdAR-M2 using the optimised digestion method are depicted in Fig. 5. The gap between aqua regia and total referenced values is clearly visible. However, except for W, all elements did not exceed 120% of the total referenced values. The optimised digestion protocol is not targeting and does not aim to target refractory minerals as already stated in Section "Digestion reagent". Due to the already stated differences in the referenced values of the reference material data sheet of SdAR-M2 in Section "Simulated whole water samples", it has to be kept in mind that different digestion procedures were used for obtaining element mass fraction results in the reference materials. This leads to variations as seen in this study by applying even slightly different parameters. Moreover, indicative values are known to have higher variabilities and therefore differences are occurring more often.

### Case study

**Fig. 6:**
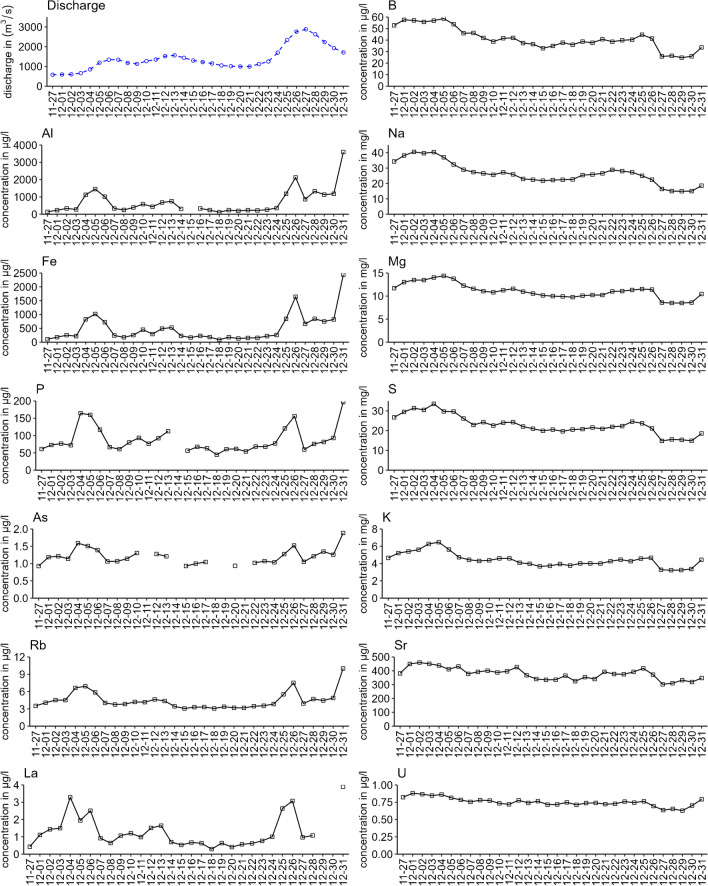
Concentrations of Al, Fe, P, As, Rb, La (first column) and B, Na, Mg, S, K, Sr, U (second column) in 24-h integrated whole water samples digested with the optimised method in comparison to the discharge in m^3^/s (blue plot) from 27 November to 31 December 2018 at the River Rhine in Koblenz. The right column shows elements with a dilution effect with rising discharge while the concentrations in the left column are co-rising. Results for further elements are found in SI Section 9. All concentrations < LOQ and with RSD > 10% are not depicted. Only elements with data points *n* > 15 are depicted to be able to see trends

For testing the applicability of the optimised digestion protocol, real-life river water samples showing a sudden change from low to high water discharge from the German part of the river Rhine in Weil (Upper Rhine), Koblenz (Middle Rhine), and Wesel (Lower Rhine) as well as from the river Moselle in Koblenz as a tributary of the river Rhine were analysed. There was a rapid increase in the water level by around 4 m resulting in a discharge increase from 581 m^3^/s (Nov 27) to 2880 m^3^/s (Dec 27) for the station Koblenz/Rhine in 1 month (Fig. 6).

**Fig. 7:**
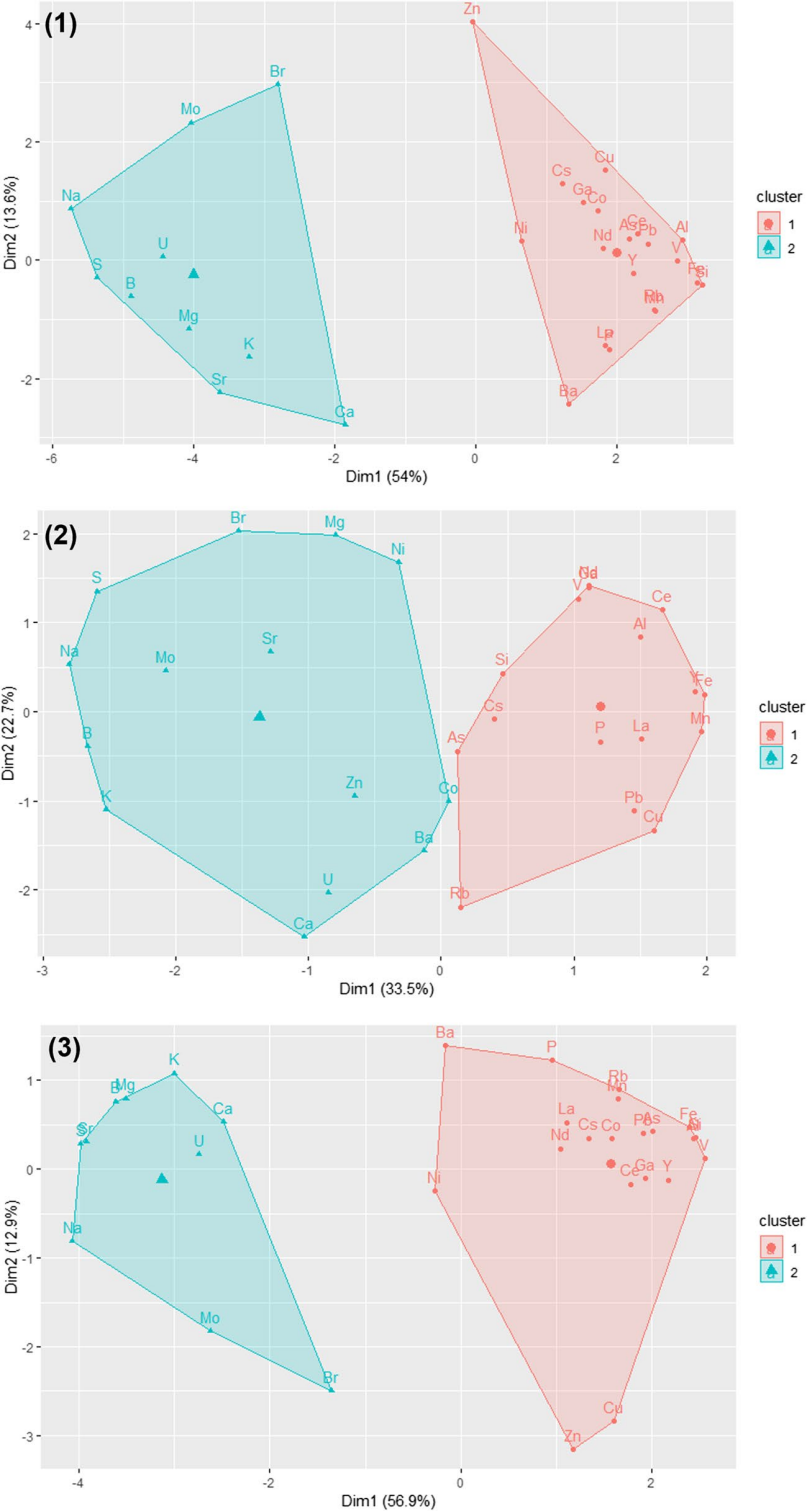
K-means clustering for the dataset of the station Koblenz/Rhine. The clustering was performed by using k-means (*k* = 2, elbow method) and by standardising the dataset before. Plot (1) reflects the complete dataset, while (2) and (3) are showing two different time stamps: (2) Dec 15 to Dec 21 (almost constant discharge level) and (3) Dec 22 to Dec 31 (peak of discharge)

The measured concentrations of the analysed 67 elements follow one of two distinctly different patterns over time. A clear pattern is visible between the right and left column of all graphs. While elements such as B, Na, Mg, S, K, Sr, and U (group A) show decreasing concentrations with higher discharge, the concentrations of elements such as Al, Fe, P, As, Rb, and La (group B) are co-rising with a higher discharge. A similar behaviour was also found for other elements (not included in Fig. 6 but in Figs. S6 and S7) such as Li, K, Sr, Mo, Br (similar to group A) and for V, Ga, Rb, La, Ce, Pb (similar to group B). A higher discharge leads to higher particle loads which leads to increased concentrations of Fe but also Al. P and As concentrations rise together with Al and Fe since they are strongly associated with their (oxyhydr)oxide mineral particles. Knapp et al. (2020) also showed a 2-year co-rising behaviour, for the dissolved fraction, for Fe and Mn due to their presence as oxides and their affinity to organic material [34]. Elements which show a dilution effect, e.g., B, Na, Mg, K, are mostly found in the dissolved fraction. Consequently, the higher the water volume, the lower the total concentration of dissolved elements. More results are available in Sect. 9 of the SI for the stations Koblenz/Rhine, Koblenz/Moselle, Wesel/Rhine, and Weil/Rhine. An interesting example is Rb. At the station Koblenz/Rhine and Koblenz/Moselle, a co-rising behaviour is observed while in Wesel/Rhine a dilution effect is visible. The region Koblenz/Moselle is influenced by high inclination wine agriculture while Wesel is located downstream of the densely populated and industrialised Ruhr basin. As a potential explanation, Rb is therefore predominantly found in particles dislocated from vineyards in the Moselle while in the Wesel/Rhine region it is mainly found in dissolved form from industry and household wastewater. The whole water sample is in this case a valuable sample matrix to assess the sum of dissolved elements and particle-bound elements in one analysis to observe long-term trends and elemental fingerprints in routine analysis. Cluster analysis of the Koblenz/Rhine samples revealed that discharge is one dominant factor for clustering element behaviour (Fig. 7). The dataset was divided into two sections to make differences in discharge levels visible. Clustering the complete dataset reveals the same division as seen in the plots of Fig. 6: elements with dilution (cluster 1) and with co-rising behaviour (cluster 2). Sections "Materials and methods" and "Results and discussion" of Fig. 7 are showing clusters at different discharge ranges of between 995 to 1300 m^3^/s and 1120 to 2880 m^3^/s leading to the observation that at lower discharge levels the clustering is not as clearly defined as for the discharge peak of Section "Results and discussion". The abrupt discharge variation in Section "Results and discussion" is causing the distinct element behaviour as described above. Therefore, having a larger long-term dataset with different discharge scenarios for a high number of elements can be valuable to detect anomalies in a certain discharge range.

## Conclusions

Digestion of whole water samples and subsequent multi-element analysis is a research topic which is not extensively studied and described in literature. Our study was conducted based on aqua regia digestions of whole water samples to stay comparable with existing long-term data series of German rivers while it aimed to develop a digestion approach which is as effective as possible for the analysis of 67 elements. Despite the non-existence of whole water reference materials, we show that the use of simulated whole water samples by suspending sediment RMs in ultrapure water is a satisfactory and rapid option for quality control. By analysing this sample type, a valuable overview on the water status can be gained since all water fractions are combined in one sample and the multi-element analysis in one single analytical run provides elemental fingerprints of the respective waterbody. This is crucial when a response on existing environmental problems and emerging issues in the future is needed. We therefore deliver a method that we hope ICP-QQQ-MS users in river water monitoring will adapt to extend existing parameter sets based on the recent state of the art for the benefit of their long-term data bases. Further conclusions on the riverine transport dynamics of different elements could be drawn when whole water samples and filtered water samples are analysed in parallel. This was not within the scope of the presented case study, but the new availability of validated methods for both filtered river water [17] and whole water samples of rivers (this study) will allow to perform such parallel analyses in future studies on multi-element fingerprint dynamics in river systems.

### Supplementary Information

Below is the link to the electronic supplementary material.

Supplementary file1 (PDF 5591 KB)

## References

[1] Gaillardet J, Viers J, Dupré B. Trace elements in river waters. In: Treatise on geochemistry (Second edition). Chapter 7.7, 2014;pages 195–235, Elsevier. 10.1016/B978-0-08-095975-7.00507-6.

[2] Buffle J, Perret D, Newman M. The use of filtration and ultrafiltration for size fractionation of aquatic particles, colloids, and macromolecules. (First edition). Chapter 5, 1992;pages 171–230. In: Environmental particles. CRC Press, Boca Raton. 10.1201/9780429286223.

[3] Fabricius A-L, Duester L, Meermann B, Ternes TA (2014). ICP-MS-based characterization of inorganic nanoparticles—sample preparation and off-line fractionation strategies. Anal Bioanal Chem.

[4] European Communities. Directive 2000/60/EC of the European Parliament and of the Council of 23 October 2000 establishing a framework for community action in the field of water policy. Official Journal L. 2000;327. https://eur-lex.europa.eu/eli/dir/2000/60/oj. Accessed 03/2024.

[5] Elordui-Zapatarietxe S, Fettig I, Philipp R, Gantois F, Lalère B, Swart C, Petrov P, Goenaga-Infante H, Vanermen G, Boom G, Emteborg H (2015). Novel concepts for preparation of reference materials as whole water samples for priority substances at nanogram-per-liter level using model suspended particulate matter and humic acids. Anal Bioanal Chem.

[6] Richter J, Elordui-Zapatarietxe S, Emteborg H, Fettig I, Cabillic J, Alasonati E, Gantois F, Swart C, Gokcen T, Tunc M, Binici B, Rodriguez-Cea A, Zuliani T, Gago AG, Pröfrock D, Nousiainen M, Sawal G, Buzoianu M, Philipp R (2016). An interlaboratory comparison on whole water samples. Accreditation Qual Assur.

[7] Dosis I, Ricci M, Emteborg H, Emons H (2021). A journey towards whole water certified reference materials for organic substances: measuring polycyclic aromatic hydrocarbons as required by the European Union Water Framework Directive. Anal Bioanal Chem.

[8] European Commission. Directorate-General for Environment, Guidance on surface water chemical monitoring under the water framework directive. Guidance document No 19, Publications Office, 2009. https://data.europa.eu/doi/10.2779/72701. Accessed 03/2024.

[9] ICPR (2020) Rheinmessprogramm Chemie 2021–2026 - International abgestimmtes Messprogramm gemäß Rheinübereinkommen und Überblicksüberwachung gemäß Wasserrahmenrichtlinie. https://www.iksr.org/fileadmin/user_upload/DKDM/Dokumente/Fachberichte/DE/rp_De_0265_d.pdf.

[10] Templeton DM, Ariese F, Cornelis R, Danielsson L-G, Muntau H, van Leeuwen HP, Lobinski R (2000). Guidelines for terms related to chemical speciation and fractionation of elements. Definitions, structural aspects, and methodological approaches (IUPAC Recommendations 2000). Pure Appl Chem.

[11] Harhash M, Schroeder H, Zavarsky A, Kamp J, Linkhorst A, Lauschke T, Dierkes G, Ternes TA, Duester L (2023). Efficiency of five samplers to trap suspended particulate matter and microplastic particles of different sizes. Chemosphere.

[12] Przibilla A, Iwainski S, Zimmermann T, Pröfrock D (2023). Impact of storage temperature and filtration method on dissolved trace metal concentrations in coastal water samples. Water Environ Res.

[13] Dendievel A-M, Grosbois C, Ayrault S, Evrard O, Coynel A, Debret M, Gardes T, Euzen C, Schmitt L, Chabaux F, Winiarski T, Van Der Perk M, Mourier B (2022). Key factors influencing metal concentrations in sediments along Western European rivers: a long-term monitoring study (1945–2020). Sci Total Environ.

[14] Romero-Freire A, Santos-Echeandía J, Neira P, Cobelo-García A. Less-studied technology-critical elements (Nb, Ta, Ga, In, Ge, Te) in the marine environment: review on their concentrations in water and organisms. Front Mar Sci. 2019;6. 10.3389/fmars.2019.00532.

[15] Free G, Van DBW, Gawlik B, Van WL, Wood M, Guagnini E, Koutelos K, Annunziato A, Grizzetti B, Vigiak O, Gnecchi M, Poikane S, Christiansen T, Whalley C, Antognazza F, Zerger B, Hoeve R-J, Stielstra H (2023). An EU analysis of the ecological disaster in the Oder River of 2022. JRC Publ Repos.

[16] Wiederhold J, Buchinger S, Düster L, Fischer H, Hahn J, Helms M, Hermes N, Jewell K, Kleinteich J, Krenek S, Löffler D, Mora D, Rademacher S, Schlüsener M, Schütze K, Wahrendorf D-S, Wick A, Ternes T (2023). Untersuchungen zum Fischsterben in der Oder im August 2022: BfG-Bericht 2143. Bundesanstalt für Gewässerkunde, Koblenz.

[17] Belkouteb N, Schroeder H, Arndt J, Wiederhold JG, Ternes TA, Duester L (2023). Quantification of 68 elements in river water monitoring samples in single-run measurements. Chemosphere.

[18] Pröfrock D, Prange A (2012). Inductively coupled plasma-mass spectrometry (ICP-MS) for quantitative analysis in environmental and life sciences: a review of challenges, solutions, and trends. Appl Spectrosc.

[19] Thomas R. Practical guide to ICP-MS: a tutorial for beginners (Third edition). CRC Press, Boca Raton. 2013. 10.1201/b14923.

[20] Van Acker T, Theiner S, Bolea-Fernandez E, Vanhaecke F, Koellensperger G (2023). Inductively coupled plasma mass spectrometry. Nat Rev Methods Primer.

[21] U.S. EPA. 1994. Method 200.2: Sample preparation procedure for spectrochemical determination of total recoverable elements. 1994, Revision 2.8. Washington, DC. https://www.epa.gov/sites/default/files/2015-08/documents/method_200-2_rev_2-8_1994.pdf.

[22] U.S. EPA. 2007. Method 3015A (SW-846): Microwave assisted acid digestion of aqueous samples and extracts. 2007, Revision 1. Washington, DC. https://www.epa.gov/sites/default/files/2015-12/documents/3015a.pdf.

[23] ISO 15587. Water quality - digestion for the determination of selected elements in water. Part 1: Aqua regia digestion. German Institute for Standardization; 2002. https://www.iso.org/standard/31354.html.

[24] Duester L, Hartmann LM, Luemers L, Hirner AV (2007). Particle size distribution of organometal(loid) compounds in freshwater sediments. Appl Organomet Chem.

[25] DIN-38402–30. German standard methods for the examination of water, waste water and sludge - General information (group A) - Part 30: Pretreatment, homogenization and aliquotation of non-homogeneous water samples (A 30). German Institute for Standardization; 1998. https://www.beuth.de/en/standard/din-38402-30/5164982.

[26] Sastre J, Sahuquillo A, Vidal M, Rauret G (2002). Determination of Cd, Cu, Pb and Zn in environmental samples: microwave-assisted total digestion versus aqua regia and nitric acid extraction. Anal Chim Acta.

[27] Gaudino S, Galas C, Belli M, Barbizzi S, de Zorzi P, Jaćimović R, Jeran Z, Pati A, Sansone U (2007). The role of different soil sample digestion methods on trace elements analysis: a comparison of ICP-MS and INAA measurement results. Accreditation Qual Assur.

[28] Balaram V, Subramanyam KSV (2022) Sample preparation for geochemical analysis: strategies and significance. Adv Sample Prep. 1. 10.1016/j.sampre.2022.100010.

[29] Roje V (2010). Multi-elemental analysis of marine sediment reference material MESS-3: one-step microwave digestion and determination by high resolution inductively coupled plasma-mass spectrometry (HR-ICP-MS). Chem Pap.

[30] Trimmel S, Meisel TC, Lancaster ST, Prohaska T, Irrgeher J (2023). Determination of 48 elements in 7 plant RMs by ICP-MS/MS with a focus on technology-critical elements. Anal Bioanal Chem.

[31] Dersch G, Krause WJ (2004). Monitoring and prediction of the dispersion of radioactive substances in German Federal waterways – concepts and methods. Kerntechnik.

[32] Louie H, Wong C, Huang YJ, Fredrickson S (2012). A study of techniques for the preservation of mercury and other trace elements in water for analysis by inductively coupled plasma mass spectrometry (ICP-MS). Anal Methods.

[33] da Silva YJAB, do Nascimento CWA, Biondi CM (2014). Comparison of USEPA digestion methods to heavy metals in soil samples. Environ Monit Assess.

[34] Knapp JLA, von Freyberg J, Studer B, Kiewiet L, Kirchner JW (2020). Concentration–discharge relationships vary among hydrological events, reflecting differences in event characteristics. Hydrol Earth Syst Sci.

