# Supplementary material for: Multi-element analysis of unfiltered samples in river water monitoring—digestion and single-run analyses of 67 elements
Source: Anal Bioanal Chem. 2024 Apr 6;416(13):3205–22. doi: 10.1007/s00216-024-05270-4 (PMC11068683; doi:10.1007/s00216-024-05270-4)
Supplement: Supplementary file 1 — Supplementary file1 (PDF 5591 KB) [file 216_2024_5270_MOESM1_ESM.pdf]

Supporting Information

Multi-element analysis of unfiltered samples in river water monitoring – digestion and single-run analyses of 67 elements

Nadine Belkouteb[a], Henning Schroeder[a], Jan G. Wiederhold[a], Thomas A. Ternes[a] and Lars Duester[a,*]

[a]*Federal Institute of Hydrology, Division G – Qualitative hydrology, Am Mainzer Tor 1, 56068 Koblenz, Germany*

**corresponding author: duester@bafg.de, + 49-261-1306-5275, Am Mainzer Tor 1, 56068 Koblenz, Germany*

Content

1. Calibration 2
2. Certified reference materials used to simulate whole water samples 4
3. XRF data 10
4. Aliquotation..........11
5. Sample and reagent volume..........12
6. Concentration of suspended particulate matter (SPM) 15
7. Concentrating the whole water sample – XpressVap 18
8. Additional data of section 3 of the manuscript 20
   8.1 Matrix correction prior to ICP-QQQ-MS analysis 20
   8.2 Open vs. closed-vessel 21
   8.3 Digestion reagent 25
   8.4 Time and temperature 30
9. Case study..........47
   9.1 Quality control..........47
   9.2 Rhine in Koblenz 53
   9.3 Moselle in Koblenz 55
   9.4 Rhine in Wesel 58
   9.5 Rhine in Weil 60
10. References 62

Number of pages: 62

Number of figures: 14

Number of tables: 15

1. Calibration

Table S1: Calibration ranges of all 67 elements for ICP-QQQ-MS measurement. Three calibration series were applied according to Belkouteb et al. (2023): i) HNO_3 stabilised elements, ii) HCl stabilised elements, and iii) non-metals out of four stock solutions.

			Concentration of the calibration solutions in µg/l										
Calibration series	Mass	Element	No. 1	No. 2	No. 3	No. 4	No. 5	No. 6	No. 7	No. 8	No. 9	No. 10	Stock
i	7	Li	0.0025	0.005	0.025	0.05	0.25	0.5	2.5	5	25	50	500
i	9	Be	0.001	0.002	0.01	0.02	0.1	0.2	1	2	10	20	200
i	11	B	0.015	0.03	0.15	0.3	1.5	3	15	30	150	300	3,000
i	23	Na	2.5	5	25	50	250	500	2,500	5,000	25,000	50,000	100,000
i	24	Mg	1	2	10	20	100	200	1,000	2,000	10,000	20,000	40,000
i	27	Al	1.25	2.5	12.5	25	125	250	1,250	2,500	12,500	25,000	50,000
i	28	Si	0.5	1	5	10	50	100	500	1,000	5,000	10,000	100,000
i	39	K	0.5	1	5	10	50	100	500	1,000	5,000	10,000	20,000
i	43	Ca	5	10	50	100	500	1,000	5,000	10,000	50,000	100,000	200,000
i	45	Sc	0.0005	0.001	0.005	0.01	0.05	0.1	0.5	1	5	10	100
i	47	Ti	0.01	0.02	0.1	0.2	1	2	10	20	100	200	2,000
i	51	V	0.005	0.01	0.05	0.1	0.5	1	5	10	50	100	1,000
i	52	Cr	0.005	0.01	0.05	0.1	0.5	1	5	10	50	100	1,000
i	55	Mn	0.025	0.05	0.25	0.5	2.5	5	25	50	250	500	1,000
i	56	Fe	0.5	1	5	10	50	100	500	1,000	5,000	10,000	20,000
i	59	Co	0.005	0.01	0.05	0.1	0.5	1	5	10	50	100	1,000
i	60	Ni	0.005	0.01	0.05	0.1	0.5	1	5	10	50	100	1,000
i	63	Cu	0.01	0.02	0.1	0.2	1	2	10	20	100	200	2,000
i	66	Zn	0.05	0.1	0.5	1	5	10	50	100	500	1,000	10,000
i	71	Ga	0.001	0.002	0.01	0.02	0.1	0.2	1	2	10	20	200
i	72	Ge	0.0005	0.001	0.005	0.01	0.05	0.1	0.5	1	5	10	100
i	75	As	0.005	0.01	0.05	0.1	0.5	1	5	10	50	100	1,000
i	78	Se	0.001	0.002	0.01	0.02	0.1	0.2	1	2	10	20	200
i	85	Rb	0.005	0.01	0.05	0.1	0.5	1	5	10	50	100	1,000
i	88	Sr	0.25	0.5	2.5	5	25	50	250	500	2,500	5,000	50,000
i	89	Y	0.0005	0.001	0.005	0.01	0.05	0.1	0.5	1	5	10	100
i	93	Nb	0.0005	0.001	0.005	0.01	0.05	0.1	0.5	1	5	10	100
i	95	Mo	0.005	0.01	0.05	0.1	0.5	1	5	10	50	100	1,000
i	107	Ag	0.001	0.002	0.01	0.02	0.1	0.2	1	2	10	20	200
i	111	Cd	0.0025	0.005	0.025	0.05	0.25	0.5	2.5	5	25	50	500
i	115	In	0.0005	0.001	0.005	0.01	0.05	0.1	0.5	1	5	10	100
i	125	Te	0.0005	0.001	0.005	0.01	0.05	0.1	0.5	1	5	10	100
i	133	Cs	0.0025	0.005	0.025	0.05	0.25	0.5	2.5	5	25	50	500
i	137	Ba	0.025	0.05	0.25	0.5	2.5	5	25	50	250	500	5,000
i	139	La	0.001	0.002	0.01	0.02	0.1	0.2	1	2	10	20	200
i	140	Ce	0.001	0.002	0.01	0.02	0.1	0.2	1	2	10	20	200
i	141	Pr	0.0005	0.001	0.005	0.01	0.05	0.1	0.5	1	5	10	100
i	146	Nd	0.001	0.002	0.01	0.02	0.1	0.2	1	2	10	20	200

Table S1 continued.

Calibration series	Mass	Element	Concentration of the calibration solutions in µg/l										
			No. 1	No. 2	No. 3	No. 4	No. 5	No. 6	No. 7	No. 8	No. 9	No. 10	Stock
i	147	Sm	0.0005	0.001	0.005	0.01	0.05	0.1	0.5	1	5	10	100
i	153	Eu	0.0005	0.001	0.005	0.01	0.05	0.1	0.5	1	5	10	100
i	157	Gd	0.0005	0.001	0.005	0.01	0.05	0.1	0.5	1	5	10	100
i	159	Tb	0.0005	0.001	0.005	0.01	0.05	0.1	0.5	1	5	10	100
i	163	Dy	0.0005	0.001	0.005	0.01	0.05	0.1	0.5	1	5	10	100
i	165	Ho	0.0005	0.001	0.005	0.01	0.05	0.1	0.5	1	5	10	100
i	166	Er	0.0005	0.001	0.005	0.01	0.05	0.1	0.5	1	5	10	100
i	169	Tm	0.0005	0.001	0.005	0.01	0.05	0.1	0.5	1	5	10	100
i	172	Yb	0.0005	0.001	0.005	0.01	0.05	0.1	0.5	1	5	10	100
i	175	Lu	0.0005	0.001	0.005	0.01	0.05	0.1	0.5	1	5	10	100
i	181	Ta	0.0005	0.001	0.005	0.01	0.05	0.1	0.5	1	5	10	100
i	182	W	0.0005	0.001	0.005	0.01	0.05	0.1	0.5	1	5	10	100
i	205	Tl	0.0025	0.005	0.025	0.05	0.25	0.5	2.5	5	25	50	500
i	208	Pb	0.025	0.05	0.25	0.5	2.5	5	25	50	250	500	5,000
i	209	Bi	0.001	0.002	0.01	0.02	0.1	0.2	1	2	10	20	200
i	232	Th	0.001	0.002	0.01	0.02	0.1	0.2	1	2	10	20	200
i	238	U	0.0015	0.003	0.015	0.03	0.15	0.3	1.5	3	15	30	300
ii	90	Zr	0.001	0.002	0.01	0.02	0.1	0.2	1	2	10	20	200
ii	101	Ru	0.0005	0.001	0.005	0.01	0.05	0.1	0.5	1	5	10	100
ii	118	Sn	0.0025	0.005	0.025	0.05	0.25	0.5	2.5	5	25	50	500
ii	121	Sb	0.0025	0.005	0.025	0.05	0.25	0.5	2.5	5	25	50	500
ii	178	Hf	0.0005	0.001	0.005	0.01	0.05	0.1	0.5	1	5	10	100
ii	193	Ir	0.0005	0.001	0.005	0.01	0.05	0.1	0.5	1	5	10	100
ii	195	Pt	0.0005	0.001	0.005	0.01	0.05	0.1	0.5	1	5	10	100
ii	197	Au	0.0005	0.001	0.005	0.01	0.05	0.1	0.5	1	5	10	100
ii	202	Hg	0.0005	0.001	0.005	0.01	0.05	0.1	0.5	1	5	10	100
iii	31	P	0.1	0.2	1	2	10	20	100	200	1,000	2,000	4,000
iii	32	S	2.5	5	25	50	250	500	2,500	5,000	25,000	50,000	100,000
iii	79	Br	0.25	0.5	2.5	5	25	50	250	500	2,500	5,000	10,000

2. Certified reference materials used to simulate whole water samples

Table S2: Reference and indicative values (italic font) in mg/kg of the eight reference materials used in our study.

	Reference is aqua regia digestion (except for CRM-MS-S*)								Reference is total digestion							
	**CRM-MS-S**	**Metranal-18**	**Metranal-1**	**NWHR-1**	**NWWQB-1**	**BCR 667**	**JSd-3**	**SdAR-M2**	**CRM-MS-S**	**Metranal-18**	**Metranal-1**	**NWHR-1**	**NWWQB-1**	**BCR 667**	**JSd-3**	**SdAR-M2**
Li	55.9				39.1			12.9							*151*	18.1
Be	*1.2*	1.7	1.58	0.630	1.10			4.70			2.65				*9.08*	6.6
B	33.3							*20.0*								
Na	16,700				295			*497*								19,200
Mg	9,220			11,100	9,330											*2,960*
Al	41,900				24,500											66,000
Si																343,000
P	*1,200*							*389*								*445*
S															*399*	
K	3,620							*2,490*								41,500
Ca	35,800	57,276		60,400	9,430			2,930								6,000
Sc								1.91						13.70	*10.5*	4.08
Ti	504							*384*								1,800
V	65	56.6	43.2		46.1			*14.9*			73.0				*70.4*	25.2
Cr	59.4	48.3	93.0	101	46.8			*7.9*			118			178	*35.3*	*51*
Mn	378	375	1,330	410	2240			914			1,370			920		1,040
Fe	33,500			24,100	43,200			*15,300*						44,800		18,400
Co	8.62	11.8	12.5	9.44	16.5			12.8			15.6			23.0	*12.7*	12.4
Ni	20.5	32.6	35.4	33.0	57.6			47.0			45.0			128	*19.6*	48.8
Cu	34.4	98.2	91.2	72.9	74			245			97.0			60	*426*	*239*
Zn	133	920	465	1,072	258			792			520			175	*136*	772

Table S2 continued.

Ga							*3.21*					*13.5*	*17.6*
Ge													
As	12.1	35.0	24.1	5.58	20.3		84		29.0		14.3	*252*	*80*
Se			0.74		1.20		*3.8*		1.02		1.59	*1.29*	
Br											99.70	*3.90*	
Rb							15.8					*285*	149
Sr	146			103	36.3		18.8				206	*58.7*	144
Y							15.4				16.7	*14.9*	32.7
Zr							*4.8*					*124*	*259*
Nb							*3.6*					*7.8*	26.2
Mo			0.97	1.00	1.19		13.4		1.13				*13.1*
Ru													
Ag	*0.3*	2.90	2.50	4.74	0.757		*16.3*		3.10			*3.38*	
Cd	*0.3*	2.2	2.66	3.69	1.72		5.11		3.10		0.67	*1.045*	5.1
In							2.06						
Sn		13.7	7.37	6.84			1.23		8.75			*195*	*2.4*
Sb	*0.3*		1.73				*99*		2.52		0.96	*2.78*	*111*
Te							1.91						
Cs							0.79				7.8	*30.6*	1.8
Ba	44.6	64.9	275	119	260		109		522			*462*	990
La							41.1				27.8	*19.8*	46.6
Ce							90				56.70	*42.0*	100
Pr											6.1	*3.09*	11
Nd											25	*15.7*	40
Sm							*5.8*				4.66	*3.26*	7.18
Eu							*0.58*				1	*0.686*	1.44
Gd							*4.6*				4.41	*2.63*	6.3

Table S2 continued.

Tb						*0.60*		0.682	*0.368*	0.95
Dy						*3.5*		4.01	*2.22*	5.88
Ho						*0.62*		0.8	*0.443*	1.21
Er						*1.83*		2.35	*1.07*	3.58
Tm								0.326	*0.155*	0.54
Yb						1.56		2.2	*1.4*	*3.5*
Lu						0.208		0.325	*0.196*	0.54
Hf						0.15			*3.21*	7.3
Ta								0.876	*0.687*	1.76
W						1.10			*179*	3.45
Ir										
Pt									*0.0013*	
Au								0.0153	*0.0056*	
Hg			1.19	0.331	1.06	1.40	1.30		*0.254*	*1.4*
Tl					0.737	1.94				*2.9*
Pb	74.5	170	82.4	131	79.0	800	93.2	31.9	*82.1*	808
Bi			0.69			1.03	0.72		*23.8*	1.05
Th						*11.3*		10	*7.79*	14.2
U					3.00	1.46		2.26	*1.66*	2.53

*** Reference values for CRM-MS-S are for HNO_3/H_2O_2 digestion according to US EPA 3050B.**

Table S 3: Absolute standard deviation in mg/kg of the reference and indicative values for the eight reference materials used in our study. For JSd-3 no standard deviation was given.

	**Reference is aqua regia digestion (except for CRM-MS-S*)**								**Reference is total digestion**							
	**CRM-MS-S**	**Metranal-18**	**Metranal-1**	**HR-1**	**WQB-1**	**BCR 667**	**JSd-3**	**SdAR-M2**	**CRM-MS-S**	**Metranal-18**	**Metranal-1**	**HR-1**	**WQB-1**	**BCR 667**	**JSd-3**	**SdAR-M2**
Li	5.6				16.6			0.8								0.8
Be		0.1	0.32	0.319	0.65			0.25			0.8					0.2
B	4.1							1.3								
Na	1,000				98			22								223
Mg	610			2,500	1,840											*121*
Al	2,900				10,700											318
Si																795
P								28								*17*
S																
K	270							166								249
Ca	2,400	335		11,100	1,940			143								71
Sc								0.12						0.70		0.21
Ti	47			3.52				30								18
V	5	7.00	5.60		31.00			1.10			10.20					0.90
Cr	5.0	2.50	14	21	11.60			0.40			12.00					3
Mn	24	12	106	95	393			39			140					15
Fe	2,200			6,540	9,500			490								140
Co	0.61	1.0	1.5	1.8	3.2			0.50			1.4					0.5
Ni	2.1	3.9	5	5.9	10			1.6			8.2					1.3
Cu	4.1	3.1	8	14.4	11.9			6			10					6
Zn	13	30	68	184	50			20			68					19
Ga								0.43								0.40
Ge																

Table S3 continued.

As	2.2	2.9	3.2	1.71	5.2	3	2		3
Se			0.14		0.67	0.60	0.20		
Br									
Rb						0.9			3
Sr	10			21	9.7	0.7			2
Y						0.7			0.7
Zr						0.6			8
Nb						0.6			0.9
Mo			0.02	0.37	0.59	0.6	0.12		0.3
Ru									
Ag		0.14	0.32	1.26	0.278	1.9	0.38		
Cd		0.1	0.54	0.66	0.41	0.12	0.68		0.3
In						0.11			
Sn		0.3	1.48			0.14	1.74		0.3
Sb			0.34			7	0.5		5
Te						0.17			
Cs						0.07			0.06
Ba	3.9	12.0	22.0	29.0	50.0	5	104.0		15
La						1.7		1	1.3
Ce						6		2.50	2
Pr								0.5	0.2
Nd								1.4	0.6
Sm						0.5		0.2	0.15
Eu						0.08		0.05	0.05
Gd						0.6		0.12	0.2
Tb						0.07		0.017	0.07
Dy						0.5		0.14	0.13

Table S3 continued.

Ho						0.06		0.06	0.03
Er						0.25		0.15	0.14
Tm								0.025	0.02
Yb						0.17		0.09	0.1
Lu						0.012		0.02	0.02
Hf						0.02			0.3
Ta									0.12
W						0.12			0.3
Ir									
Pt									
Au									
Hg			0.15	0.096	0.25	0.14	0.1		0.1
Tl					0.293	0.16			0.3
Pb	6.5	4	16.4	15	9.5	20	10		17
Bi			0.14			0.08	0.14		0.04
Th						1.2		0.5	0.4
U					1.08	0.11		0.15	0.1

*** Reference values for CRM-MS-S are for HNO_3/H_2O_2 digestion according to US EPA 3050B.**

3. XRF data

Table S 4: Concentrations in mg/kg measured with XRF for all eight CRMs used in this study.

	CRM-MS-S	Metranal-18	Metranal-1	NWHR-1	NWWQB-1	BCR 667	JSd-3	SdAR-M2
Na	10,400	192	1,460	4,730	973	9,580	142	16,200
Mg	13,000	6,200	8,030	15,600	14,400	24,500	8,130	3,250
Al	64,600	58,300	53,000	57,200	77,700	69,500	58,000	53,800
Si	189,000	177,000	250,000	217,000	233,000	195,000	301,000	315,000
P	735	1,810	2,830	998	870	1,090	275	242
S	5,780	8,120	3,340	1,480	1,600	4,660	239	760
Cl	10,300	188	131	76.5	71.5	10,100	14.7	60
K	8,360	11,900	14,600	19,700	27,800	20,000	16,300	38,700
Ca	30,600	46,700	22,300	61,400	9,960	40,100	3,680	5,490
Ti	3,900	3,930	3,330	4,000	4,290	3,350	2,280	1,620
V	90.1	99.7	81	72	135	113	76.9	23
Cr	95.4	85.2	123	156	99.2	180	33.3	28.4
Mn	380	400	1,430	518	2,050	850	972	1,030
Fe	37,700	30,000	28,700	33,300	52,000	46,900	29,200	17,900
Co	<1.0	<1.0	<1.0	<1.0	<1.0	<1.0	<1.0	<1.0
Ni	25.8	42.9	40.9	42	65.9	123	21.3	47
Cu	32.4	99.7	83.9	78.5	76.7	58.9	388	221
Zn	135	924	461	1,140	276	166	129	748
Ga	14	14.5	12	15.3	22.4	17.5	11.1	16.2
Ge	1.2	2.9	2.3	1.1	2.6	1.5	5.1	1.1
As	13.2	34.1	28.4	6.5	24.6	17.2	255	76
Se	0.6	0.9	1.2	<1.0	1	0.4	1.4	2.5
Br	139	7.6	7	6.9	39	87.1	4.7	1.6
Rb	51.9	74.1	85.6	77.5	144	123	268	144
Sr	178	204	168	250	132	213	52.2	137
Y	21.7	25.9	24.8	26.3	31.9	23	11.3	33.6
Zr	176	214	276	230	150	112	118	253
Nb	12.5	17.5	13.8	12.3	14.6	12.1	7.6	23.7
Mo	1.8	2.3	0.9	1.4	1.9	0.7	1.2	15.3
Ag	3.1	3.1	2.2	4.5	4.8	3.9	2.9	14.3
Cd	<0.2	2.5	2.8	3.8	1.5	0.7	1.3	4.9
Sb	<1.0	8.9	1.3	1.4	<1.0	<1.0	1.2	96.3
Sn	6.2	16.1	10.3	9.1	3.7	2.6	186	0.9
Ba	223	586	513	495	556	290	459	954
Hg	<1.0	<1.0	1.3	<1.0	0.6	<1.0	<1.0	1.2
Tl	0.5	1.6	0.8	0.5	1.3	0.6	2	3.1
Bi	<0.3	0.8	0.6	<0.3	<0.3	<0.3	12.8	3.5
Pb	63	186	89.7	147	86.7	34.3	83.2	819

4. Aliquotation

The aliquotation procedure was not tested in detail in this study but Figure S 1 shows that the overall relative standard deviation (RSD) of five replicates was below 10% for 83% of the elements measured above LOQ in a river water sample from the river Rhine (Koblenz) with a SPM concentration of 22.4 mg/l. The RSD did not exceed 30% for any element. This indicates that the simple aliquotation procedure described in section 2.4 of the manuscript together with the uncertainty of the digestion and measurement procedures resulted in a sufficiently high reproducibility. In general, aliquotation of whole water samples may lead to uncertainties between samples due to an inhomogeneous fractionation of particles. This is especially the case for whole water samples that contain a low amount of suspended particulate matter or only a few larger particles containing a certain analyte. In a worst-case scenario, if one particle occurs only once in a row of five replicate samples exhibiting very low SPM concentrations, strongly varying results for the element would be achieved.

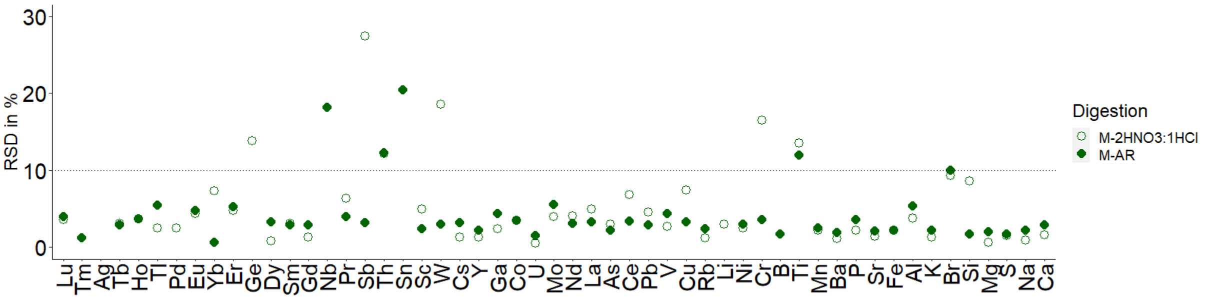

Figure S 1: RSD in % for all elements measured by ICP-QQQ-MS after microwave (M) aqua regia (M-AR) and the optimised microwave digestion approach (M-2HNO$_3$:1HCl) at 150 °C and 20 min in five replicate samples of the River Rhine (Koblenz, SPM: 22.4 mg/l). Elements below LOQ are not depicted: Be, Zn, Se, Ru, Cd, In, Te, Hf, Ta, Ir, Pt, Au, Hg and Bi. The x-axis is ordered from low (ng/l) to high concentration (mg/l) elements.

5. Sample and reagent volume

Looking at the elements in Figure S 2 as examples for analytes regularly analysed and of continuous interest, it is clear that there is no systematic reduction of the digestion efficiency with higher water volumes. The additional data is presented in Table S 5.

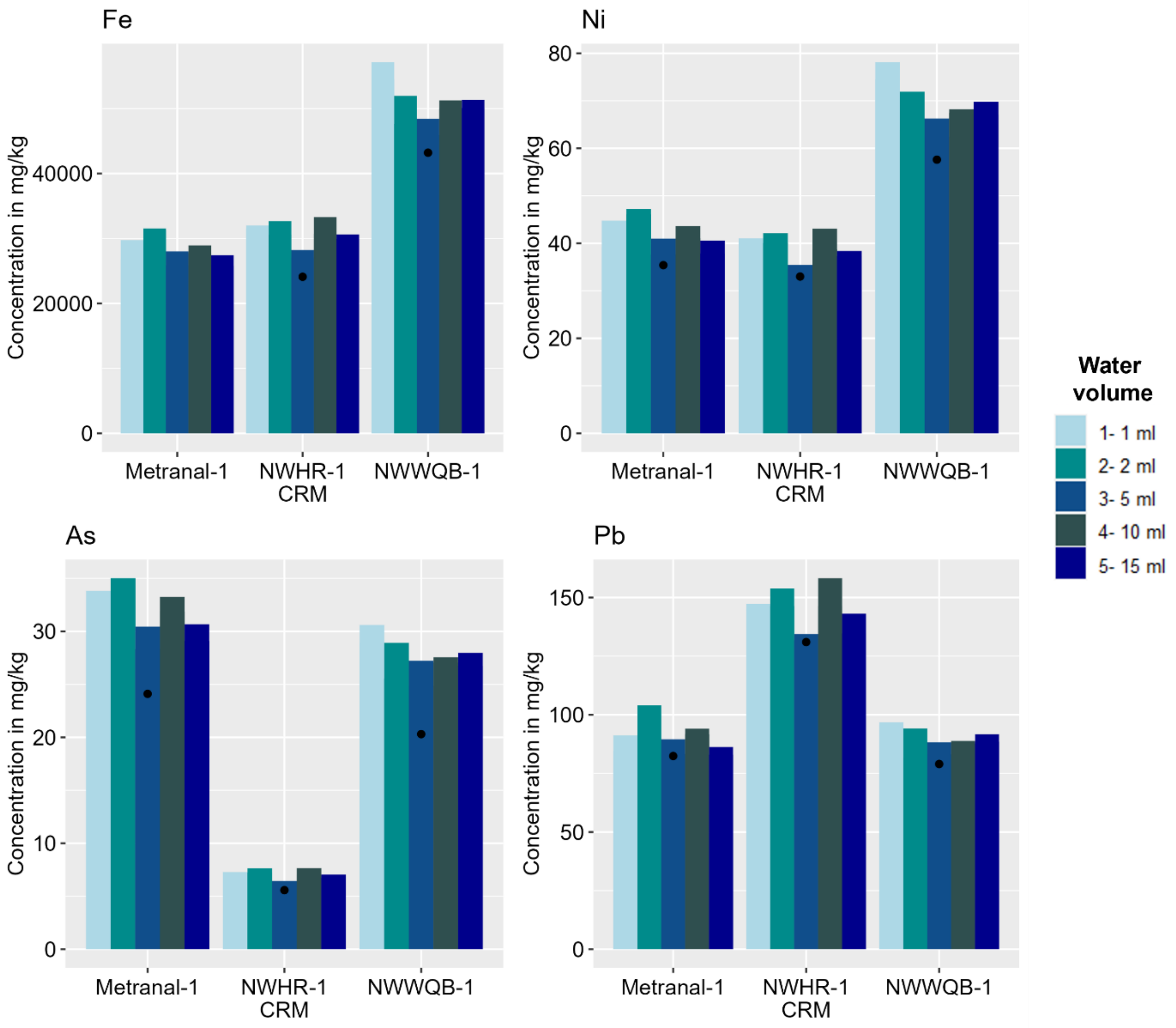

Figure S 2: Mean concentrations of Fe, Ni, As, Pb in mg/kg in sediment CRMs (0.3 g, duplicates) and different added water volumes (1, 2, 5, 10, 15 ml) for suspension using aqua regia. The points indicate the certified aqua regia reference values.

Table S 5: Concentrations in mg/kg in sediment CRMs (0.3 g) and added water volumes of 1, 2, 5, 10 and 15 ml.

CRM	Water volume	Li	Be	B	Na	Mg	Al	K	Ca	Sc	Ti	V	Cr	Mn	Fe	Co	Ni	Cu	Zn	Ga	As	Se	Sr	Y	Mo	Ag
Metranal-1	1- 1 ml	25.7	2.16	33.8	462	5,220	27,600	5,520	31,200	5.88	1,150	57.8	101	1,310	26,700	13.3	39.1	87.1	473	7.99	29.6	1.26	131	15	1.07	2.54
Metranal-1	1- 1 ml	28	2.31	36.2	525	5,780	30,600	6,300	35,100	6.74	1,320	65.8	116	1,450	29,700	14.9	44.8	98.7	519	8.94	33.8	1.41	148	16.7	1.25	2.87
Metranal-1	2- 2 ml	28	2.46	36.9	504	5,810	32,500	6,050	37,100	6.58	1,280	66	117	1,570	31,500	15.8	47.2	105	552	9.67	35	1.51	153	17.3	1.2	2.97
Metranal-1	2- 2 ml	28.9	2.37	37.5	523	5,770	31,600	6,440	36,200	6.81	1,350	67.7	118	1,540	31,300	15.7	46.1	105	546	9.32	35	1.65	151	17.4	1.29	3.54
Metranal-1	3- 5 ml	25.3	2.11	31.6	448	5,010	26,900	5,450	31,300	5.66	1,160	57.2	100	1,370	28,000	13.8	41	90.9	508	8.04	30.4	1.29	131	14.9	1.15	2.74
Metranal-1	3- 5 ml	23.4	1.92	29.5	424	4,670	24,700	5,280	29,300	5.44	1,110	53.8	93.2	1,260	25,800	12.7	37.6	84.9	457	7.44	28.3	1.22	122	14.3	1.03	2.55
Metranal-1	4- 10 ml	25.4	2.12	32.7	471	5,000	27,600	5,970	32,900	6.09	1,230	61.4	104	1,360	27,700	13.9	41.1	90.8	492	8.55	31.8	1.37	138	15.8	1.33	2.8
Metranal-1	4- 10 ml	27	2.2	34.9	520	5,270	30,400	6,620	33,500	6.63	1,390	65.4	110	1,420	28,900	14.4	43.6	95.4	509	9.17	33.2	1.35	143	16.9	1.22	2.74
Metranal-1	5- 15 ml	25.7	2.03	31.3	507	5,080	27,700	6,110	32,200	6.12	1,180	60.3	103	1,360	27,400	13.6	40.6	89	487	8.3	30.7	1.3	137	15.6	1.07	2.86
Metranal-1	5- 15 ml	24.1	1.95	29.9	480	4,830	27,000	5,900	30,600	5.84	1,150	57.2	96.4	1,260	25,800	12.8	37.9	82.9	459	8.17	29.1	1.25	129	14.6	1.05	2.58
NWHR-1	1- 1 ml	25.8	1.22	35.1	570	14,200	33,600	8,840	95,100	9.25	1,470	71.5	146	557	31,400	11.8	40.4	81.1	1,160	10.2	7.28	0.68	139	21.6	1.34	5.45
NWHR-1	1- 1 ml	25.9	1.18	33.1	524	13,900	31,700	8,000	95,000	8.76	1,390	67.4	140	564	32,000	12	41	83.3	1,190	9.64	7.25	0.67	135	21	1.35	5.64
NWHR-1	2- 2 ml	26.5	1.18	33	533	14,200	31,900	8,350	1E+05	9.25	1,450	70.2	149	579	32,700	12.3	42.1	85.4	1,230	9.9	7.64	0.69	143	22.5	1.35	5.81
NWHR-1	2- 2 ml	24.2	1.11	30.3	496	13,600	28,500	7,580	93,500	8.29	1,350	65.2	137	547	30,500	11.6	40	81.3	1,150	8.85	7.22	0.66	134	20.8	1.25	5.42
NWHR-1	3- 5 ml	22	0.95	25.6	418	11,700	25,600	6,390	79,400	6.79	1,090	53	115	479	26,900	10	34.5	71.3	1,050	8.15	6.19	0.6	114	17.9	1.14	5.19
NWHR-1	3- 5 ml	22.3	1.01	29.1	491	12,200	29,600	7,930	81,600	7.77	1,280	60.8	121	502	28,200	10.3	35.5	72.2	1,070	9.28	6.44	0.61	120	18.8	1.17	5
NWHR-1	4- 10 ml	27	1.21	34.9	578	13,900	34,600	9,240	99,600	9.46	1,470	73	149	584	33,300	12.4	43.1	85.7	1,250	10.8	7.66	0.69	147	22.7	1.4	5.88
NWHR-1	4- 10 ml	24.2	1.12	32	564	12,800	33,200	8,950	90,700	8.68	1,400	68.3	132	537	30,700	11.3	38.7	78.3	1,130	10.5	7.03	0.64	135	20.8	1.27	5.54
NWHR-1	5- 15 ml	23.6	1.06	31	587	13,000	31,500	8,600	88,200	8.11	1,280	65	127	529	29,900	10.9	37.3	75.7	1,120	9.66	6.74	0.63	132	19.9	1.17	5.25
NWHR-1	5- 15 ml	23.8	1.09	30.9	610	13,500	32,900	8,930	92,400	8.52	1,330	66.9	132	532	30,600	11.2	38.4	77.1	1,150	10.3	7.04	0.65	137	20.9	1.24	5.38
NWWQB-1	1- 1 ml	57.9	2.48	83.9	649	13,900	68,300	18,100	15,800	14	1,010	123	91.9	2,730	54,500	21.5	74.4	90.9	306	18.8	28.9	1.39	79.2	26.3	1.6	0.87
NWWQB-1	1- 1 ml	62.1	2.71	99.9	722	15,000	74,400	20,300	16,600	15.4	1,120	136	99.3	2,860	57,100	22.4	78.1	94.3	317	20	30.6	1.47	86.3	27.8	1.83	0.9
NWWQB-1	2- 2 ml	54.3	2.22	70.2	584	13,300	60,100	16,200	15,700	13.1	923	114	85.7	2,640	52,000	20.6	71.9	88	296	17.1	28.9	1.4	73.6	26.3	1.56	0.84
NWWQB-1	2- 2 ml	50.4	2.03	73.8	585	12,700	58,300	16,300	13,800	12.5	886	108	78.9	2,410	47,600	18.5	63.8	78.2	269	16.1	25.5	1.25	70.4	23.3	1.4	0.79
NWWQB-1	3- 5 ml	46.6	1.91	66.7	525	11,600	52,800	14,900	13,500	11.5	843	100	74.3	2,320	45,900	18	62.4	76.6	262	15.1	24.8	1.2	65.6	22.2	1.43	0.78
NWWQB-1	3- 5 ml	49	1.98	61.8	531	12,400	53,800	14,600	14,500	11.9	867	102	78	2,480	48,400	19.1	66.3	81.5	278	15.6	27.2	1.32	67.1	24.1	1.55	0.79
NWWQB-1	4- 10 ml	52.7	2.01	72.5	624	13,000	59,000	17,000	15,000	12.8	951	112	81.4	2,540	50,000	19.5	67.4	83	283	16.9	27.5	1.32	74.2	24.9	1.51	0.84
NWWQB-1	4- 10 ml	54.3	2.26	86	707	13,200	74,600	19,600	14,700	14.1	1,080	124	86.4	2,540	51,300	19.7	68.2	82.9	283	20.7	27	1.28	80.4	24.9	1.54	0.83
NWWQB-1	5- 15 ml	52.8	2.04	67.8	657	12,800	60,100	16,500	15,400	12.7	934	110	79.7	2,620	51,300	20.1	69.8	85.6	292	17.5	28	1.35	72.8	25	1.58	0.85
NWWQB-1	5- 15 ml	51.9	2.11	75.2	675	12,600	61,400	17,300	14,500	12.8	954	111	78.7	2,510	50,100	19.4	67	81.8	281	17.3	26.7	1.3	73	23.8	1.38	0.82

Table S5 continued.

CRM	Water volume	Cd	In	Sb	Te	Cs	La	Ce	Pr	Nd	Sm	Eu	Gd	Tb	Dy	Ho	Er	Tm	Yb	Lu	Hg	Tl	Pb	Th	U
Metranal-1	1- 1 ml	2.96	0.04	2.4	3.51	5.18	32.5	66.2	6.82	26.8	5.13	0.85	4.06	0.53	2.87	0.51	1.32	0.17	1.03	0.15	1.5	0.41	84.3	8.41	2.5
Metranal-1	1- 1 ml	3.26	0.04	2.57	4.1	5.89	35.3	70.3	7.5	29.6	5.66	0.97	4.54	0.59	3.22	0.57	1.46	0.19	1.16	0.16	1.71	0.45	91.2	9.08	2.62
Metranal-1	2- 2 ml	3.73	0.05	2.93	4.3	6.42	37.2	74.5	8.31	32.5	6.25	1.06	5.01	0.66	3.57	0.63	1.61	0.21	1.28	0.18	1.67	0.48	104	9.47	2.68
Metranal-1	2- 2 ml	3.42	0.05	3.03	4.34	6.46	38.1	75.9	8.37	33	6.35	1.04	5.04	0.66	3.53	0.62	1.59	0.2	1.24	0.17	1.7	0.46	99.9	10	2.67
Metranal-1	3- 5 ml	3.1	0.04	2.47	3.69	5.52	32.7	66.7	6.93	27.2	5.24	0.91	4.16	0.54	2.94	0.52	1.36	0.17	1.09	0.15	1.46	0.41	89.6	7.94	2.28
Metranal-1	3- 5 ml	2.79	0.04	2.5	3.48	5.23	32.1	63.9	6.88	26.9	5.12	0.84	4.11	0.53	2.9	0.51	1.31	0.17	1.03	0.14	1.41	0.38	83.2	8.23	2.15
Metranal-1	4- 10 ml	3.12	0.04	2.87	3.9	5.95	35.5	70.8	7.79	30.6	5.88	0.97	4.65	0.6	3.21	0.56	1.44	0.18	1.14	0.16	1.5	0.42	91.7	9.09	2.32
Metranal-1	4- 10 ml	3.14	0.07	2.6	4.11	6.25	36.7	73.9	8.11	31.8	6.03	0.98	4.8	0.62	3.4	0.6	1.55	0.2	1.22	0.17	1.52	0.44	94	9.53	2.35
Metranal-1	5- 15 ml	3	0.04	2.29	3.71	5.67	33.7	67.3	6.94	27.4	5.25	0.91	4.21	0.54	2.97	0.53	1.36	0.18	1.08	0.15	1.4	0.42	86.3	8.74	2.19
Metranal-1	5- 15 ml	2.85	0.04	2.17	3.48	5.39	33.3	66.7	6.83	26.7	5.1	0.85	4.06	0.53	2.84	0.5	1.27	0.16	1.01	0.14	1.36	0.41	82.9	8.31	2.17
NWHR-1	1- 1 ml	4.2	0.03	1.74	2.31	2.77	30	64.3	7.15	29.2	5.82	1.1	4.82	0.67	3.95	0.74	2.03	0.27	1.76	0.25	0.37	0.39	145	6.25	1.06
NWHR-1	1- 1 ml	4.2	0.04	1.8	2.2	2.7	29.5	63.8	7.19	29.5	5.93	1.1	4.89	0.68	3.94	0.74	2.02	0.27	1.75	0.25	0.37	0.37	147	6.36	1.03
NWHR-1	2- 2 ml	4.33	0.04	1.87	2.29	2.83	30.7	65.9	7.36	30.2	6.03	1.14	5.01	0.69	4.06	0.77	2.09	0.28	1.81	0.26	0.39	0.38	154	6.33	1.03
NWHR-1	2- 2 ml	4	0.04	1.78	2.11	2.62	28.6	61.4	6.91	28.3	5.72	1.07	4.69	0.65	3.79	0.72	1.94	0.26	1.67	0.24	0.37	0.34	146	5.76	0.96
NWHR-1	3- 5 ml	3.88	0.03	1.58	1.79	2.3	24.5	55.9	6	24.8	5.01	0.95	4.16	0.58	3.38	0.64	1.72	0.23	1.49	0.21	0.34	0.34	134	4.97	0.85
NWHR-1	3- 5 ml	3.87	0.03	1.64	2	2.49	28.2	60.3	6.41	26.2	5.19	0.98	4.26	0.6	3.48	0.65	1.78	0.24	1.53	0.22	0.31	0.37	132	5.3	0.89
NWHR-1	4- 10 ml	4.61	0.04	1.82	2.39	3.01	33.2	71.5	7.65	31.4	6.24	1.17	5.16	0.71	4.15	0.78	2.11	0.28	1.8	0.26	0.38	0.42	158	6.58	1.03
NWHR-1	4- 10 ml	4.3	0.03	1.74	2.27	2.83	30.4	64.7	7	28.7	5.72	1.08	4.71	0.65	3.82	0.72	1.97	0.27	1.69	0.24	0.37	0.41	146	5.7	0.92
NWHR-1	5- 15 ml	3.98	0.03	1.5	2.03	2.54	30	64.7	6.4	26.3	5.21	0.98	4.3	0.6	3.49	0.65	1.77	0.24	1.52	0.22	0.33	0.39	135	5.1	0.9
NWHR-1	5- 15 ml	4.29	0.03	1.57	2.14	2.66	31.5	67.1	6.87	28.1	5.55	1.05	4.61	0.64	3.69	0.7	1.87	0.25	1.6	0.23	0.33	0.41	143	5.5	0.92
NWWQB-1	1- 1 ml	2.03	0.09	1.64	5.31	7.17	40.1	84.4	10.3	42	8.4	1.59	6.76	0.9	5.08	0.94	2.47	0.33	2.06	0.29	1.22	1.16	95.1	10.2	4.43
NWWQB-1	1- 1 ml	2.07	0.09	1.71	5.73	7.7	43.4	90.3	11	44.3	8.82	1.66	7.04	0.94	5.31	0.98	2.57	0.34	2.15	0.31	1.27	1.21	96.8	10.9	3.77
NWWQB-1	2- 2 ml	2	0.09	1.62	4.99	6.68	37.4	79	9.74	39.9	8.1	1.53	6.52	0.88	4.96	0.9	2.38	0.31	1.97	0.28	1.22	1.1	94.1	9.51	3.43
NWWQB-1	2- 2 ml	1.76	0.08	1.38	4.41	5.98	36.9	78	8.65	35.1	7.01	1.34	5.65	0.76	4.3	0.79	2.1	0.27	1.73	0.25	1.05	1.06	81.5	8.6	3.26
NWWQB-1	3- 5 ml	1.74	0.08	1.4	4.36	6.14	34.6	72.8	8.69	35.5	7.14	1.35	5.74	0.77	4.37	0.79	2.09	0.28	1.72	0.25	1.07	0.97	83	8.45	3.09
NWWQB-1	3- 5 ml	1.91	0.08	1.46	4.55	6.15	34.1	72.9	8.7	35.8	7.33	1.39	5.95	0.79	4.46	0.82	2.16	0.28	1.77	0.25	1.16	1.03	88.3	8.13	2.62
NWWQB-1	4- 10 ml	1.96	0.08	1.41	4.59	6.38	39.3	82.8	9.06	37.1	7.5	1.42	6.02	0.8	4.52	0.82	2.16	0.29	1.77	0.25	1.12	1.15	88.8	8.72	3.55
NWWQB-1	4- 10 ml	2.1	0.08	1.4	4.98	6.91	43.1	89.8	9.67	39.1	7.69	1.44	6.09	0.81	4.58	0.84	2.21	0.29	1.82	0.26	1.09	1.32	87.7	9.27	3.54
NWWQB-1	5- 15 ml	2.07	0.08	1.26	4.59	6.44	39.4	83.5	9.12	37.4	7.65	1.45	6.18	0.82	4.58	0.84	2.19	0.28	1.78	0.25	1.13	1.18	91.7	8.59	3.1
NWWQB-1	5- 15 ml	1.95	0.08	1.26	4.55	6.44	39.8	83.4	9.04	36.9	7.43	1.4	5.97	0.79	4.43	0.81	2.12	0.28	1.72	0.24	1.07	1.15	88.5	8.59	3.65

6. Concentration of suspended particulate matter (SPM)

Figure S 3 reflects examples of different simulated whole water samples with SPM concentrations of 0.1, 0.25 and 1.0 g/l. The concentration of 0.25 g/l, also chosen as quality control in the final digestion protocol, delivered the best results regarding the certified aqua regia extractable values. The additional data is presented in Table S 6.

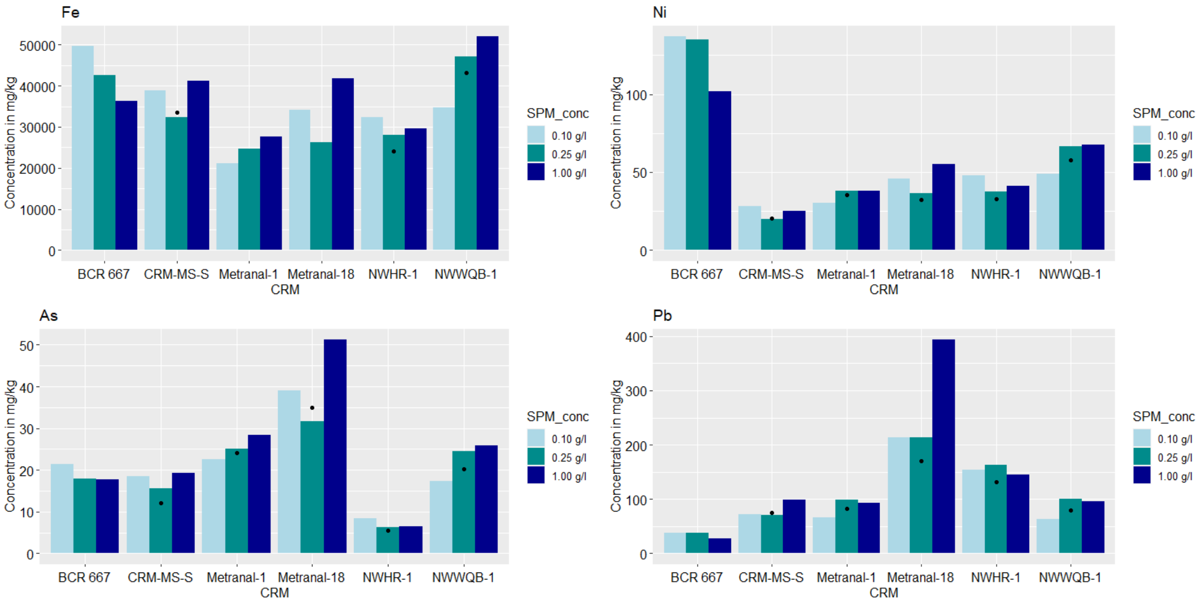

Figure S 3: Mean concentration of Fe, Ni, As and Pb in mg/kg in simulated whole water samples with different SPM concentrations using aqua regia (n=3 per CRM for 0.1 and 1 g/l and n=2 for 0.25 g/l). The points indicate the certified aqua regia extractable value.

Table S 6:Concentrations in mg/kg in simulated whole water samples with different SPM concentrations in aqua regia digests. Concentrations which are outliers (outlier detection with boxplots) but kept in the data row are marked with an asterisk (*).

CRM	SPM_conc	Li	Be	B	Na	Mg	Al	K	Ca	Sc	Ti	V	Cr	Mn	Fe	Co	Ni	Cu	Zn	Ga	As	Se	Sr	Y	Mo	Ag
CRM-MS-S	1.00 g/l	68.4	1.65	/	18,300	11,300	60,800	5,830	39,700	11	1,980	94.5	81.8	452	41,300	10.1	25.1	38	/	14.3	19.2	0.888	180	18.5	1.89	0.215
CRM-MS-S	1.00 g/l	59	1.55	/	17,700	10,600	52,500	5,170	36,900	9.8	1,590	85.2	75	418	37,600	9.13	22.8	34.1	/	12.5	18	0.832	166	17	1.92	0.199
CRM-MS-S	1.00 g/l	58.7	1.55	/	18,100	10,500	54,700	5,370	37,000	10	1,760	85.9	71.8	415	37,100	9.18	22.8	33.6	/	12.9	17.5	0.761	168	17	1.88	0.193
Metranal-18	1.00 g/l	29.5	2.23	/	1,130	4,520	31,500	7,080	54,000	7.8	1,180	78.3	63	409	28,500	12.2	37.4	104	989	9.93	34.5	1.49	171	14.1	1.91	3.31
Metranal-18	1.00 g/l	33.4	2.89	/	1,290	5,450	37,800	8,370	63,500	9.5	1,430	94.9	74.8	495	35,100	14.8	45.8	133	1,280	12.1	43.6	2.03	205	17.6	2.07	7.63
Metranal-18	1.00 g/l	42.7	3.45	/	1,540	6,570	47,800	10,300	73,000	12	1,780	114	90.6	595	41,700	17.7	55.3	162	1,520	15	51.3	2.35	249	20.9	2.65*	5.13
Metranal-1	1.00 g/l	25.5	1.93	/	1,260	5,240	26,800	6,840	23,000	5.8	1,060	55.1	91	1290	26,400	12.9	37.1	89.6	501	7.78	27.5	1.24	128	14.8	/	2.68
Metranal-1	1.00 g/l	28.6	2.12	/	1,530	5,530	30,400	7,960	24,400	6.4	1,260	61.2	96.4	1310	27,500	13.1	38	87.8	505	8.57	28.2	1.43	147	16.6	/	2.58
Metranal-1	1.00 g/l	27.8	2.1	/	1,440	5,440	29,000	7,560	23,400	6.2	1,190	58.9	95.2	1290	27,200	13	37.8	84.4	508	8.23	28.4	1.39	139	16.6	/	2.47
NWHR-1	1.00 g/l	27.5	1.17	/	2,060	13,900	33,300	10,300	63,300	8.3	1,200	62.5	122	494	29,000	10.7	37.5	77.2	1,190	9.45	6.35	0.657	140	19.4	/	5.21
NWHR-1	1.00 g/l	27.7	1.22	/	2,940	14,100	33,800	12,600	74,500	8.1	1,220	63.3	125	505	29,600	11.1	41.3	77.4	1,210	9.7	6.5	0.65	146	19.2	/	5.25
NWHR-1	1.00 g/l	26.6	1.2	/	2,010	13,600	33,300	10,300	62,400	8.1	1,200	62.2	122	487	28,900	10.7	37.6	75.5	1,150	9.43	6.45	0.696	134	18.8	/	5.14
NWWQB-1	1.00 g/l	55.4	2.25	/	2,870	14,100	68,900	24,300	25,600	14	1,090	116	83.3	2,410	50,100	18.7	65.7	82.5	387	19	25.1	1.32	99.3	23.7	1.62	0.851
NWWQB-1	1.00 g/l	54.3	2.31	42	2,420	14,600	70,000	23,700	/	14	1,100	118	85.2	2,460	52,000	19.1	67.8	83.4	/	19.5	25.9	1.37	94.8	24.1	/	0.872
NWWQB-1	1.00 g/l	47.5	2.12	/	2,160	13,200	62,500	20,900	/	13	974	105	75.3	2,230	47,100	17.4	61	75.6	331	17.2	23.6	1.27	84.6	21.7	1.59	0.793
BCR 667	1.00 g/l	40.3	1.61	36	12,000	18,300	42,300	12,300	37,700	8.5	715	72.7	124	730	35,700	17.5	100	49.7	/	11.2	15.1	0.496	157	12.7	/	0.271
BCR 667	1.00 g/l	42.4	1.71	39	12,000	17,800	46,700	13,500	35,800	9	821	76.6	127	705	35,300	17	97.1	47.7	/	11.9	14.6	0.474	166	12.9	/	0.272
BCR 667	1.00 g/l	44.4	1.82	100	13,200	18,600	48,800	14,100	38,200	8.7	826	83.7	142	733	36,200	18.2	102	52.6	/	12.6	17.8	7.71*	209	13	21.4*	0.313
CRM-MS-S	0.10 g/l	62.3	1.8	/	18,300	11,300	60,200	/	/	10	2,140	98.6	79.5	439	38,800	9.67	26.9	33.5	/	14	18.4	/	179	18	/	/
CRM-MS-S	0.10 g/l	58.3	1.51	/	18,000	10,300	57,500	/	/	9.6	2,100	93.1	70.6	407	36,900	8.81	25.3	33.1	/	12.7	17.7	/	171	16.3	/	/
CRM-MS-S	0.10 g/l	64.8	1.41	/	17,700	10,200	55,800	/	/	9.5	2,210	94.9	76	418	36,200	9.03	28	36.9	/	13.1	17.2	/	163	15.7	/	/
Metranal-18	0.10 g/l	/	2.75	/	/	4,940	39,000	/	/	8.5	1,510	97.4	67.7	449	31,200	12.6	40.6	109	/	12.5	38.3	/	206	16.5	/	5.11
Metranal-18	0.10 g/l	/	3.1	/	/	5,270	40,000	/	/	8.9	1,500	101	84.1	468	32,800	13.7	46	109	/	12.6	37.9	/	209	16.7	/	3.95
Metranal-18	0.10 g/l	/	2.78	/	/	5,500	44,000	/	/	9.4	1,690	105	75.2	482	34,100	14.8	45.8	122	/	12.7	39	/	216	17.9	/	4.58
Metranal-1	0.10 g/l	/	1.59	/	/	3,940	22,700	/	/	4.7	985	51.3	72.4	939	20,800	9.82	30	63.2	/	6.48	22.5	/	106	11.2	/	1.95
Metranal-1	0.10 g/l	/	1.68	/	/	3,950	21,700	/	/	4.3	929	48.9	70	952	20,000	9.58	28.6	60.6	/	6.4	21.4	/	105	10.7	/	1.81
Metranal-1	0.10 g/l	/	1.7	/	/	4,170	23,900	/	/	4.8	1,110	50.9	71.7	942	21,200	10	28.2	58.4	/	6.93	21.5	/	105	11.4	/	1.81
NWHR-1	0.10 g/l	/	1.45	/	/	14,500	43,600	13,500	/	9.3	1,520	78	134	551	32,400	11.8	43.8	85.4	/	11.9	8.34	/	163	21.9	/	5.72
NWHR-1	0.10 g/l	/	1.38	/	/	14,200	38,800	12,300	/	8.7	1,390	74.7	130	512	31,300	11.1	41.9	82.6	/	10.6	6.89	/	153	20.1	/	5.41
NWHR-1	0.10 g/l	/	1.27	/	/	14,300	36,500	11,600	/	8.4	1,370	72.6	128	523	30,600	11	47.8	84.1	/	10.2	7.02	/	157	19.7	/	6.36
NWWQB-1	0.10 g/l	42.3	1.61	/	/	9,720	50,500	17,200	/	9.4	790	85.1	59.8	1,580	34,800	12.8	45.5	56.2	/	13.3	17.3	/	71.2	16.8	/	0.625
NWWQB-1	0.10 g/l	37.9	1.61	/	/	9,260	46,300	15,900	/	8.9	715	80.3	55.9	1,550	33,300	13	49.1	86.4	/	12.7	17.2	/	63.7	16	/	0.593
NWWQB-1	0.10 g/l	41.6	1.67	/	/	9,430	50,500	17,400	/	9.2	865	84.4	60.1	1,580	33,900	12.4	46.1	54.5	/	13.6	17.2	/	70.1	16.6	/	/
BCR 667	0.10 g/l	51.4	2.48	/	18,300	23,000	64,700	19,700	/	12	1,190	112	168	959	47,100	23.2	134	63	/	16.6	19.4	/	214	18.1	/	/
BCR 667	0.10 g/l	56.4	2.45	/	18,100	23,800	66,800	19,800	/	13	1,220	116	176	985	49,800	24.4	137	65.5	/	17.3	21.5	/	227	18.8	/	/
BCR 667	0.10 g/l	62	2.29	/	17,100	22,400	61,500	18,500	/	12	1,190	108	172	940	47,500	22.8	131	64.9	/	15.8	19.9	/	213	17.3	/	/
CRM-MS-S	0.25 g/l	27.3		/	15,500	8,830	43,800	4,510	30,100	8.3	1,290	69.5	65.1	359	32,300	7.95	19.5	38.4	190	10.4	15.7	/	152	13.6	1.62	/
CRM-MS-S	0.25 g/l	24.5	1.17	/	15,500	8,610	45,800	4,690	27,400	8.9	1,570	67.4	61.3	340	30,700	7.82	19.9	35.8	151	10.6	15.2	/	141	12.9	1.95	/
Metranal-18	0.25 g/l	/	2.23	/	/	4,090	32,000	7,280	46,000	8	1,240	70.6	61.8	375	26,300	11.4	36.5	117	1,050	9.57	31.7	/	166	12.2	1.8	5.33
Metranal-18	0.25 g/l	/	2.19	/	953	3,870	35,000	7,660	41,400	8.1	1,410	65.7	55.8	346	24,300	11.4	34.4	113	1,010	9.95	28.7	/	163	12.7	2.31	3.17
Metranal-1	0.25 g/l	/	1.72	/	/	4,590	23,500	6,220	/	5.7	939	/	/	1,160	24,500	12.7	38	106	641	6.84	/	/	111	12.6	1.16	3.02
Metranal-1	0.25 g/l	/	1.94	/	873	4,740	28,100	7,350	18,500	6.3	1,170	49.2	89.9	1,160	24,600	12.4	38.1	110	581	7.77	25.1	/	124	13.4	1.25	2.74

Table S 6 continued.

NWHR-1	0.25 g/l	/	1.16	/	1,610	12,300	32,500	9,950	53,300	8.3	1,110	56	120	452	27,400	10.3	36.5	92.3	1,330	9.21	/	/	132	16.6	1.52	5.77
NWHR-1	0.25 g/l	/	/	/	2,340	12,300	36,000	10,700	52,900	8.7	1,230	58.8	121	467	28,000	10.7	37.4	98.4	1,230	9.8	6.36	/	139	17.1	/	6.1
NWWQB-1	0.25 g/l	25.2	2.05	81	1,950	12,000	61,200	20,500	9,920	13	952	95.6	75.5	2,140	46,100	17.8	64.5	96.9	448	16.4	23.2	/	78.6	19.7	1.42	0.888
NWWQB-1	0.25 g/l	/	2.28	88	2,720	12,400	73,500	23,600	9,990	15	1,130	112	85.7	2,170	47,100	18.3	66.5	93.3	346	19.4	24.6	/	97.9	21.3	/	0.895
BCR 667	0.25 g/l	/	/	85	12,900	18,700	50,100	14,400	39,400	11	844	82.8	149	833	41,700	21.3	131	77.1	234	13	17.5	/	174	13.8	1.51	0.355
BCR 667	0.25 g/l	/	2.04	89	13,300	19,500	55,200	16,000	39,500	12	967	86.7	157	845	42,600	21.6	135	74.7	278	14.3	17.9	/	183	14.6	1.36	0.39

**CRM**	**SPM_conc**	**Cd**	**In**	**Sb**	**Te**	**Cs**	**La**	**Ce**	**Pr**	**Nd**	**Sm**	**Eu**	**Gd**	**Tb**	**Dy**	**Ho**	**Er**	**Tm**	**Yb**	**Lu**	**Hg**	**Tl**	**Pb**	**Th**	**U**
CRM-MS-S	1.00 g/l	0.33	0.06	1.2	1.16	3.6	36.8	77.4	8.55	34	6.19	1.31	5.08	0.69	3.89	0.713	1.9	0.252	1.6	0.221	0.079	0.487	75.7	9.87	3.82
CRM-MS-S	1.00 g/l	0.32	/	1.9	0.928	3.37	31.3	66.4	7.36	29	5.62	1.18	4.46	0.62	3.5	0.66	1.76	0.233	1.45	0.211	0.051	0.445	99.3	8.71	3.55
CRM-MS-S	1.00 g/l	0.3	0.08	1.3	1.03	3.27	32.8	69.5	7.69	31	5.78	1.15	4.69	0.65	3.59	0.651	1.72	0.229	1.48	0.197	0.052	0.438	69.3	9.15	3.94
Metranal-18	1.00 g/l	2.64	0.05	9.8	4.95	6.29	27	58.1	6.32	25	4.92	0.94	3.84	0.51	2.85	0.508	1.35	0.172	1.08	0.165	0.788	1.54	202	8.67	2.76
Metranal-18	1.00 g/l	3.34	0.08	16	6.23	7.58	34.2	73.2	7.94	32	6.16	1.2	4.96	0.66	3.69	0.653	1.68	0.218	1.37	0.193	1.15	1.84	243	10.8	3.46
Metranal-18	1.00 g/l	4.06	0.08	69	7.08	9.24	42.3	89.4	9.81	39	7.52	1.47	6.03	0.82	4.36	0.783	2.04	0.259	1.65	0.225	0.917	2.21	394	13.3	4.19
Metranal-1	1.00 g/l	2.9	0.05	2.4	3.82	5.79	31.2	63.5	7.17	28	5.5	0.89	4.43	0.58	3.13	0.546	1.35	0.174	1.1	0.151	1.42	0.473	87.7	9.36	2.59
Metranal-1	1.00 g/l	2.99	0.06	2.3	4.02	5.97	38.3	78.1	8.64	34	6.28	1.02	5.15	0.64	3.52	0.613	1.54	0.199	1.22	0.163	1.34	0.521	91.4	10.8	3.08
Metranal-1	1.00 g/l	3	/	2.4	4.15	5.86	37.1	75.3	8.38	33	6.39	0.94	5.05	0.66	3.43	0.623	1.54	0.194	1.19	0.158	1.31	0.506	93	10.5	3
NWHR-1	1.00 g/l	4.02	/	1.7	2.73	2.73	24.7	54	6.38	27	5.44	1.04	4.6	0.64	3.78	0.711	1.89	0.255	1.68	0.234	0.251	0.42	144	6.07	0.976
NWHR-1	1.00 g/l	4	0.05	1.7	2.69	2.77	26.2	56.6	6.62	28	5.43	1.06	4.5	0.64	3.69	0.714	1.87	0.257	1.61	0.225	0.258	0.419	145	6	1
NWHR-1	1.00 g/l	3.78	/	1.6	2.61	2.72	26.8	57.4	6.87	28	5.6	1.05	4.56	0.63	3.67	0.68	1.83	0.247	1.57	0.223	0.239	0.404	142	5.52	0.936
NWWQB-1	1.00 g/l	1.9	0.1	1.5	5.8	7.04	37.8	80.1	9.44	38	7.59	1.46	6.21	0.85	4.82	0.877	2.29	0.3	1.94	0.271	1.1	1.35	92.3	9.49	2.83
NWWQB-1	1.00 g/l	2.01	0.1	1.5	5.98	7.37	38.5	81.7	9.65	39	7.99	1.54	6.33	0.88	4.84	0.894	2.31	0.313	1.92	0.272	1.15	1.38	95.8	9.79	2.63
NWWQB-1	1.00 g/l	1.83	0.08	1.3	5.33	6.63	33.7	71.7	8.53	35	7.05	1.38	5.81	0.78	4.38	0.825	2.13	0.277	1.82	0.242	1.04	1.25	87.8	8.77	2.81
BCR 667	1.00 g/l	0.5	/	0.6	1.8	5.88	15.4	31.9	3.72	15	3.55	0.72	3.16	0.44	2.53	0.459	1.2	0.148	0.941	0.133	0.034	0.488	27.2	5.93	0.9
BCR 667	1.00 g/l	0.49	/	0.6	2.06	6.08	17.3	35.4	4.06	17	3.59	0.73	3.2	0.44	2.54	0.46	1.23	0.153	0.958	0.133	0.032	0.515	26.6	6.09	0.926
BCR 667	1.00 g/l	0.51	0.06	2.9	2.82	6.25	18.2	37.1	4.19	17	3.75	0.78	3.28	0.47	2.68	0.482	1.26	0.165	0.991	0.14	0.033	0.567	28.1	6.01	1.96
CRM-MS-S	0.10 g/l	/	/	/	1.47	3.64	34.2	72.3	8.1	32	5.62	1.19	4.8	0.65	3.62	0.683	1.89	0.253	1.59	0.208	/	0.461	72.6	8.32	3.48
CRM-MS-S	0.10 g/l	/	/	1.2	1.47	3.29	30	62.9	6.8	27	5.07	1.02	4.28	0.6	3.46	0.63	1.71	0.247	1.62	0.208	/	0.422	71.3	7.47	3.3
CRM-MS-S	0.10 g/l	/	/	1.1	1.31	3.18	28.3	58.5	6.53	25	4.97	1.04	4.04	0.6	3.28	0.603	1.49	0.212	1.36	0.215	/	0.418	61.4	7.25	3.19
Metranal-18	0.10 g/l	2.94	/	14	5.4	6.99	30.9	64.7	7.15	28	6.02	1.12	4.24	0.59	3.45	0.608	1.33	0.21	1.2	0.157	0.249	1.63	205	8.92	3.3
Metranal-18	0.10 g/l	3.21	/	8	5.93	7.01	32.6	69.1	7.6	30	5.49	1.1	4.62	0.64	3.2	0.66	1.65	0.184	1.27	0.192	/	1.7	214	9.49	3.32
Metranal-18	0.10 g/l	3.04	/	9.4	5.87	7.7	33.8	70.8	7.56	30	6.29	1.18	4.72	0.64	3.67	0.653	1.7	0.257	1.32	0.146	/	1.78	206	9.05	3.17
Metranal-1	0.10 g/l	2.52	/	2.1	2.98	4.57	20.4	41.6	4.66	18	3.66	0.67	2.86	0.43	2.29	0.403	0.987	0.144	0.792	0.12	0.347	0.375	66	5.61	1.87
Metranal-1	0.10 g/l	2.47	/	1.7	2.78	4.42	17.5	34.5	3.86	15	3.04	0.53	2.58	0.38	1.97	0.388	0.964	0.133	0.743	0.12	0.728	0.37	61.8	3.89	1.61
Metranal-1	0.10 g/l	2.36	/	1.6	3.06	4.19	19.6	40.2	4.6	18	3.47	0.65	3.04	0.39	2.34	0.401	1.06	0.151	1	0.12	0.094	0.399	65.3	5.45	1.7
NWHR-1	0.10 g/l	4.34	/	1.8	3.33	3.11	29.5	64.1	7.58	31	5.67	1.23	4.86	0.75	4.37	0.823	2.11	0.276	2.02	0.257	/	0.518	154	6.32	/
NWHR-1	0.10 g/l	4.57	/	1.7	3.1	2.97	25.2	54.7	6.58	27	5.64	1.19	4.45	0.69	3.87	0.774	2.03	0.277	1.68	0.3	/	0.502	152	5.98	/
NWHR-1	0.10 g/l	4.15	/	1.7	2.98	2.63	24.8	53.2	6.3	26	5.92	1.01	4.75	0.67	4.1	0.756	2.04	0.268	1.81	0.219	/	0.442	152	5.67	/
NWWQB-1	0.10 g/l	1.27	/	1.1	4.28	4.89	25.2	54	6.31	26	5.08	1.04	4.1	0.61	3.49	0.641	1.59	0.24	1.43	0.204	0.273	0.95	63.3	6.43	1.88
NWWQB-1	0.10 g/l	1.31	/	1	4.22	4.73	23.3	50.1	6.05	24	5.02	0.96	4.05	0.58	3.38	0.643	1.61	0.206	1.35	0.177	0.425	0.906	60.5	6.31	1.81
NWWQB-1	0.10 g/l	1.36	/	1.1	4.22	4.87	26.3	56.2	6.69	27	5.6	1.05	4.39	0.56	3.43	0.61	1.65	0.223	1.4	0.17	0.497	0.958	62.5	6.38	1.9
BCR 667	0.10 g/l	0.78	/	/	3.14	8.09	24.3	49.5	5.6	22	4.79	0.96	4.24	0.63	3.4	0.622	1.61	0.209	1.35	0.175	/	0.723	35.4	7.91	/
BCR 667	0.10 g/l	0.97	/	/	3.32	8.5	23.8	50.1	5.43	24	4.93	1.01	4.66	0.66	3.72	0.687	1.69	0.223	1.39	0.176	/	0.722	37.6	8	/
BCR 667	0.10 g/l	0.67	/	/	3.14	8.01	22.5	45.7	5.12	21	4.43	1	4.17	0.62	3.2	0.594	1.6	0.248	1.39	0.147	/	0.699	35.2	7.3	/
CRM-MS-S	0.25 g/l	/	/	/	/	3.09	27.7	60.2	6.82	28	5.39	1.08	4.54	0.59	3.49	0.603	1.7	0.215	1.37	0.188	/	2.62	70.5	7.36	3.58

Table S 6 continued.

CRM-MS-S	0.25 g/l	/	/	/	/	3.04	27.1	56.9	6.62	25	4.94	1.02	4.08	0.53	3.25	0.553	1.57	0.199	1.24	0.174	/	2.61	65	7.39	3.19
Metranal-18	0.25 g/l	2.85	/	13	/	6.53	26.3	56.8	6.35	26	5.11	1.01	4.33	0.55	3.19	0.539	1.48	/	1.18	0.167	/	1.6	213	8.01	2.9
Metranal-18	0.25 g/l	2.47	/	/	/	6.22	27	57.2	6.53	25	4.77	0.91	4.01	/	/	0.531	1.44	0.176	1.17	0.157	/	1.57	202	8.28	3
Metranal-1	0.25 g/l	3.55	/	/	/	6.06	29.3	59.9	6.96	/	/	0.95	/	/	/	/	/	0.162	1.05	/	/	0.517	99.3	7.44	2.79
Metranal-1	0.25 g/l	3.42	/	/	/	6.02	25.5	52.3	6.12	23	4.57	0.87	4.02	0.52	3.06	0.523	1.44	0.189	/	0.151	/	0.608	99.2	6.73	2.61
NWHR-1	0.25 g/l	4.18	/	/	/	2.86	24.4	53.5	6.74	28	5.74	1.05	4.88	0.65	4.03	0.715	2.02	0.26	1.53	/	/	0.49	163	5.99	1.01
NWHR-1	0.25 g/l	4.07	/	/	/	3	24.8	55	6.67	28	5.59	1.03	4.8	0.65	4.03	0.745	2.13	0.259	1.74	0.254	/	0.482	162	6.11	1.09
NWWQB-1	0.25 g/l	2.11	/	/	/	6.99	32.3	69.7	8.55	35	7.37	1.43	6.31	0.82	4.82	0.835	2.3	0.304	1.83	0.261	/	1.43	97.7	8.98	31.2*
NWWQB-1	0.25 g/l	1.7	0.08	/	/	7.37	38.6	82.3	9.98	41	8.35	1.51	6.85	0.92	5.35	0.95	2.59	0.359	2.05	0.299	/	1.58	101	9.73	2.96
BCR 667	0.25 g/l	/	/	/	/	7.54	19	39.3	4.74	20	4.56	0.87	4.21	0.57	3.39	0.57	1.62	0.186	1.27	0.164	/	0.673	38.5	7.48	1.21
BCR 667	0.25 g/l	/	/	/	/	7.93	20.9	43.5	5.26	21	4.62	0.88	4.29	0.58	3.46	0.602	1.62	0.199	1.28	0.17	/	0.733	38.1	7.52	1.26

7. Concentrating the whole water sample – XpressVap

The XpressVap (CEM, Germany) was used to concentrate the whole water samples prior to digestion. Therefore, we firstly needed to find the optimal duration of the concentration step (Table S 5). A maximum of 24 vessels can be concentrated in one run. Figure S 4 shows the recovery for all elements analysed after concentration and subsequent digestion with the final digestion protocol ($2HNO_3$:1HCl). Se or Hg are known to be volatile elements (Sastre et al., 2002) and lower recoveries were obtained for these elements leading to the conclusion that concentration of the sample may cause an uncertainty (randomly occurring based on the respective unknown fractionation of the elements) for potentially volatile analytes (Figure S 4). In addition, the finale volume within the same run differed significantly delivering another uncertainty factor. In some cases, recoveries > 200% were achieved: Ti, Sb, Pb in CRM-MS-S; Ba, in Metranal-18; Hg in NWHR-1; Na in NWWQB-1; Rb, Sr, Zr, Nb, Ba, W, in SdAR-M2 showing a higher digestion efficiency as explained in the manuscript.

Table S 7: Microwave XpressVap programs for different number of vessels leading to a final volume of 0.3 to 3 ml per vessel.

No. of vessels	Microwave XpressVap program		final volume / ml
	step 1	step 2	
12	800 W, 20 min		0.7 – 3
20	1200 W, 20 min	600 W, 10 min	0.3 – 1.2
24	1400 W, 20 min	600 W, 10 min	0.8 – 1.9

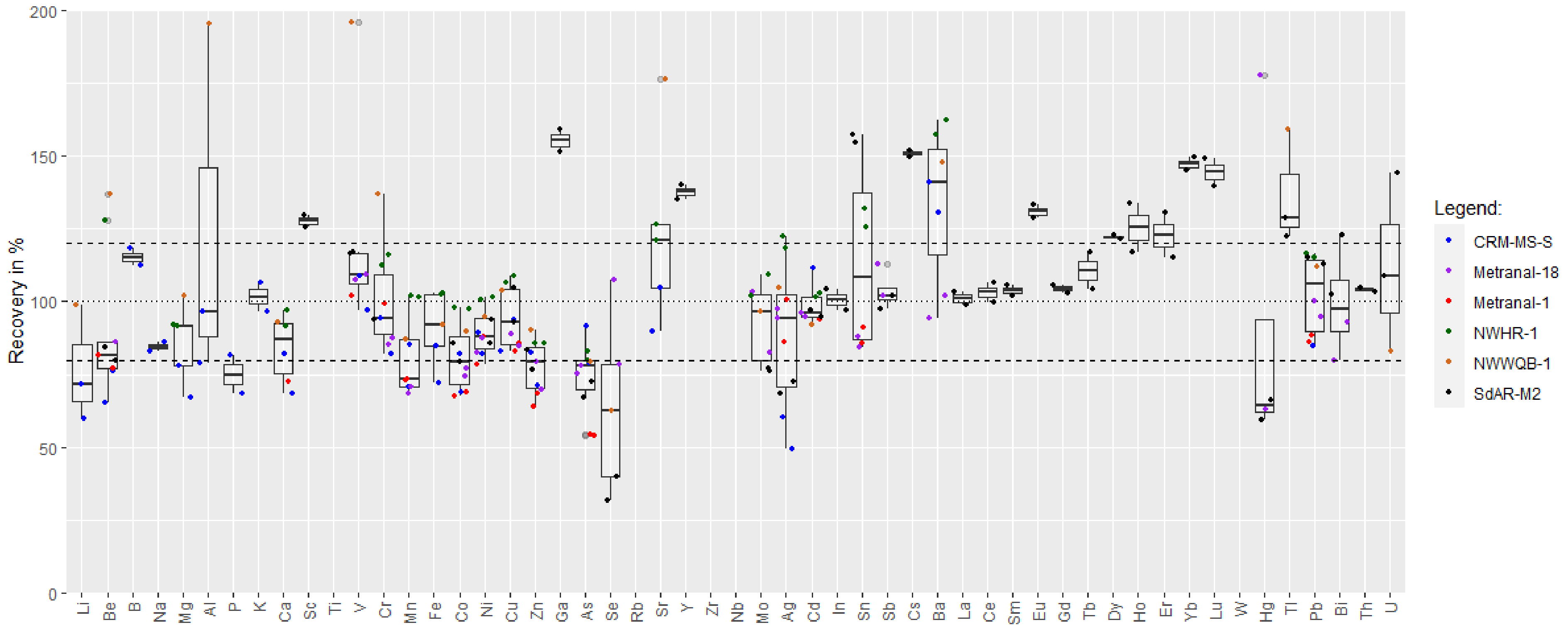

Figure S 4: Recoveries in % of all elements included in the optimised multi-element method using the XpressVap (CRMs: CRM-MS-S, Metranal-18, Metranal-1, NWHR-1, NWWQB-1 and SdAR-M2) with a digestion using 8 ml HNO_3 and 4 ml HCl at 150 °C and 20 min hold time. The boxplots indicate the range of the recoveries per element for all CRMs while the points indicate the recovery per CRM as explained by the legend. The calculation of the recoveries is based on the aqua regia extractable values.

8. Additional data of section 3 of the manuscript

8.1 Matrix correction prior to ICP-QQQ-MS analysis

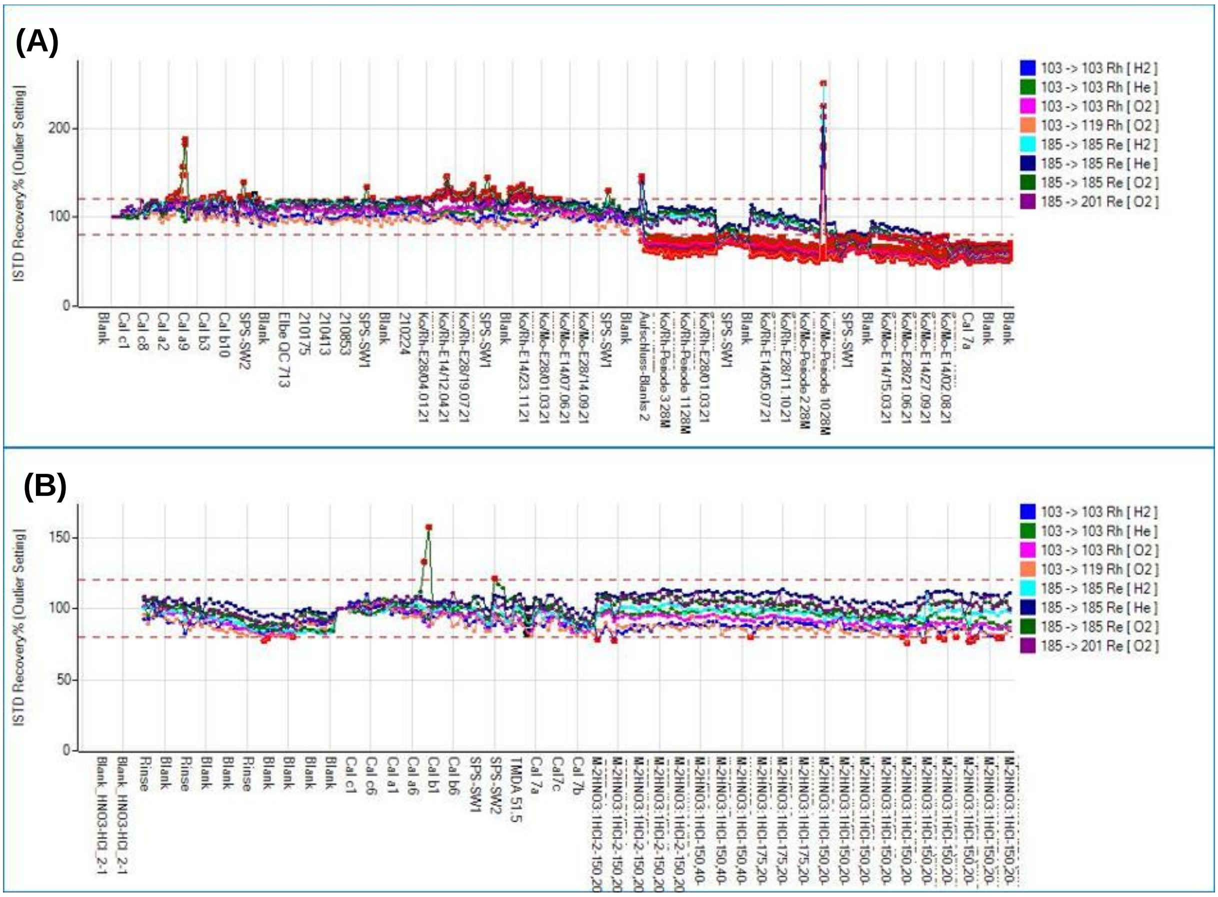

Figure S 5: Internal standard variation during two different ICP-QQQ-MS analytical runs using (A) no matrix matching and (B) matrix matching which leads to a stable internal standard throughout the analysis run. Run (A) was conducted with standards in a 1.3 % HNO_3 matrix while the digested samples consisted of a diluted aqua regia matrix. Run (B) shows the optimised digestion protocol using matrix matched solutions by using diluted digestion matrix solution of the optimised digestion protocol (diluted $2HNO_3$:1HCl matrix, 1:50 diluted).

8.2 Open vs. closed-vessel

Table S 8A: Recoveries in % related to the referenced aqua regia extractable values in two CRMs NWWQB-1 and SdAR-M2 testing different microwave digestion devices/approaches: Beaker and DEENA which are two open-vessel approaches and the microwave which is a closed-vessel approach. Aqua regia was applied for the beaker approach and the optimised digestion reagent for the DEENA and the microwave. Recoveries > 120% are marked in italic font. Acceptable recoveries of between 80 and 120% are marked in bold font. Please note that recoveries >100% relative to the aqua regia extractable values may indicate a higher digestion efficiency, as long as the values are still below 100% of the total element contents.

	**Beaker (n=6)**												**DEENA (n=4)**							
**CRM**	**NWWQB-1**						**SdAR-M2**						**NWWQB-1**				**SdAR-M2**			
**Li**	75	30	**82**	*137*		**92**			**96**	*130*	**90**	**105**	37	31	50	70	24	29	6	61
**Be**	*137*		*162*	*193*	45	*173*	**91**	44	54	*126*	**89**	**112**	55	63	**105**	*125*	45	63	30	**91**
**B**							*763*	*214*	*277*	*3248*	*788*	*1307*					17	29	6	**100**
**Na**	*1043*	*773*	*899*	*1116*		*1138*							*153*	**110**	*161*	*194*				
**Mg**	*123*	**102**	**119**	*130*		*126*							51	44	65	75				
**Al**	**119**	52	*152*	*205*	31	*123*							70	63	**92**	**111**				
**Ca**	**113**	**108**	**115**	**119**	**113**	*122*							*148*	45	59	63				
**Sc**							*129*	65	**102**	*150*	**117**	*132*					51	64	34	**81**
**V**	63	28	*141*	*226*		70	62	20	22	*143*	48	*121*	69	62	**96**	**114**	43	53	30	**93**
**Cr**	71	46	*134*	*172*		65	*124*		56	*167*	**114**	*178*	58	49	72	**86**	45	57	35	**86**
**Mn**	33	14	77	**107**		51							40	37	56	64				
**Fe**	42	11	**88**	**115**		42							44	40	60	69				
**Co**	50	19	**87**	**112**		**85**	56	12	58	**101**	56	**98**	44	39	58	71	43	53	30	**84**
**Ni**	**103**	45	*156*	**120**		**86**	*124*	68	**112**	**111**	**86**	**112**	50	46	68	78	51	64	35	**102**
**Cu**	**97**	71	*129*	*244*		*255*	**89**	62	**89**	*149*	*132*	*150*	51	46	64	**81**	50	62	34	**102**
**Zn**	**95**	67	**111**	*131*		*134*	**91**	**83**	**93**	**105**	**107**	**116**	66	50	62	**83**	53	60	44	**103**
**Ga**							**106**	29	46	*220*	**86**	*158*					**94**	**112**	68	*182*
**As**	35	22	**83**	**118**	11	46	26	17	20	**94**	32	**99**	45	40	59	77	40	49	32	**84**
**Se**			66							**95**	15	56	30	21	55	57	27	23	17	66
**Rb**							*268*	*185*	*232*	*366*	*220*	*243*					**105**	*124*	71	*213*
**Sr**	*172*	*147*	*177*	*215*		*176*	*209*	*160*	*198*	*272*	*202*	*204*	77	60	**86**	**97**	**99**	**118**	66	*175*
**Y**							**117**	**88**	**112**	*121*	**108**	*123*					51	62	37	**103**
**Zr**										*516*							41	24	13	19
**Nb**							19			*329*		39					**117**	*132*	72	*213*
**Mo**	47		**84**	*141*		41	24	8	12	**88**	24	**98**	27		22	19	39	47	24	**84**
**Ag**	**85**	48	79	*3495*		*907*	34	66	61	*162*	**103**	48	39	36	52	70	41	42	31	74

	**Microwave (n=10)**																	
**CRM**	**NWWQB-1**									**SdAR-M2**								
**Li**	*134*	*131*																
**Be**	*196*	*211*	*225*			*163*				*127*		*133*		**112**			*123*	
**B**																		
**Na**					*1002*	*1063*	*1239*		*1285*									
**Mg**	*142*	*133*	*132*	*137*	*136*	*135*	*151*	*144*	*144*									
**Al**	*293*	*257*	*290*	*271*	*283*	*281*	*298*	*312*	*303*									
**Ca**	*121*	**114**	*161*	*163*	*170*	*159*		*169*	*165*									
**Sc**										*152*	*146*	*173*				*154*		*144*
**V**	*260*	*240*	*245*	*246*	*244*	*251*	*248*	*244*	*247*	*129*	*129*	*147*	*141*	*127*		*134*	*133*	*136*
**Cr**	*180*	*172*		*170*	*184*	*177*		*171*										
**Mn**	**104**	**99**	**97**	**99**	**100**	**101**	**100**	**97**	**101**									
**Fe**	**117**	**108**	**110**	**109**	**111**	**112**	**112**	**109**	**111**									
**Co**	**109**	**107**	**109**	**105**	**114**	**114**	**114**	**112**	**112**	**98**	**104**	**100**	**87**	**97**	**98**	**95**	**87**	**98**
**Ni**			**117**	**109**	**118**	**108**	**110**	**101**	**105**				**92**	**103**	**105**	**96**	**100**	**97**
**Cu**	*124*	*120*	**106**	**103**	**108**	**108**	**109**	**103**	**104**	**110**	**110**	**113**	**92**	**101**	**94**	**98**	**94**	**95**
**Zn**	*126*	*121*	**107**	**115**	**108**	**117**	*149*	*128*	**114**	**106**	**101**	**99**	**92**	**89**	**97**	**93**	**96**	**93**
**Ga**										*214*	*219*	*259*	*227*	*218*	*221*	*216*	*216*	*219*
**As**	**112**	**108**	**112**	**114**	*122*	**113**		**105**	*126*	**90**	**86**	**91**					**95**	**91**
**Se**																		
**Rb**										*355*	*358*	*424*	*389*	*332*		*340*	*362*	*354*
**Sr**	*258*	*238*	*245*	*247*	*263*	*255*		*246*	*286*	*264*	*282*	*339*					*323*	
**Y**										*131*	*126*	*145*	*139*	*126*		*132*	*152*	*139*
**Zr**																		
**Nb**										*205*								
**Mo**			*121*						**108**	**86**	75	**87**	78	**82**	78	**86**	**88**	**90**
**Ag**	**115**	**108**	**111**	**101**	**108**	**113**	**113**	**98**	**104**	58	56	59	*260*	60	64	55	66	80

Table S8 continued.

**Cd**	**101**	80	**107**	**111**		*121*	**97**	**94**	**100**	**105**	**100**	**104**	38	42	56	**80**	46	65	34	**96**	**94**	**102**				**118**				**99**	**86**	**99**				**84**	**88**	
**In**							**81**	51	55	**98**	77	**101**					48	61	35	**97**										**97**	**102**	**98**	**92**	**99**	**100**	**110**	**100**	**99**
**Sn**							*560*	**95**	*1710*	*695*	*1632*	*530*					24	31	41	**113**																		
**Sb**							10	7	8	**114**	5	11					47	59	33	**96**										**106**	**103**	**106**	**96**	**104**	**99**	**105**	**101**	**105**
**Te**																	71	78	33	*148*																		
**Cs**							*218*	*136*	*191*	*205*	*172*	*191*					63	77	51	*139*												*172*			*165*	*160*		
**Ba**	*142*	*123*	*138*	*159*		*144*	*194*	*157*	*184*	*272*	*173*	*181*	62	55	**83**	**96**	**100**	**118**	66	*198*	*186*	*165*	*175*	*172*	*181*	*186*	*192*	*182*	*189*	*270*	*289*	*359*	*310*	*280*	*294*	*296*	*298*	*307*
**La**							**99**	**90**	**103**	**100**	**101**	**103**					47	56	33	**83**										**105**	**93**	**97**	**95**	**96**	**96**	**99**	**108**	**111**
**Ce**							71	24	65	**103**	76	**105**					50	60	35	**90**										**103**	**92**	**96**	**93**	**95**	**95**	**98**	**104**	**108**
**Sm**							**105**	77	**109**		**105**	**112**					52	60	35	**95**										**115**	**105**	**109**					*121*	**119**
**Eu**							**117**	**80**	**115**		**114**	*125*					58	68	42	*122*										*137*	*126*	*140*					*136*	*139*
**Gd**							**104**	73	**104**	**114**	**102**	**110**					51	59	36	**94**										**114**	**107**	**117**					*123*	*123*
**Tb**							**105**	73	**104**		**100**	**111**					53	61	34	**97**												*122*					*130*	**117**
**Dy**							**106**	73	**104**		**100**	**112**					53	61	36	**103**										*122*	**116**	*126*					*139*	
**Ho**							**110**	75	**108**	**117**	**103**	**116**					54	65	35	**105**										*124*		*141*				*127*	*157*	*146*
**Er**							**107**	72	**106**		**100**	**114**					48	59	33	**103**										*131*	*124*	*139*				**120**	*146*	*135*
**Yb**							**119**	79	**110**		**107**	*122*					49	63	32	**111**										*131*	*131*	*152*	*128*		**113**	*140*	*150*	*143*
**Lu**							**111**	74	**110**		**99**	**116**					52	64	35	**109**										*136*		*155*		*151*				*162*
**Hf**																	42	70	29	50																		
**W**										*385*		**97**					*149*	*207*	*168*	*178*													*937*					
**Hg**	77		**86**	**100**						**97**		**81**	42	28	52	78	46	50	31	**96**																		
**Tl**	**88**	59	*123*	*158*		**114**	**81**	31	68	*128*	60	**108**	68	60	**82**	**101**	56	69	36	**104**	*178*	*172*	*171*		*170*	*185*	*168*			*128*		*128*	**118**	*125*	*128*	**119**	*136*	*128*
**Pb**	**94**	76	**113**	*121*		*145*	**95**	79	**96**	**105**	**101**	**105**	45	40	59	74	48	59	31	**96**	**109**	**104**	**109**	**113**	**110**	**115**	**109**	**113**	**109**	**103**	**101**	**103**	**98**	**103**	**103**	**103**	**104**	**106**
**Bi**							78	40	55	**116**	71	**112**					61	63	37	**99**										**108**	**102**	**107**	**104**	**99**	**98**	**106**	**97**	**93**
**Th**							**81**	36	54	**107**	**82**	**111**					44	58	32	58										**101**	**92**	**105**		**90**	**98**	**98**	**113**	**93**
**U**	**112**	68	*1486*	**82**		**92**	**103**	76	**102**	**117**	**96**	**105**	29	28	46	48	52	63	41	**95**	**93**	77		**93**		*137*		**106**	**102**	**113**			**95**	**91**	**98**	**103**	**114**	**98**

Table S8B: As an addition to table S8A, recoveries in % related to the referenced aqua regia extractable values for the DEENA and microwave approach using aqua regia are shown. Recoveries > 120% are marked in italic font. Acceptable recoveries of between 80 and 120% are marked in bold font. Please note that recoveries >100% relative to the aqua regia extractable values may indicate a higher digestion efficiency, as long as the values are still below 100% of the total element contents.

	DEENA (n=3)			Microwave (n=5)								
	NWWQB-1	NWWQB-1	NWWQB-1	NWWQB-1	NWWQB-1	NWWQB-1	NWWQB-1	SdAR-M2	SdAR-M2	SdAR-M2	SdAR-M2	SdAR-M2
**Be**	*153*	*188*	*282*	*158*	*193*	*187*	*207*	**103**		**107**	**105**	**112**
**Na**	*612*	*707*	*296*	*748*	*1069*	*663*	*922*					
**Mg**	**112**	*132*	34	**109**	*129*	*128*	*133*					
**Al**	*216*	*266*	*133*	*180*	*228*	*250*	*300*					
**Ca**	*123*	*129*	31	**110**	**114**	**105**	**106**					
**Sc**								*139*	*125*	*153*	*173*	*179*
**V**	*206*	*246*	**101**	*170*	*209*	*207*	*242*	**117**	**88**	**112**	**113**	*135*
**Cr**	*146*	*176*	55	*195*	*211*	*161*	*183*	**102**	*420*	*468*	**97**	*124*
**Mn**	**95**	**111**	37	**81**	**91**	**95**	**97**					
**Fe**	**105**	*126*	56	**81**	**92**	**107**	**109**					
**Co**	**103**	**119**	60	**83**	**92**	**108**	**111**	**96**	72	**88**	**92**	**94**
**Ni**	**114**	*136*	36	**117**	*126*	**112**	**115**	**107**	**112**	*134*	**102**	**103**
**Cu**	**120**	*141*	*622*	**95**	**104**	*131*	*126*	**111**	**87**	**95**	**114**	*129*
**Zn**	*158*	*174*	71	**95**	**104**	*174*	*134*	**107**	**93**	**94**	**113**	**110**
**Ga**								*238*	*157*	*229*	*204*	*248*
**As**	**112**	*128*	*1174*	**85**	**93**	**114**	*121*	**97**	67	75	**88**	**92**
**Se**	**103**	**116**	*150*	**81**	**100**			58		45	43	
**Rb**								*328*	*220*	*329*	*331*	*394*
**Sr**	*234*	*275*	**90**	*162*	*231*	*217*	*270*	*264*	*184*	*273*	*249*	*304*
**Y**								**115**	**80**	**101**	*125*	*134*
**Nb**								*300*	*216*			
**Mo**	**90**	**111**	**98**		*124*	**119**		**87**	62	74	**88**	**88**
**Ag**	*136*	*153*	*485*	**85**	**99**	**117**	**118**	75	45	66	62	69
**Cd**	**112**	*137*	59		**107**	*123*	**99**	**111**	**83**	**95**		**113**
**In**								**99**	75	**87**	**106**	**106**
**Sn**								*131*	**95**			
**Sb**								**114**	79	**94**	**116**	**119**
**Cs**								*154*	**106**	*130*	*177*	*193*
**Ba**	*159*	*191*	**103**	*128*	*162*	*198*	*223*	*289*	*194*	*300*	*327*	*390*
**La**								**102**	72	**85**	**114**	*124*
**Ce**								**101**	71	**86**	**115**	*126*
**Sm**								**113**	75	**89**	*126*	*145*

Table S8B continued.

**Eu**								*127*	**104**	*144*	*153*	*164*
**Gd**								**107**	74	**88**	*124*	*139*
**Tb**								**111**	**86**	**103**	*127*	*145*
**Dy**								**116**	**81**	**100**	*135*	*152*
**Ho**								**118**	**93**	**114**	*136*	*156*
**Er**								**117**	**92**	**116**	*132*	*149*
**Yb**								*128*	**99**	*135*	*135*	*156*
**Lu**								*134*	**100**	*128*	*141*	
**W**								*216*	*265*	*354*	*485*	*428*
**Hg**	**108**	**115**	27					**98**	64	74		
**Tl**	*157*	*204*	*256*	*127*	*154*	*194*	*215*	*129*	**91**	**108**	*140*	*144*
**Pb**	*129*	*152*	*141*	**84**	**99**	*124*	*128*	**112**	76	**90**	**117**	**119**
**Bi**								**115**	**83**	**95**	*125*	*139*
**Th**								**100**	80	**88**	*124*	*141*
**U**	**110**	**113**	24	63	**84**		**99**	**111**	78	**96**	*127*	*125*

8.3 Digestion reagent

Table S 9: Recoveries in % related to the referenced aqua regia extractable values of different HNO_3 and HCl ratios. HNO_3:HCl 6:2 means that 6 ml HNO_3 and 2 ml HCl were used for digestion at 150 °C and a hold time of 20 min. Recoveries > 120% are marked in italic font. Acceptable recoveries of between 80 and 120% are marked in bold font (n=7). Please note that recoveries >100% relative to the aqua regia extractable values may indicate a higher digestion efficiency, as long as the values are still below 100% of the total element contents.

Digestion	CRM	Li	B	Na	Mg	Al	K	Ca	Ti	V	Cr	Mn	Fe	Co	Ni	Cu	Zn	As	Se	Sr	Mo	Cd	Sn	Sb	Ba	Hg	Pb	Bi	U
HNO_3	CRM-MS-S	**87**	**99**	**97**	**101**	**85**	**102**	**105**	37	**92**	**95**	**98**	**93**	**87**	**92**	**109**	**98**	*128*		**103**					**92**		**118**		
HNO_3	CRM-MS-S	**86**	**92**	**99**	**100**	**81**	**100**	**106**	32	**88**	**87**	**97**	**92**	**85**	**87**	**94**	**98**	*127*		**102**					**92**		**110**		
HNO_3	Metranal-18							**95**		**112**	**103**	**105**		**96**	**109**	**108**	**108**	**100**				*149*	17		*586*		*132*		
HNO_3	Metranal-18							**92**		**102**	**95**	**101**		**90**	**101**	**98**	**106**	**95**				*159*	13		*537*		*155*		
HNO_3	Metranal-1									72	**81**	**88**		**90**	**92**	**89**	**95**	**103**	*158*		48	*123*	10	2	**93**		**109**	**99**	
HNO_3	Metranal-1									78	**85**	**90**		**91**	**94**	**87**	**96**	**104**	*162*		50	120	11	2	**95**		**109**	**97**	
HNO_3	NWHR-1				**103**			**105**			**106**	**104**	**96**	**97**	**103**	**100**	**103**	**104**		**97**	50	**119**	12		**103**		**117**		
HNO_3	NWHR-1				**107**			**107**			**108**	**107**	**99**	**99**	**105**	**101**	**103**	**103**		**99**	59	**117**	30		**107**		**118**		
HNO_3	NWWQB-1	**106**		**111**	**105**	**112**		**107**		**97**	**102**	**106**	**96**	**102**	**104**	**102**	**104**	**102**	**89**	**99**	26	*122*			**111**		121		**115**
HNO_3	NWWQB-1	**105**		**116**	**104**	**120**		**105**		**101**	**103**	**102**	**96**	**100**	**103**	**102**	**103**	**100**	**94**	**99**	34	*124*			**111**		**119**		**92**
HNO_3:HCl 6:2	CRM-MS-S	**98**	*178*	**108**	**115**	120	**109**	*141*	**111**	**120**	**116**	**111**	**100**	**102**	**104**	**100**	**98**	*131*		**113**		**94**					**84**		
HNO_3:HCl 6:2	CRM-MS-S	**109**	*191*	**115**	121	*131*	*124*	*148*	*136*	*130*	*131*	**117**	**111**	**111**	**116**	121	**108**	*146*		**120**		*228*					**108**		
HNO_3:HCl 6:2	Metranal-18							*128*		120	**112**	**108**		**101**	**110**	**105**	**103**	**96**				*130*					*138*		
HNO_3:HCl 6:2	Metranal-18							*133*		*132*	*124*	**114**		**105**	**117**	**105**	**107**	**117**				*150*					*134*		
HNO_3:HCl 6:2	Metranal-1									**108**	**95**	**93**		**100**	**104**	**92**	**92**	**110**	*171*		**107**	**108**		*123*		*129*	**105**		
HNO_3:HCl 6:2	Metranal-1									**119**	**103**	**102**		**110**	**114**	**103**	**100**	120	*184*		*126*	**115**		*145*		*148*	**117**		
HNO_3:HCl 6:2	NWHR-1				**117**			*145*			120	*123*	**109**	**113**	**115**	**107**	**105**	**107**		**114**	*127*	**108**				**116**	**114**		
HNO_3:HCl 6:2	NWHR-1				**117**			*147*			**120**	*122*	**109**	**114**	**115**	**108**	**107**	**111**		**114**	*137*	**109**				**114**	**117**		
HNO_3:HCl 6:2	NWWQB-1	*136*		*172*	*134*	*203*		**112**		*190*	*149*	**113**	**114**	**116**	**117**	**112**	**110**	*126*	**109**	*141*	*125*	**114**				**115**	**119**		**116**
HNO_3:HCl 6:2	NWWQB-1	*137*		*169*	*133*	*195*		**112**		*181*	*148*	**117**	**114**	**118**	**118**	**114**	**112**	*125*	**111**	*138*	*129*	**116**				**117**	121		**114**
HNO_3:HCl 9:3	CRM-MS-S	**91**	**114**	**104**	**113**	**99**	**110**	*131*	**85**	**100**	**104**	**110**	**104**	**93**	**92**	**102**	**112**	*143*		**113**					**107**		**96**		
HNO_3:HCl 9:3	CRM-MS-S	**97**	*130*	**106**	**118**	**106**	**118**	*135*	**104**	**106**	**108**	**111**	**107**	**97**	**98**	**117**	**108**	*146*		**116**					**118**		**100**		
HNO_3:HCl 9:3	Metranal-18							**111**		**105**	**107**	**103**		**90**	**102**	**95**	**104**	*163*				**107**	**115**		*504*		*131*		
HNO_3:HCl 9:3	Metranal-18							**116**		**99**	**101**	**106**		**92**	**102**	**101**	**110**	**105**				**114**	**113**		*520*		**115**		
HNO_3:HCl 9:3	Metranal-1									78	**82**	**85**		**87**	**93**	**84**	**90**	**108**	*155*		**87**	**95**	**113**	**98**	**83**	*141*	**93**	**90**	

Table S9 continued.

HNO_3:HCl 9:3	Metranal-1									**90**	**87**	**87**		**91**	**96**	**88**	**93**	**110**	*157*		**90**	**97**	**101**	**104**	**88**	*130*	**97**	**90**	
HNO_3:HCl 9:3	NWHR-1				**112**			*126*			**109**	**113**	**110**	**104**	**106**	**100**	**109**	**113**		**109**	**111**	**97**	*126*		**106**	**115**	**107**		
HNO_3:HCl 9:3	NWHR-1				**111**			*126*			**107**	**112**	**107**	**101**	**103**	**98**	**108**	**112**		**107**	*122*	**95**	*123*		**106**	*145*	**106**		
HNO_3:HCl 9:3	NWWQB-1	**113**		*126*	**117**	*154*		**103**		*129*	*122*	**103**	**103**	**101**	**104**	**103**	**105**	**117**	**100**	120	**113**	**99**			**107**	**104**	**105**		**113**
HNO_3:HCl 9:3	NWWQB-1	121		*128*	**118**	*156*		**103**		*132*	*129*	**109**	**108**	**107**	**109**	**107**	**110**	**118**	**99**	*121*	*124*	**99**			**107**	**104**	**105**		**98**
HNO_3:HCl 6:3	CRM-MS-S	**94**	*151*	**105**	**111**	**106**	**114**	**114**	**113**	**110**	**112**	**112**	**108**	**99**	**101**	**100**	**105**	*136*		**114**					*130*		**95**		
HNO_3:HCl 6:3	CRM-MS-S	**98**	*155*	**108**	**112**	**111**	**117**	**118**	*122*	**113**	**115**	**115**	**111**	**102**	**105**	**111**	**110**	*138*		**117**					*139*		**104**		
HNO_3:HCl 6:3	Metranal-18							**97**		**103**	**105**	**105**		**92**	**103**	**98**	**102**	**107**				**119**	*125*		*544*		*129*		
HNO_3:HCl 6:3	Metranal-18							**98**		**101**	**101**	**102**		**90**	**102**	**97**	**98**	**98**				**114**	*127*		*547*		*122*		
HNO_3:HCl 6:3	Metranal-1									**90**	**87**	**90**		**94**	**98**	**91**	**95**	**112**	*165*		**118**	**109**	*153*	**116**	**95**	*121*	**100**	**94**	
HNO_3:HCl 6:3	Metranal-1									**95**	**90**	**91**		**95**	**99**	**93**	**96**	**110**	*168*		**113**	**109**	*127*	**109**	**95**	**120**	**101**	**109**	
HNO_3:HCl 6:3	NWHR-1				**98**			**106**			**104**	**108**	**105**	**99**	**101**	**98**	**98**	**100**		**102**	**114**	**100**	*145*		**111**	**102**	**105**		
HNO_3:HCl 6:3	NWHR-1				**106**			**109**			**107**	**113**	**109**	**102**	**104**	**98**	**100**	**100**		**108**	**110**	**102**	*143*		**119**	**102**	**105**		
HNO_3:HCl 6:3	NWWQB-1	121		*145*	120	*172*		**95**		*153*	*132*	**107**	**109**	**107**	**108**	**107**	**109**	**117**	**105**	*132*	**109**	**109**			121	**104**	**108**		**84**
HNO_3:HCl 6:3	NWWQB-1	*129*		*159*	*126*	*190*		**97**		*169*	*143*	**109**	**113**	**110**	**111**	**109**	**112**	120	**106**	*141*	**115**	**111**			*124*	**105**	**110**		**96**
HNO_3:HCl 8:4	CRM-MS-S	**94**	*148*	**104**	**107**	**102**	**107**	**111**	**109**	**105**	**113**	**109**	**109**	**100**	**101**	**100**	**109**	*141*		**111**					*122*		**114**		
HNO_3:HCl 8:4	CRM-MS-S	**100**	*166*	**108**	**111**	**115**	**119**	**117**	*143*	**117**	*124*	**119**	**116**	**108**	**114**	*126*	**115**	*151*		**115**					*141*		**119**		
HNO_3:HCl 8:4	Metranal-18							**107**		*125*	*126*	**114**		**106**	**119**	**111**	**113**	*170*				*132*	*135*		*600*		*133*		
HNO_3:HCl 8:4	Metranal-18							**107**		**111**	**112**	**111**		**102**	**115**	**109**	**113**	*176*				*137*	*135*		*566*		*122*		
HNO_3:HCl 8:4	Metranal-1									**97**	**96**	**97**		**103**	**108**	**100**	**105**	*124*	*174*		*124*	121	**111**	*122*	**95**	*145*	**112**	**101**	
HNO_3:HCl 8:4	Metranal-1									**106**	**99**	**97**		**104**	**109**	**100**	**104**	*126*	*183*		**107**	121	*143*	120	**99**	*126*	**108**	**100**	
HNO_3:HCl 8:4	NWHR-1				**104**			**109**			**116**	**119**	**117**	**110**	**113**	**107**	**106**	**114**		**107**	**115**	**109**	*140*		**117**	**114**	**113**		
HNO_3:HCl 8:4	NWHR-1				**112**			**120**			**119**	*123*	**119**	**113**	**118**	**113**	**111**	*121*		**114**	*130*	**115**	*150*		**119**	**119**	120		
HNO_3:HCl 8:4	NWWQB-1	**114**		*123*	**107**	*138*		**94**		*123*	121	**108**	**107**	**108**	**111**	**110**	**111**	*124*	**102**	**113**	120	**112**			**106**	**107**	**112**		*141*
HNO_3:HCl 8:4	NWWQB-1	*121*		*135*	**115**	*153*		**98**		*136*	*127*	**108**	**110**	**110**	**112**	**111**	**112**	*128*	**107**	*121*	121	**117**			**112**	**112**	**114**		**112**
HNO_3:HCl 3:6	CRM-MS-S	**91**	*143*	**91**	**98**	**107**	**103**	**109**	*195*	**101**	**104**	**94**	**94**	**89**	**95**	**90**	**92**	*125*		**100**					*133*		**90**		
HNO_3:HCl 3:6	CRM-MS-S	**93**	*146*	**96**	**104**	**114**	**112**	**119**	*213*	**110**	**109**	**99**	**98**	**92**	**99**	**103**	**98**	*133*		**107**					*141*		**85**		
HNO_3:HCl 3:6	Metranal-18							**117**		*123*	**117**	**105**		**95**	**107**	**98**	**99**	*130*				**118**	*143*		*610*		*155*		
HNO_3:HCl 3:6	Metranal-18							*122*		*123*	**117**	**111**		**98**	**109**	**104**	**106**	**111**				*139*	*143*		*633*		*136*		
HNO_3:HCl 3:6	Metranal-1									**106**	**97**	**98**		**107**	**108**	**98**	**104**	*122*	*182*		**111**	*125*	*137*	*123*	**105**	*147*	**114**	**102**	

Table S9 continued.

HNO_3:HCl 3:6	Metranal-1									**108**	**93**	**91**		**97**	**102**	**95**	**98**	**119**	*205*		**104**	121	*134*	*123*	**105**	*135*	**105**	**98**	
HNO_3:HCl 3:6	NWHR-1				**119**			*130*			**114**	*121*	**115**	**108**	**108**	**103**	**101**	**117**		**119**	**115**	**112**	*151*		*134*	*131*	**118**		
HNO_3:HCl 3:6	NWHR-1				*122*			*137*			**117**	*125*	**119**	**112**	**112**	**109**	**109**	*124*		*123*	*127*	**119**	*158*		*135*	*130*	*126*		
HNO_3:HCl 3:6	NWWQB-1	*132*		*164*	*135*	*172*		**119**		*169*	*141*	**112**	**115**	**115**	**115**	**116**	**118**	*133*	**118**	*159*	*150*	120			*133*	*125*	*122*		*150*
HNO_3:HCl 3:6	NWWQB-1	*138*		*175*	*139*	*188*		120		*177*	*146*	**114**	**118**	**117**	**117**	**117**	**120**	*133*	**118**	*164*	*126*	*121*			*135*	*124*	*123*		**101**
HNO_3:HCl 2:6	CRM-MS-S	**112**	*188*	**112**	**113**	*128*	*127*	*131*	*294*	*141*	*141*	**120**	**103**	**115**	120	**115**	**110**	*150*		*124*		**104**					**111**		
HNO_3:HCl 2:6	CRM-MS-S	**111**	*185*	**116**	**116**	**111**	**116**	*137*	*226*	*135*	*130*	*122*	**106**	**116**	**116**	**120**	**110**	*155*		*126*		**100**					**104**		
HNO_3:HCl 2:6	Metranal-18							*134*		*159*	*150*	*126*		**116**	*130*	**118**	**116**	**118**				*130*					*149*		
HNO_3:HCl 2:6	Metranal-18							*125*		*135*	*128*	**113**		**105**	**117**	**111**	**108**	**110**				*140*					*132*		
HNO_3:HCl 2:6	Metranal-1									*133*	**114**	**106**		**118**	**120**	**105**	**111**	*131*	*197*		*128*	*126*		*130*		*129*	**111**		
HNO_3:HCl 2:6	Metranal-1									*129*	**114**	**105**		**115**	**118**	**105**	**109**	*125*	*189*		*127*	*132*		*131*		*128*	**111**		
HNO_3:HCl 2:6	NWHR-1				*130*			*137*			*122*	121	121	**105**	**112**	**95**	**97**	**114**		120	**108**	**96**				**97**	**104**		
HNO_3:HCl 2:6	NWHR-1				*138*			*145*			*129*	*127*	*128*	**111**	**120**	**100**	**103**	*122*		*129*	120	**101**				**117**	**109**		
HNO_3:HCl 2:6	NWHR-1				**116**			*131*			*130*	*131*	**111**	*123*	**118**	**110**	**108**	*121*		*122*	*132*	**113**					**110**		
HNO_3:HCl 2:6	NWWQB-1	*139*		*194*	*141*	*197*		*142*		*209*	*161*	**116**	**111**	*121*	120	**114**	**113**	*129*	**116**	*189*	*135*	**110**				**106**	**112**		**93**
HNO_3:HCl 2:6	NWWQB-1	*142*		*182*	*141*	*185*		*149*		*195*	*160*	**119**	**113**	*126*	*125*	**120**	**117**	*131*	**116**	*180*	*139*	**114**				**108**	**118**		**104**

Table S 10: Recoveries in % related to the referenced total digestion values of different HNO_3 and HCl ratios. HNO_3:HCl 6:2 means that 6 ml HNO_3 and 2 ml HCl were used for digestion at 150 °C and a hold time of 20 min. Recoveries > 120% are marked in italic font. Acceptable recoveries of between 80 and 120% are marked in bold font (n=7).

Digestion	CRM	Li	Sc	V	Cr	Mn	Fe	Co	Ni	Cu	Zn	Ga	As	Se	Rb	Sr	Mo	Ag	Cd	Sn	Sb	Cs	Ba	La	Ce	Pr	Nd	Sm	Eu	Gd	Tb	Dy	Ho	Er	Tm	Yb	Lu	Hf	Hg	Pb	Bi	Th	U
HNO_3	BCR 667		53		77	**98**	**87**	**98**	**91**	**103**	**82**			36					**86**		1	47		39	44	49	50	69	71	74	73	75	63	55	51	46	44			**96**		65	40
HNO_3	BCR 667		54		78	**97**	**87**	**97**	**91**	**103**	**81**			36					**87**		1	47		39	44	49	50	68	71	74	72	75	62	55	51	46	44			**96**		66	40
HNO_3	Metranal-1			43	64	**86**		72	72	**84**	**85**		**86**	**115**			41	*213*	**106**				49																	**96**			
HNO_3	Metranal-1			46	67	**87**		73	74	**82**	**86**		**86**	**117**			43	*189*	**103**				50																	**97**			
HNO_3	BCR 667		53		68	**97**	**90**	**86**	**88**	**102**	**91**			36					**101**		1	51		41	46	54	56	74	79	**81**	79	72	66	57	63	51	47			**106**		63	41
HNO_3	JSd-3	35	35	54	41			**83**	**84**	**106**	70	35	**107**	*144*	35	32		*234*	**115**	4	1	73	29	**82**	**89**	*126*	**93**	**88**	**87**	**94**	**94**	**86**	76	**81**	77	51	47			**119**	70	72	38
HNO_3	JSd-3	35	35	54	40			**82**	**82**	**105**	70	34	**107**	*141*	34	31		*222*	**114**	5	1	72	28	**81**	**88**	*126*	**93**	**87**	**86**	**94**	**94**	**86**	75	80	77	50	47			**119**	74	72	37
HNO_3:HCl 6:2	Metranal-1			64	75	**91**		**80**	**82**	**86**	**82**		**92**	*124*			**92**	*148*	**93**																				**118**	**93**			
HNO_3:HCl 6:2	Metranal-1			70	**81**	**99**		**88**	**90**	**97**	**89**		**100**	*133*			**108**	*154*	**99**																				*135*	**103**			
HNO_3:HCl 6:2	BCR 667		70		**92**	**109**	**103**	**102**	**105**	**109**	**96**			37					**86**		64	74		57	60	68	68	**80**	79	**86**	75	72	64	57	54	47	47			**91**		64	44
HNO_3:HCl 6:2	BCR 667		71		**94**	**107**	**102**	**100**	**104**	**107**	**94**			37					**86**		63	75		57	59	68	67	**81**	80	**84**	77	72	64	58	55	48	46			**90**		65	44
HNO_3:HCl 6:2	JSd-3	75	59	**81**	70			**92**	**101**	**114**	**82**	69	**119**	*148*		39		*251*	**106**		75	**84**		**105**	**111**	*155*	**114**	**106**	**100**	**107**	**105**	**93**	80	**86**	78	53	49		*122*	**112**		78	41
HNO_3:HCl 6:2	JSd-3	74	57	78	69			**88**	**97**	**109**	79	67	**110**	*143*		38		*241*	**101**		73	**82**		**99**	**105**	*146*	**108**	**102**	**95**	**103**	**102**	**90**	77	**83**	75	50	50		**112**	**107**		73	39
HNO_3:HCl 9:3	Metranal-1			46	65	**83**		70	73	79	**81**		**90**	**112**			75	**91**	**82**				43																*129*	**82**			
HNO_3:HCl 9:3	Metranal-1			53	68	**85**		73	76	**82**	**83**		**92**	**114**			77	**83**	**83**				47																**119**	**86**			
HNO_3:HCl 9:3	BCR 667		61		73	**101**	**94**	**91**	**94**	**101**	**94**			35					79		54	58		48	50	57	58	73	72	76	72	73	60	54	50	45	41			**92**		70	42
HNO_3:HCl 9:3	BCR 667		66		75	**98**	**94**	**89**	**92**	**98**	**91**			37					76		54	65		50	52	59	59	74	73	76	73	74	60	53	50	46	42			**91**		71	42
HNO_3:HCl 9:3	JSd-3	46	47	61	51			80	**86**	**103**	77	57	**110**	*139*	48	37		**95**	**94**	34	62	**81**	35	**90**	**98**	*132*	**97**	**91**	**86**	**95**	**93**	**89**	71	75	66	45	43	6	**100**	**105**	56	76	35
HNO_3:HCl 9:3	JSd-3	43	45	59	49			79	**83**	**103**	76	53	**109**	*138*	44	36		**92**	**96**	33	61	76	32	**87**	**96**	*128*	**94**	**89**	**84**	**92**	**90**	**87**	67	71	65	44	42	5	**97**	**101**	60	73	33
HNO_3:HCl 6:3	Metranal-1			53	69	**87**		75	77	**85**	**85**		**93**	**120**			**101**	**87**	**94**				50																**111**	**88**			
HNO_3:HCl 6:3	Metranal-1			56	71	**88**		76	78	**87**	**86**		**91**	*122*			**97**	**80**	**93**				50																**109**	**89**			
HNO_3:HCl 6:3	BCR 667		65		76	**103**	**97**	**93**	**96**	**104**	**95**			37					**89**		58	66		57	59	61	61	76	76	79	74	76	62	55	51	46	44			**94**		70	41
HNO_3:HCl 6:3	BCR 667		72		79	**100**	**97**	**91**	**95**	**100**	**93**			37					**86**		59	74		60	62	64	63	79	77	**81**	76	77	63	57	52	47	44			**94**		73	42
HNO_3:HCl 6:3	JSd-3	44	45	59	49			79	**84**	**105**	**80**	50	**101**	*137*	39	36		**89**	**98**	34	64	77	35	**97**	**103**	*133*	**97**	**92**	**85**	**94**	**93**	**90**	69	73	65	45	43	6	**101**	**104**	58	76	36
HNO_3:HCl 6:3	JSd-3	45	46	61	51			**80**	**87**	**106**	**82**	50	**104**	*142*	41	37		**97**	**99**	35	64	79	35	**96**	**103**	*133*	**98**	**93**	**87**	**95**	**93**	**90**	70	74	66	45	43	6	**99**	**103**	57	76	35
HNO_3:HCl 8:4	Metranal-1			57	76	**95**		**83**	**85**	**94**	**94**		**103**	*126*			**106**	**88**	**104**				50																*133*	**99**			
HNO_3:HCl 8:4	Metranal-1			63	78	**95**		**83**	**86**	**94**	**93**		**105**	*133*			**92**	**85**	**104**				52																**115**	**96**			

Table S10 continued.

HNO_3:HCl 8:4	BCR 667		70		**84**	**108**	**104**	**100**	**103**	**111**	**101**			38					**92**		63	71		61	63	68	67	**85**	**83**	**87**	**82**	**83**	67	60	56	50	47			**101**		74	45
HNO_3:HCl 8:4	BCR 667		73		**84**	**104**	**101**	**97**	**100**	**107**	**99**			38					**93**		62	75		61	62	69	68	**85**	**83**	**86**	**82**	**83**	67	60	55	50	47			**100**		73	45
HNO_3:HCl 8:4	JSd-3	55	55	69	60			**84**	**92**	**109**	**87**	61	**115**	*146*	53	39		**91**	**105**	36	70	80	41	**96**	**99**	*148*	**108**	**103**	**95**	**105**	**104**	**100**	78	**81**	73	51	48	7	**103**	**111**	59	77	38
HNO_3:HCl 8:4	JSd-3	55	56	69	60			**84**	**91**	**110**	**89**	58	**110**	*147*	52	41		**92**	**104**	38	67	**84**	43	**99**	**101**	*143*	**105**	**99**	**93**	**102**	**100**	**98**	77	**82**	73	50	48	8	**106**	**111**	58	78	39
HNO_3:HCl 3:6	Metranal-1			63	76	**95**		**86**	**85**	**92**	**93**		**102**	*132*			**95**	**87**	**107**				55																*134*	**101**			
HNO_3:HCl 3:6	Metranal-1			64	73	**88**		78	**80**	**89**	**88**		**99**	*149*			**89**	**87**	**104**				55																*124*	**93**			
HNO_3:HCl 3:6	BCR 667		73		**82**	**103**	**100**	**97**	**99**	**107**	**96**			39					**95**		69	67		61	62	71	70	**87**	**86**	**89**	**83**	**92**	69	61	59	52	48			**105**		77	47
HNO_3:HCl 3:6	BCR 667		56		63	**83**	78	76	78	**85**	78			32					75		56	51		46	47	54	52	66	64	67	63	72	53	47	44	40	37			**81**		58	36
HNO_3:HCl 3:6	JSd-3	49	52	67	55			**84**	**89**	**108**	**97**	57	**111**	*145*	46	41		**94**	**106**	36	68	78	38	**97**	**104**	*141*	**103**	**97**	**91**	**101**	**99**	**92**	74	77	71	48	46	5	**109**	**110**	56	75	38
HNO_3:HCl 3:6	JSd-3	62	62	77	63			**87**	**94**	**115**	**104**	67	**117**	*163*	58	48		**103**	**115**	43	79	**87**	51	**104**	**106**	*153*	**112**	**105**	**100**	**110**	**110**	**112**	**82**	**88**	**80**	54	52	7	*128*	*122*	62	**86**	44
HNO_3:HCl 2:6	Metranal-1			79	**90**	**103**		**95**	**94**	**99**	**99**		**109**	*143*			**110**	**92**	**108**																				**118**	**98**			
HNO_3:HCl 2:6	Metranal-1			76	**90**	**102**		**92**	**93**	**98**	**98**		**104**	*137*			**109**	**94**	**113**																				**117**	**98**			
HNO_3:HCl 2:6	BCR 667		**81**		**97**	**97**	**102**	**92**	**103**	**97**	**86**			38					78		72	**82**		68	70	73	74	**89**	**84**	**84**	75	73	66	58	53	49	47			**91**		66	47
HNO_3:HCl 2:6	BCR 667		77		**93**	**99**	**103**	**93**	**105**	**100**	**88**			38					**82**		73	76		65	69	71	72	**87**	**83**	**83**	75	73	65	58	53	49	46			**93**		66	48
HNO_3:HCl 2:6	JSd-3	63	55	77	63			**88**	**92**	**110**	**96**	63	**107**	*143*		43		**88**	**97**		78	**84**		**97**	**104**	*136*	**103**	**97**	**89**	**95**	**90**	**82**	72	75	68	47	46		**96**	**101**		65	36
HNO_3:HCl 2:6	JSd-3	**81**	67	**86**	74			**92**	**94**	**111**	**101**	78	**108**	*143*		46		**90**	**100**		78	**92**		**101**	**109**	*142*	**107**	**99**	**91**	**97**	**92**	**85**	74	78	70	50	49		**100**	**100**		68	38

8.4 Time and temperature

Table S 11: Concentrations in mg/kg of all elements measured > LOQ and with a RSD < 10% in sediment CRM digests with a $2HNO_3$:1HCl mixture (optimised digestion protocol) using different temperature/time-programs with the microwave.

Temperature | Time	CRM	Li	Be	B	Na	Mg	Al	Si	P	S	K	Ca	Sc	Ti	V	Cr	Mn	Fe	Co	Ni	Cu	Zn
**125°C | 20 min**	**CRM-MS-S**	47.8	0.95	37.6	15100	7850	27400	253	1240	10900	3180	35200	6.61	342	61	48.3	341	29200	6.88	17.6	43.4	144
**125°C | 20 min**	**CRM-MS-S**	43.9	0.983	32.3	15300	8010	25600	314	1360	12100	3060	38700	7.1	230	64.1	52.4	353	29800	6.94	17.3	39.2	140
**125°C | 20 min**	**CRM-MS-S**	39.8	0.845	32.4	14300	7230	21000	237	1230	11000	2860	35400	5.97	241	56.1	42.3	327	27200	6.16	15	36.2	129
**125°C | 20 min**	**Metranal-1**	16.4	1.18	13.5	258	3090	10900	402	3370	3930	2340	23700	3.61	336	36.8	77	1090	20700	10.8	35.6	88.9	493
**125°C | 20 min**	**Metranal-1**	15.4	1.11	10.8	246	3100	10900	313	3090	3650	2050	21800	3.58	280	34.8	72.8	1050	20700	10.7	35.2	84.6	480
**125°C | 20 min**	**Metranal-1**	15.1	1.11	11.8	253	3020	10000	337	3220	3740	2040	22400	3.29	291	33.7	71.9	1110	20800	10.9	35.6	90.7	506
**125°C | 20 min**	**NWWQB-1**	41.7	0.956	9.53	270	8150	21500	138	1460	2610	4030	8520	6.45	194	46.1	43.8	2180	39900	16.4	66.4	86.8	304
**125°C | 20 min**	**NWWQB-1**	42.8	1.04	15.5	284	8220	23400	205	1590	2760	4720	9100	7.05	269	51.3	47.4	2190	40000	16.5	66.4	87.7	304
**125°C | 20 min**	**NWWQB-1**	35.8	0.882	9.12	286	6830	17400	203	1510	2660	3500	8640	5.8	214	42.5	40.7	2000	35000	14.3	57.9	79	272
**125°C | 20 min**	**BCR 667**	29.8	0.871	20.8	11700	12400	14000	335	1660	8510	3810	48300	5.08	101	36.5	80.3	786	32700	18.3	109	70	162
**125°C | 20 min**	**BCR 667**	37.3	1.05	28.4	11700	14600	22600	933	1680	8560	5430	49100	6.83	134	50.6	108	801	35800	19.4	123	70.8	167
**125°C | 20 min**	**BCR 667**	32.9	0.874	24.8	11100	13000	17200	223	1580	8040	4440	46400	5.69	112	40.7	89.4	759	32500	18.1	112	66.4	158
**125°C | 40 min**	**CRM-MS-S**	48.1	0.89	33.4	13600	7150	32600	246	1370	12200	3200	40700	8.44	552	76.4	57.8	336	29100	7.02	19	40.9	138
**125°C | 40 min**	**CRM-MS-S**	44.9	0.802	30.5	13200	6840	28200	232	1260	11200	3000	37100	7.36	418	67.6	50.3	321	27500	6.62	17.4	33.7	128
**125°C | 40 min**	**CRM-MS-S**	46.2	0.852	32.3	13400	7040	31400	185	1250	11300	3070	37200	7.72	468	69.2	53.3	330	28500	6.9	19	35.5	132
**125°C | 40 min**	**Metranal-1**	15.5	1.01	11.7	239	2870	11400	357	3310	3900	2260	23900	3.98	377	40.2	75.9	1000	19800	10.2	33.9	82.1	445
**125°C | 40 min**	**Metranal-1**	18	1.14	14.3	259	3150	13300	336	3250	3910	2690	23800	4.27	454	42.8	77.2	1050	21000	10.8	35.6	87.5	472
**125°C | 40 min**	**Metranal-1**	15.4	0.979	10.7	232	2840	11400	286	3120	3660	2130	22700	3.79	335	37.1	70.9	1010	19700	10.2	33.8	83.5	441
**125°C | 40 min**	**NWWQB-1**	38.3	0.843	11.3	245	7080	22600	116	1430	2530	4390	8370	7.09	215	51.2	44.4	1940	36600	14.9	59.7	85.1	275
**125°C | 40 min**	**NWWQB-1**	39.4	0.908	14.9	265	7160	23000	219	1480	2590	5230	8740	7.34	265	55.7	46	1920	36400	14.6	58.4	79.6	270
**125°C | 40 min**	**NWWQB-1**	41.1	1.02	19.7	285	7870	27800	186	1510	2670	6290	8940	8.44	303	64.9	51.5	1960	38000	15.2	60.7	85	278
**125°C | 40 min**	**BCR 667**	33.8	0.884	21.1	10800	13500	19200	336	1800	9180	4690	53200	7.15	119	51.4	111	780	34700	18.8	117	68.2	172
**125°C | 40 min**	**BCR 667**	38.3	1.02	28.7	11200	14800	26200	185	1790	9100	6240	53400	8.31	173	64.2	126	825	37500	19.9	128	71.4	172
**125°C | 40 min**	**BCR 667**	34.2	0.865	21.3	10700	13200	20400	266	1540	8020	4930	46600	6.44	110	46.9	98.7	775	35000	18.8	119	68.7	164

Table S11 continued.

**125°C | 60 min**	**CRM-MS-S**	49.5	0.929	37.8	13700	7400	37600	190	1230	10900	3290	35300	7.85	618	70.2	55.6	339	29400	7.23	20	38.4	134
**125°C | 60 min**	**CRM-MS-S**	49	0.962	38.1	14700	7750	33100	125	1370	12100	3320	39300	8.1	585	73.3	56.5	356	30500	7.4	19.7	44.2	147
**125°C | 60 min**	**CRM-MS-S**	46.4	0.913	36.5	14400	7640	30800	136	1400	12300	3230	40800	8.09	514	74	56.7	353	29900	7.26	18.8	40.3	142
**125°C | 60 min**	**Metranal-1**	16.7	1.03	13.5	246	2960	11600	302	3050	3620	2510	22100	3.75	381	37.6	71	1010	19900	10.2	33.3	83.1	465
**125°C | 60 min**	**Metranal-1**	17.6	1.08	15	261	3110	13100	326	3010	3630	2810	21500	3.88	436	39	71.8	1030	20500	10.6	36.7	85	451
**125°C | 60 min**	**Metranal-1**	16.5	1.07	14	246	3010	12900	284	3020	3590	2570	22000	3.97	420	40.2	72.1	976	19800	10.1	34.6	81.3	432
**125°C | 60 min**	**NWWQB-1**	41	0.969	18	284	7600	24000	203	1480	2630	6020	8820	7.38	271	57.1	46.6	2060	38100	15.1	61	81.6	280
**125°C | 60 min**	**NWWQB-1**	44.4	1.15	29.2	324	8570	33400	125	1540	2670	8020	8950	9.18	407	72.5	55.9	2060	39600	15.9	63.4	80.8	284
**125°C | 60 min**	**NWWQB-1**	45.8	1.16	29.3	334	8610	32100	221	1520	2680	8610	9050	9.04	370	72.1	55.4	2060	40300	16	63.7	83.3	287
**125°C | 60 min**	**BCR 667**	34.5	0.898	24.8	10500	13100	20000	228	1560	8060	5280	47000	6.43	121	48.9	98.4	744	33600	17.9	112	65.1	154
**125°C | 60 min**	**BCR 667**	39.1	1.08	35.9	9980	14200	30800	264	1510	7740	7260	45400	8.26	297	68.5	122	731	34900	18	118	62.6	158
**125°C | 60 min**	**BCR 667**	37.6	0.984	30.6	11600	13900	22300	350	1810	9340	5910	54600	7.25	173	56.1	109	826	36500	19.5	122	72.1	172
**150°C | 40 min**	**CRM-MS-S**	52.3	1.32	49.6	15600	9130	50900	174	1310	10900	4330	36500	8.44	754	73	70.5	387	32700	8.37	22.8	44.3	147
**150°C | 40 min**	**CRM-MS-S**	60.2	1.57	59.1	18200	10400	55200	175	1570	13300	4940	43900	9.93	833	86.5	79.9	433	37500	9.45	25.8	45.9	172
**150°C | 40 min**	**CRM-MS-S**	46.6	1.22	43.7	14200	8270	43200	264	1240	10500	3580	34900	7.86	642	68.9	65.7	347	29800	7.56	20.7	35.7	138
**150°C | 40 min**	**Metranal-1**	20.9	1.62	24.8	336	4320	18900	405	3190	3630	4040	22400	4.21	592	43.9	83.7	1230	23600	12	38.6	90.9	502
**150°C | 40 min**	**Metranal-1**	18.1	1.46	20.8	282	3820	16500	438	2920	3330	3410	20700	3.87	502	39.9	76.8	1090	21100	10.8	35.1	90.5	447
**150°C | 40 min**	**Metranal-1**	18	1.43	20	295	3910	15100	473	3120	3570	3370	22200	3.85	472	39.7	80.6	1130	22100	11.4	36.1	95.1	480
**150°C | 40 min**	**NWWQB-1**	43.5	1.41	32.9	359	9520	30600	308	1500	2480	8890	8610	7.52	321	60.5	53.2	2250	40500	16.4	63.1	90.4	295
**150°C | 40 min**	**NWWQB-1**	49.5	1.85	64.1	518	11600	50000	199	1490	2420	15600	8520	10.4	605	89.2	68.1	2250	43400	17	64.7	88.9	312
**150°C | 40 min**	**NWWQB-1**	44.1	1.49	38.3	373	9880	34100	295	1430	2350	9840	8130	7.64	349	62	53.3	2250	40800	16.3	62.5	88.5	291
**150°C | 40 min**	**BCR 667**	42.3	1.52	56.7	12600	17800	34300	319	1760	8510	9560	51200	8.06	407	69.9	138	866	39100	20.5	127	70.5	187
**150°C | 40 min**	**BCR 667**	45.9	1.81	72	12600	19000	47500	310	1640	7960	12300	47600	9.32	601	83.7	150	894	41300	21.2	131	71.5	185
**150°C | 40 min**	**BCR 667**	34.7	1.31	46	10700	14800	27200	287	1500	7350	7830	43600	6.73	300	56.8	114	761	33600	17.9	110	61.7	152
**150°C | 60 min**	**CRM-MS-S**	53.3	0.979	39.8	13700	7530	47700	278	1340	11800	4020	38800	9.85	1170	85.9	66.9	349	30900	7.84	22.5	39.6	140
**150°C | 60 min**	**CRM-MS-S**	44.7	0.863	33.9	11600	6430	40400	703	1210	10800	3390	35500	8.75	766	75.2	59.4	300	26400	6.62	18.9	33	120
**150°C | 60 min**	**CRM-MS-S**	47.9	0.903	36.4	12400	6880	43500	93.6	1250	11000	3550	35800	8.9	829	77.5	60.8	319	27900	7.06	20.3	35.3	126
**150°C | 60 min**	**Metranal-1**	18.5	1.02	17.4	263	3060	15700	365	2890	3460	3290	21000	4.37	609	45	71.8	912	18800	9.48	32	75	403
**150°C | 60 min**	**Metranal-1**	20.8	1.18	18.7	284	3400	18200	328	3260	3910	3560	23900	5.07	693	50.8	81.4	1040	21400	10.8	46.9	86.1	454

Table S11 continued.

**150°C | 60 min**	**Metranal-1**	20.6	1.17	19.3	295	3530	17600	271	3310	3920	3610	24100	4.88	699	50.1	80.4	1080	22000	11.3	37.5	89.4	470
**150°C | 60 min**	**NWWQB-1**	49.1	1.38	42.9	395	9690	49200	174	1590	2710	11900	9170	12.3	560	102	69.1	2060	42400	16.4	65.5	84.1	293
**150°C | 60 min**	**NWWQB-1**	48	1.35	48.5	432	9580	47600	208	1510	2600	13300	8790	11.5	612	99	65.2	2050	41800	15.9	63.3	86.1	288
**150°C | 60 min**	**NWWQB-1**	45.2	1.35	50.6	440	9130	45800	133	1410	2430	13700	8160	11.7	654	97.6	64	1880	38600	14.8	57.7	72.3	263
**150°C | 60 min**	**BCR 667**	45.2	1.33	49.7	11100	16600	42900	308	1690	8650	9650	50900	10.4	532	90.5	146	812	39100	19.8	129	68	173
**150°C | 60 min**	**BCR 667**	51.8	1.52	67.3	11900	17100	52600	78	1840	9280	13400	54400	12.1	839	107	158	861	41900	21	136	72.4	184
**150°C | 60 min**	**BCR 667**	43.9	1.23	52.6	10600	15000	40900	236	1670	8540	10100	49900	10.2	606	90.5	139	764	37200	18.7	120	64.5	162
**175°C | 20 min**	**CRM-MS-S**	49.5	1.32	47.7	14600	8540	52000	195	1250	10500	4760	34600	8.76	947	75.9	70.3	385	31700	8.08	22.4	39	139
**175°C | 20 min**	**CRM-MS-S**	53.6	1.44	50.6	16000	9260	56700	239	1390	11600	4720	39200	9.6	782	81.1	80.5	401	34900	8.96	24.8	42.5	158
**175°C | 20 min**	**CRM-MS-S**	59.5	1.63	56.8	17600	10100	63700	196	1460	12400	5540	41600	10.2	953	87.9	81.4	454	37800	9.67	26.9	65.2	168
**175°C | 20 min**	**Metranal-1**	23.4	1.79	27.4	370	4590	24300	535	3330	3840	4990	23800	5.11	718	52.1	93.2	1280	25100	12.7	41.5	93.8	521
**175°C | 20 min**	**Metranal-1**	20.3	1.56	24.6	327	3920	20500	559	2930	3380	4320	21300	4.55	650	46.4	81.7	1100	21400	10.8	35.4	87.5	448
**175°C | 20 min**	**Metranal-1**	32.2	1.89	28.6	511	6150	22000	631	3640	4250	6550	26300	5.11	675	52.4	99.3	1790	34500	17.5	56.9	132	726
**175°C | 20 min**	**NWWQB-1**	43.6	1.72	56.2	450	10300	48500	323	1370	2250	13700	7810	9.9	553	85.8	64	2050	40500	15.6	59.3	81.1	273
**175°C | 20 min**	**NWWQB-1**	52.3	1.99	66.8	539	12300	56700	370	1680	2770	16600	9610	12.3	694	106	79.1	2490	47900	18.5	69.9	95.9	323
**175°C | 20 min**	**NWWQB-1**	46.4	1.78	59.6	486	10900	52200	346	1460	2420	14900	8440	10.8	591	92.6	69	2180	42500	16.5	62.5	85.6	288
**175°C | 20 min**	**BCR 667**	57.5	2.23	86.2	15400	23200	58500	278	1980	9790	15400	58400	11.5	677	104	182	1080	51300	26.1	162	87.6	220
**175°C | 20 min**	**BCR 667**	43	1.67	66.9	11200	17000	47100	259	1570	7760	12300	46300	9.52	650	86.5	146	809	38200	19.4	120	65.7	166
**175°C | 20 min**	**BCR 667**	47.4	1.89	73.2	12600	19000	48200	284	1830	9020	12700	53700	10.6	723	95.9	168	923	42600	21.8	136	73.6	184
**175°C | 40 min**	**CRM-MS-S**	49.7	1.38	48.4	14900	8600	53900	229	1290	10700	4960	36000	9.09	1050	78.8	72.5	377	32000	8.15	22.5	38.1	142
**175°C | 40 min**	**CRM-MS-S**	55.5	1.48	53.3	16300	9610	57700	403	1420	11900	5150	40000	9.8	1150	85.7	77.5	413	35700	9.07	25.2	41.5	155
**175°C | 40 min**	**CRM-MS-S**	60.5	1.68	57.5	17500	10200	66400	176	1560	13100	6100	44300	11.3	955	94.4	91.3	455	38700	9.9	36.7	53.3	174
**175°C | 40 min**	**Metranal-1**	22.6	1.68	26.6	388	4280	25200	432	3060	3530	5260	22100	5.1	757	51.3	87.7	1200	23100	11.7	38.4	88.1	482
**175°C | 40 min**	**Metranal-1**	25.3	1.85	28.3	437	4480	28200	503	3250	3810	6050	23700	5.88	893	58.1	95.3	1210	24400	12.4	39.8	91.3	503
**175°C | 40 min**	**Metranal-1**	21.1	1.62	24.9	350	3910	22700	496	2830	3290	4790	20500	4.71	700	47.4	81.2	1060	21200	11.4	34.6	86.5	438
**175°C | 40 min**	**NWWQB-1**	54.7	2.25	82.8	756	12900	70800	359	1740	2830	22300	9900	15.5	988	131	91.1	2500	49500	18.9	71.3	96	328
**175°C | 40 min**	**NWWQB-1**	53.4	2.09	76.3	617	12700	63600	447	1670	2720	19300	9560	13	825	114	82	2480	49100	18.6	70.5	95.3	325
**175°C | 40 min**	**NWWQB-1**	57.5	2.34	85.8	815	13700	75300	217	1740	2800	23600	9840	15.4	987	130	90.4	2580	51000	19.5	73.8	99.3	335
**175°C | 40 min**	**BCR 667**	40.6	1.59	64.3	10700	16100	45200	198	1420	6890	12100	41400	8.72	629	80.3	133	766	35600	18.1	112	60.2	153

Table S11 continued.

**175°C | 40 min**	**BCR 667**	46.9	1.91	76.6	12300	18500	55100	246	1690	8320	14700	49300	10.9	785	99.3	160	887	42000	21.1	130	70.3	179
**175°C | 40 min**	**BCR 667**	51	2.05	81.9	13200	20000	57200	374	1820	8980	15600	54100	11.6	856	105	173	954	45400	22.7	142	76.4	196
**175°C | 60 min**	**CRM-MS-S**	59.2	1.29	49.5	15500	8860	58100	55.1	1460	12700	5360	41500	11.2	1190	95.3	81	391	34800	8.76	26	45.6	155
**175°C | 60 min**	**CRM-MS-S**	57.3	1.3	50.2	15200	8610	57100	93.7	1400	12400	5150	40200	10.8	1000	89	77.6	384	33600	8.52	24.4	48.3	149
**175°C | 60 min**	**CRM-MS-S**	59.9	1.26	49.5	15700	8890	58800	70.7	1520	13300	5310	43300	11.5	1250	97.2	84.6	395	34900	8.9	25.5	41	164
**175°C | 60 min**	**Metranal-1**	28.1	1.63	28.1	462	4430	29500	471	3360	4000	6150	24300	6.48	1020	62.4	94.6	1170	24800	12.3	42	93.9	531
**175°C | 60 min**	**Metranal-1**	29.3	1.65	28.4	475	4540	29900	284	3520	4250	6110	25400	6.7	1040	64.5	96.9	1220	25400	12.7	42.9	97.1	553
**175°C | 60 min**	**Metranal-1**	26.3	1.49	26.5	406	4170	25800	660	3400	4060	5380	24200	6.18	917	60.1	92.9	1140	23500	11.8	40.3	89.3	487
**175°C | 60 min**	**NWWQB-1**	57.9	1.99	79.1	818	12000	70300	41.2	1720	2870	21800	11700	17.3	1070	140	89.4	2260	47900	18	70.9	85.8	312
**175°C | 60 min**	**NWWQB-1**	54.2	1.85	75.1	783	11100	67100	209	1590	2660	20800	9580	16	991	129	82.8	2110	44400	16.6	65.7	79.5	291
**175°C | 60 min**	**NWWQB-1**	55.4	1.82	75.8	728	11700	66700	970	1660	2780	20500	11000	15.9	1010	129	82.9	2180	45100	17.2	67.9	81.9	299
**175°C | 60 min**	**BCR 667**	52.4	1.65	73.3	12100	17900	56100	191	1650	8360	14700	48300	11.4	810	101	152	844	41100	20.5	134	70.9	177
**175°C | 60 min**	**BCR 667**	54.3	1.77	77.6	12500	18300	57200	107	1800	9080	16000	53300	13.4	927	114	168	875	42600	21.3	138	73.6	187
**175°C | 60 min**	**BCR 667**	56.6	1.79	79.1	13000	19200	58200	67.9	1880	9460	15400	55300	13.2	928	113	175	916	44900	22.6	146	76.8	195

**Temperature | Time**	**CRM**	**Ga**	**Ge**	**As**	**Se**	**Br**	**Rb**	**Sr**	**Y**	**Zr**	**Nb**	**Mo**	**Ag**	**Cd**	**In**	**Sn**	**Sb**	**Te**	**Cs**	**Ba**	**La**	**Ce**
**125°C | 20 min**	**CRM-MS-S**	7.57	1.21	16.2	0.741	201	24.3	147	14.4	5.46	0.483	1.46	0.172	0.291	0.0338	6.36	0.893	0.0197	2.2	37.7	22.7	53
**125°C | 20 min**	**CRM-MS-S**	7.09	1.21	17.7	0.765	250	22.2	163	14.9	4.84	0.486	1.56	0.18	0.336	0.0325	6.06	1.16	0.0279	2.02	37.6	23.3	54.3
**125°C | 20 min**	**CRM-MS-S**	6.33	1.12	16.6	0.728	215	20.8	148	14.5	4.3	0.429	1.43	0.169	0.283	0.0277	6.98	0.832	0.025	1.9	30.8	20.8	49.8
**125°C | 20 min**	**Metranal-1**	4.06	1.04	30	1.43	107	22.5	112	12.2	3.32	0.938	0.982	4.14	3.33	0.0303	6.81	1.91	0.0656	3.44	233	18.9	40.5
**125°C | 20 min**	**Metranal-1**	3.96	1.04	28.2	1.33	98.2	20.4	106	11.9	5.52	1.01	0.949	2.87	3.04	0.0289	5.81	1.93	0.0476	3.22	232	18.6	40.3
**125°C | 20 min**	**Metranal-1**	3.76	0.978	28.4	1.49	52.8	20.6	103	11.7	3.42	0.866	0.967	3.05	3.25	0.0291	6.31	1.94	0.0366	3.27	237	18	38.7
**125°C | 20 min**	**NWWQB-1**	7.51	1.49	25	1.34	63.4	34.4	37.2	20.5	3.68	0.451	1.35	0.993	2.17	0.0616	4.39	1.18	0.151	3.21	264	21.8	50.4
**125°C | 20 min**	**NWWQB-1**	7.69	1.56	26.8	1.48	38.9	38.3	40.5	19.8	4.89	0.45	1.34	0.988	2.18	0.0607	4.62	1.2	0.153	3.41	267	22.2	50.4
**125°C | 20 min**	**NWWQB-1**	6.37	1.33	25.5	1.38	85.6	29.6	36.2	17.8	3.83	0.409	1.22	0.92	2	0.0549	3.98	1.05	0.156	2.8	237	18.6	43.4
**125°C | 20 min**	**BCR 667**	4.11	0.81	20.4	0.666	164	28.9	183	13.1	1.69	0.285	1.03	0.369	0.636	0.0293	3.16	0.542	0.0665	2.73	67.6	10.1	23.7
**125°C | 20 min**	**BCR 667**	5.7	0.906	20.6	0.703	166	40.5	186	15.2	2.99	0.477	1.04	0.368	0.642	0.035	3	0.579	0.0611	3.6	82	12.5	29.8
**125°C | 20 min**	**BCR 667**	4.66	0.802	19.4	0.636	142	34	174	12.7	2.14	0.34	0.984	0.347	0.57	0.0303	3.17	0.546	0.0527	3.14	71.7	10.7	25.6
**125°C | 40 min**	**CRM-MS-S**	7.97	1.14	19.1	0.828	167	25.9	172	14.9	6.85	0.498	1.52	0.19	0.301	0.0349	6.95	0.998	0.0282	2.4	44.7	24.4	57.9
**125°C | 40 min**	**CRM-MS-S**	7.27	1.08	17.8	0.838	179	23.9	157	14.7	6.16	0.481	1.48	0.167	0.29	0.0323	6.93	1.47	0.033	2.24	39.3	22.1	54.2

Table S11 continued.

**125°C | 40 min**	**CRM-MS-S**	7.64	1.12	17.9	0.78	168	24.8	159	15.5	7.08	0.486	1.46	0.187	0.3	0.0338	5.25	1.6	0.0269	2.3	42.9	23.8	57.1
**125°C | 40 min**	**Metranal-1**	4.02	1.01	31	1.43	64.9	22.1	117	12.2	3.91	0.877	0.915	2.85	3.07	0.0317	6.55	2.01	0.0406	3.43	238	19.8	44.5
**125°C | 40 min**	**Metranal-1**	4.59	1.06	30.7	1.38	44.7	25.4	119	13.2	5.91	1.02	1.04	3.59	3.23	0.0331	6.58	2.05	0.0584	3.84	257	22.2	50.4
**125°C | 40 min**	**Metranal-1**	3.98	0.967	29.1	1.39	42.1	21.3	109	12.2	4.91	0.993	0.937	2.86	2.97	0.0285	6.66	1.94	0.0557	3.37	240	19.6	44.3
**125°C | 40 min**	**NWWQB-1**	7.26	1.3	25.4	1.34	49.4	36.3	39.3	19.8	6.32	0.428	1.28	0.872	1.95	0.06	4.32	1.14	0.118	3.3	259	20.8	50.4
**125°C | 40 min**	**NWWQB-1**	7.55	1.33	26.1	1.44	63	40.5	41.6	19.4	6.48	0.467	1.22	0.874	1.9	0.0588	4.33	1.1	0.153	3.52	257	20.7	49.3
**125°C | 40 min**	**NWWQB-1**	8.4	1.43	26.5	1.43	71.2	46.5	46.1	20.8	8.97	0.525	1.27	0.885	1.96	0.0631	4.5	1.14	0.126	3.84	272	22.6	53.1
**125°C | 40 min**	**BCR 667**	5.12	0.786	22.6	0.724	91.8	36.1	204	14.5	2.81	0.402	1.06	0.36	0.614	0.0333	3.45	0.584	0.037	3.37	80.6	12.3	29.8
**125°C | 40 min**	**BCR 667**	6.62	0.879	22.8	0.719	73.7	47.4	206	15.9	3.94	0.543	1.1	0.369	0.653	0.0385	3.75	0.633	0.0635	4.22	96.1	14.2	34.3
**125°C | 40 min**	**BCR 667**	5.36	0.777	19.7	0.628	57.1	38.1	178	14	2.85	0.394	1.06	0.357	0.628	0.0348	3.49	0.607	0.0767	3.53	82.8	12.5	30.6
**125°C | 60 min**	**CRM-MS-S**	8.52	1.21	16.6	0.734	145	27.1	151	14.7	7.66	0.601	1.47	0.161	0.284	0.0346	6.31	0.911	0.0279	2.43	48.3	24	56.2
**125°C | 60 min**	**CRM-MS-S**	8.15	1.22	18.6	0.854	83.7	26.3	168	15.6	6.71	0.554	1.49	0.183	0.316	0.0359	7.06	1.01	0.0153	2.42	44.5	25	58.3
**125°C | 60 min**	**CRM-MS-S**	7.71	1.2	19	0.868	108	25.3	170	16.2	6.29	0.54	1.53	0.173	0.303	0.0315	8.63	1	0.016	2.33	41.1	26	57.7
**125°C | 60 min**	**Metranal-1**	4.26	1.03	28.6	1.37	63	23.6	108	11.9	4.15	0.897	0.874	2.74	3	0.0313	6.26	1.88	0.0407	3.53	230	19.7	43.2
**125°C | 60 min**	**Metranal-1**	4.62	1.11	28	1.36	61.7	25.9	108	12.5	5.22	0.976	0.929	3.13	3.08	0.0327	6.88	2.03	0.0458	3.76	241	21.1	46.7
**125°C | 60 min**	**Metranal-1**	4.45	1.08	28.2	1.36	65	24.2	109	12.7	5.39	0.981	0.901	2.98	2.98	0.0313	6.07	1.92	0.0395	3.58	235	21.5	47.7
**125°C | 60 min**	**NWWQB-1**	8.08	1.39	26.5	1.46	73.6	44.7	41.9	19.9	5.8	0.48	1.3	0.936	2.01	0.061	4.38	1.17	0.113	3.79	267	21.3	51
**125°C | 60 min**	**NWWQB-1**	9.45	1.5	26.5	1.46	45.6	56.3	48.1	20.8	8.64	0.535	1.31	0.911	1.99	0.0653	4.9	1.18	0.0998	4.39	287	24.1	56.9
**125°C | 60 min**	**NWWQB-1**	9.7	1.53	26.6	1.46	82.6	58.6	48.4	21.8	11.2	0.61	1.3	0.927	2.09	0.0664	5.08	1.24	0.13	4.45	289	24.6	55.3
**125°C | 60 min**	**BCR 667**	5.47	0.784	19.7	0.627	124	39.7	179	14.3	2.95	0.39	0.983	0.342	0.601	0.0314	3.42	0.551	0.0527	3.53	77.6	11.7	27.8
**125°C | 60 min**	**BCR 667**	7.35	0.887	19.2	0.604	133	53.2	176	14.5	4.43	0.519	0.958	0.334	0.585	0.0395	3.69	0.594	0.0328	4.49	101	14.3	33.3
**125°C | 60 min**	**BCR 667**	5.88	0.864	23.1	0.73	146	44.7	207	15.9	3.09	0.445	1.1	0.387	0.666	0.0368	3.73	0.646	0.0806	4.01	86.9	12.9	31.2
**150°C | 40 min**	**CRM-MS-S**	10.7	1.45	16	0.801	231	33.1	158	16.4	10.6	0.79	1.46	0.163	0.296	0.0382	8.93	1.01	0.0309	2.92	68.4	29.4	63.2
**150°C | 40 min**	**CRM-MS-S**	11.9	1.62	19	0.949	253	37.8	189	19.4	11.6	0.864	1.62	0.193	0.333	0.0446	7.14	1.4	0.0307	3.35	72.8	32.8	69.6
**150°C | 40 min**	**CRM-MS-S**	9.09	1.2	14.6	0.77	234	28	151	15.5	10.2	0.787	1.31	0.158	0.258	0.0349	8.72	1	0.0527	2.56	54.7	24.8	54.5
**150°C | 40 min**	**Metranal-1**	6.01	1.4	27.9	1.53	62.7	34.2	114	13.8	6.94	1.39	0.964	3.03	3.26	0.0372	8.99	1.95	0.0669	4.94	287	27	57.1
**150°C | 40 min**	**Metranal-1**	5.27	1.18	25.5	1.42	75.8	29.3	105	12.7	7.79	1.36	0.857	2.59	2.91	0.0328	7.35	1.77	0.0512	4.25	255	22.9	48.2
**150°C | 40 min**	**Metranal-1**	5.23	1.22	26.1	1.39	90.1	29.3	110	13.3	7.17	1.36	0.904	2.76	3.16	0.0335	7.34	1.92	0.044	4.28	260	23.3	49.8
**150°C | 40 min**	**NWWQB-1**	9.52	1.61	22.9	1.42	120	58.5	43.6	21.2	12.2	0.734	1.3	0.893	2.03	0.0662	5.26	1.14	0.139	4.71	300	23.7	54.6

Table S11 continued.

**150°C | 40 min**	**NWWQB-1**	13.1	1.84	23.9	1.47	108	90.5	56.5	22.3	23.8	0.797	1.28	0.848	1.95	0.0781	5.88	1.22	0.137	6.13	369	30.1	66.6
**150°C | 40 min**	**NWWQB-1**	10	1.59	22.6	1.39	68.2	62.8	43.3	20.4	12.8	0.691	1.24	0.847	1.96	0.0685	5.12	1.1	0.134	4.93	307	24.5	56.1
**150°C | 40 min**	**BCR 667**	8.25	1.02	18.5	0.683	197	66.1	197	16.9	4.96	0.768	1.03	0.359	0.624	0.0418	4.25	0.603	0.0636	5.59	113	15.1	34.1
**150°C | 40 min**	**BCR 667**	10.5	1.19	19.2	0.716	169	83.4	191	16.8	5.89	0.769	1.04	0.356	0.648	0.049	4.93	0.64	0.0776	6.66	157	18.1	39.8
**150°C | 40 min**	**BCR 667**	6.76	0.857	16.3	0.611	162	54.7	170	14.7	3.92	0.594	0.916	0.314	0.588	0.0349	3.69	0.527	0.0764	4.72	94.1	12.9	28.8
**150°C | 60 min**	**CRM-MS-S**	10.1	1.47	18.7	0.825	96	32	170	16.3	10.3	0.437	1.51	0.174	0.292	0.0397	7.32	1.01	0.0259	2.72	68.9	31.5	66.2
**150°C | 60 min**	**CRM-MS-S**	8.53	1.12	17	0.785	167	27.4	155	13.9	7.88	0.45	1.22	0.145	0.317	0.0323	6.32	0.672	0.0143	2.37	55.2	23.2	54
**150°C | 60 min**	**CRM-MS-S**	8.98	1.18	17.4	0.759	180	28.8	158	15.2	9.23	0.539	1.38	0.156	0.271	0.0311	9.11	0.959	0.0286	2.52	59	26.8	57.4
**150°C | 60 min**	**Metranal-1**	5.01	1.09	27.4	1.31	50.3	29	110	13	7.23	1.01	0.835	2.57	2.75	0.0314	5.97	1.85	0.0418	3.92	232	22.9	50.7
**150°C | 60 min**	**Metranal-1**	5.54	1.14	31.1	1.48	49	31.7	126	13.9	8.51	1.02	0.96	2.98	3.05	0.0356	8	2.01	0.0346	4.37	262	24.5	55.9
**150°C | 60 min**	**Metranal-1**	5.63	1.15	30.8	1.4	/	32.6	125	14.1	6.05	1.1	2.35	3.01	3.19	0.0365	6.8	2.08	0.0302	4.51	272	25.4	57.8
**150°C | 60 min**	**NWWQB-1**	12	1.69	27.7	1.51	83.9	75.9	62.1	24.9	23.2	0.597	1.35	0.885	1.98	0.0764	5.49	1.27	0.106	5.31	353	31.2	69.8
**150°C | 60 min**	**NWWQB-1**	12.4	1.67	26.3	1.43	74.3	82.5	60.7	22.2	19.9	0.608	1.31	0.874	1.98	0.0764	5.46	1.21	0.143	5.5	352	28.5	67
**150°C | 60 min**	**NWWQB-1**	12	1.61	24.3	1.31	67	82.5	60.2	21.3	24.3	0.554	1.21	0.821	1.78	0.0717	5.26	1.11	0.11	5.29	361	29.9	63.8
**150°C | 60 min**	**BCR 667**	9.27	1.01	21.2	0.661	127	69.1	203	16.3	5.98	0.532	1.05	0.355	0.614	0.0451	4.34	0.668	0.076	5.61	136	17.1	39.5
**150°C | 60 min**	**BCR 667**	11.4	1.2	22.9	0.708	77.4	91.9	224	17.9	6.36	0.608	1.09	0.383	0.685	0.0527	5.02	0.712	0.0483	6.98	185	19.5	45.3
**150°C | 60 min**	**BCR 667**	9.11	0.933	21.3	0.662	110	71.9	201	14.8	5.02	0.521	1	0.343	0.618	0.0465	4.29	0.647	0.0693	5.73	137	16.3	37.9
**175°C | 20 min**	**CRM-MS-S**	10.9	1.55	15.5	0.829	177	34.9	154	16.7	12.3	0.594	1.42	0.161	0.289	0.0415	7.3	0.797	0.0371	3	82.8	31.4	65.1
**175°C | 20 min**	**CRM-MS-S**	11.6	1.58	16.9	0.848	211	36	172	18.2	12.6	0.779	1.48	0.175	0.317	0.0453	8.2	1.84	0.0487	3.15	77.9	33.5	69.8
**175°C | 20 min**	**CRM-MS-S**	12.7	1.76	18.8	0.94	205	41.1	182	19.2	13.2	0.733	1.6	0.2	0.365	0.0478	9.37	1.15	0.0369	3.54	92.2	35.4	74.7
**175°C | 20 min**	**Metranal-1**	7.13	1.56	30.1	1.63	57.6	40.7	126	15.5	12.2	1.4	1.05	2.97	3.85	0.0433	8.2	2.16	0.0351	5.48	317	31.4	63.4
**175°C | 20 min**	**Metranal-1**	6.2	1.35	25	1.36	70.8	35.3	113	13.9	11.8	1.34	0.921	2.66	2.93	0.0335	8.57	1.79	0.0621	4.76	277	26.6	55.9
**175°C | 20 min**	**Metranal-1**	9.13	2.01	32.4	1.75	70.2	54.7	136	16.6	10.5	1.52	1.48	4.29	4.72	0.0434	11.1	2.97	0.0563	7.54	432	39	82.7
**175°C | 20 min**	**NWWQB-1**	12.1	1.66	21.7	1.33	72.5	81.1	53.8	21.4	24.3	0.644	1.18	0.789	1.86	0.0723	5.48	1.08	0.143	5.58	347	29.5	63.4
**175°C | 20 min**	**NWWQB-1**	14.4	1.92	25.6	1.52	61.1	96.9	66.4	26.2	28.3	0.757	1.38	0.93	2.19	0.0863	6.51	1.23	0.176	6.77	429	36	76.1
**175°C | 20 min**	**NWWQB-1**	12.9	1.74	24.2	1.42	101	87.3	58.4	23	26.4	0.794	1.24	0.829	1.97	0.078	5.78	1.15	0.159	5.95	370	31.7	67
**175°C | 20 min**	**BCR 667**	13.2	1.37	23.1	0.792	172	104	232	20.4	7.46	0.736	1.24	0.434	0.79	0.0595	5.97	0.767	0.0843	8.28	197	22.3	48.1
**175°C | 20 min**	**BCR 667**	10.2	1.1	17.9	0.648	150	81.7	186	16.7	6.11	0.721	0.94	0.332	0.619	0.0479	4.67	0.582	0.0573	6.39	160	17.5	38
**175°C | 20 min**	**BCR 667**	10.9	1.2	20.3	0.753	91.9	86.8	214	19.1	6.58	0.753	1.08	0.371	0.687	0.0506	5.03	0.647	0.112	7.02	165	19	41.6

Table S11 continued.

**175°C | 40 min**	**CRM-MS-S**	11	1.69	16.2	0.806	68.8	36	159	17.3	12.9	0.266	1.46	0.163	0.304	0.0413	8.02	1.07	0.0162	3.03	83.8	31.4	66.2
**175°C | 40 min**	**CRM-MS-S**	12	1.78	17.7	0.841	60.7	39	177	18.5	13.1	0.314	1.56	0.195	0.322	0.0461	8.26	0.993	0.0442	3.34	86.7	34.7	72.1
**175°C | 40 min**	**CRM-MS-S**	13.5	1.85	19.4	0.945	158	44.8	197	21	15.7	0.514	1.6	0.199	0.354	0.0507	8.61	0.908	0.0465	3.73	106	37.4	79.3
**175°C | 40 min**	**Metranal-1**	7.26	1.64	26	1.37	23.9	42	119	15.3	14.6	1.3	0.932	2.96	3.14	0.0438	7.47	1.97	0.0374	5.39	307	32.6	66.5
**175°C | 40 min**	**Metranal-1**	8.06	1.65	28.5	1.53	71.5	46.4	131	17	24.5	1.54	0.976	3.02	3.43	0.0401	10.7	2.11	0.0708	5.59	338	34.3	67.9
**175°C | 40 min**	**Metranal-1**	6.65	1.48	25.2	1.31	34.3	38.3	111	14.3	14.2	1.29	0.954	2.56	2.95	0.0397	7.09	1.87	0.0495	4.85	280	30	60.9
**175°C | 40 min**	**NWWQB-1**	17.5	2.46	27.6	1.62	49.1	126	83.7	28.7	42.6	0.754	1.42	0.952	2.17	0.0929	7.23	1.36	0.153	7.77	534	43.4	89.7
**175°C | 40 min**	**NWWQB-1**	15.6	2.03	27	1.69	/	111	70.9	26.4	30.5	0.528	1.39	0.95	2.15	0.0875	8.56	1.39	0.152	7.29	469	37.9	80.4
**175°C | 40 min**	**NWWQB-1**	18	2.54	28.8	1.68	83.5	131	83.5	28.4	41.9	0.836	1.47	0.974	2.25	0.0972	7.37	1.35	0.172	8.03	537	44.6	90.6
**175°C | 40 min**	**BCR 667**	9.83	1.04	16.5	0.563	44.3	80.4	169	15.1	5.52	0.469	0.868	0.309	0.585	0.0426	4.46	0.544	0.0533	6.29	162	16.7	36.4
**175°C | 40 min**	**BCR 667**	11.9	1.32	19.7	0.697	74.3	97.4	206	18.3	6.92	0.619	1.04	0.363	0.653	0.0521	5.33	0.637	0.114	7.38	201	20	43.3
**175°C | 40 min**	**BCR 667**	12.6	1.39	21.7	0.767	67.7	104	222	19.8	7.38	0.715	1.13	0.4	0.724	0.0578	5.61	0.751	0.0996	8.09	212	21.7	47.3
**175°C | 60 min**	**CRM-MS-S**	12	1.83	19.3	0.889	84.1	39	183	17.5	13.4	0.157	1.57	0.177	0.318	0.0458	7.34	0.837	0.0293	3.06	93.1	32	67.4
**175°C | 60 min**	**CRM-MS-S**	11.8	1.77	18.8	0.827	98.8	37.9	179	18	14.4	0.443	1.47	0.189	0.301	0.0418	9.21	1.39	0.0207	2.98	90.1	34.4	68.7
**175°C | 60 min**	**CRM-MS-S**	12.2	1.79	20.8	0.919	117	39.4	190	18.3	13.8	0.316	1.62	0.186	0.358	0.0454	7.31	2.1	0.0358	3.12	91.7	33	68.3
**175°C | 60 min**	**Metranal-1**	8.21	1.76	31.8	1.46	/	47.2	134	15.4	22.7	1.26	1.08	3.01	3.33	0.0433	8	2.15	0.0412	5.32	320	31.9	64
**175°C | 60 min**	**Metranal-1**	8.49	1.88	32.9	1.55	0.675	48.2	140	16.7	23.8	1.28	1.2	3.29	3.41	0.0482	8.51	2.29	0.0492	5.47	330	34	68.3
**175°C | 60 min**	**Metranal-1**	7.48	1.56	31.3	1.51	49.1	42.8	133	14.7	19.4	1.14	1.04	2.94	3.15	0.044	7.83	2.07	0.0672	5.02	304	31	62.7
**175°C | 60 min**	**NWWQB-1**	17.5	2.47	28.6	1.57	52.4	125	88.4	25.9	40.8	0.596	1.42	0.955	2.12	0.0916	6.8	1.42	0.153	6.95	509	42.8	86
**175°C | 60 min**	**NWWQB-1**	16.5	2.4	26.4	1.45	55.7	118	82.9	25.5	41.8	0.647	1.31	0.892	1.97	0.0848	6.33	1.26	0.155	6.52	476	41.2	81
**175°C | 60 min**	**NWWQB-1**	16.3	2.44	27.1	1.47	/	116	80.3	25	31.4	0.402	1.37	0.926	2.04	0.087	5.82	1.23	0.0982	6.56	469	40.5	79.5
**175°C | 60 min**	**BCR 667**	11.9	1.26	20.2	0.667	93.7	96.6	202	16.7	6.3	0.606	1.03	0.37	0.641	0.0513	4.96	0.675	0.0571	6.91	198	19.3	42.2
**175°C | 60 min**	**BCR 667**	12.9	1.39	21.9	0.696	45.8	105	221	17	6.38	0.566	1.11	0.378	0.659	0.056	5.37	0.709	0.06	7.33	222	21.2	46.4
**175°C | 60 min**	**BCR 667**	12.7	1.36	22.8	0.745	9.99	103	226	17.6	6.26	0.4	1.15	0.395	0.664	0.0547	5.36	0.72	0.0541	7.44	205	20.7	45.7

**Temperature | Time**	**CRM**	**Pr**	**Nd**	**Sm**	**Eu**	**Gd**	**Tb**	**Dy**	**Ho**	**Er**	**Tm**	**Yb**	**Lu**	**W**	**Hg**	**Tl**	**Pb**	**Bi**	**Th**	**U**
**125°C | 20 min**	**CRM-MS-S**	5.66	22.1	4.46	0.937	3.81	0.509	3	0.536	1.43	0.192	1.27	0.164	0.269	0.125	0.286	78.9	0.262	7.04	3.52
**125°C | 20 min**	**CRM-MS-S**	5.88	23.4	4.62	0.974	3.95	0.527	3.1	0.552	1.48	0.204	1.33	0.172	0.285	0.131	0.279	81.8	0.29	7.52	3.33
**125°C | 20 min**	**CRM-MS-S**	5.31	21.4	4.42	0.892	3.82	0.509	3.02	0.536	1.44	0.188	1.24	0.167	0.281	0.123	0.247	69.2	0.254	6.56	3
**125°C | 20 min**	**Metranal-1**	4.47	18.7	3.86	0.677	3.37	0.433	2.46	0.42	1.09	0.133	0.888	0.118	3.24	1.47	0.323	96.9	0.74	4.62	2.07

Table S11 continued.

**125°C | 20 min**	**Metranal-1**	4.47	18.3	3.9	0.662	3.39	0.434	2.44	0.418	1.08	0.13	0.831	0.114	3.17	1.42	0.306	89.2	0.706	5.19	1.99
**125°C | 20 min**	**Metranal-1**	4.36	18	3.68	0.673	3.23	0.419	2.36	0.409	1.06	0.131	0.868	0.112	3.31	1.39	0.314	95.3	0.731	4.59	2.01
**125°C | 20 min**	**NWWQB-1**	6.28	27	6.41	1.17	5.84	0.766	4.35	0.751	1.94	0.239	1.58	0.212	0.958	1.11	0.923	102	0.716	5.76	2.92
**125°C | 20 min**	**NWWQB-1**	6.29	27.5	6.19	1.18	5.58	0.738	4.17	0.724	1.88	0.24	1.6	0.206	0.956	1.17	0.942	103	0.718	6.3	4.12
**125°C | 20 min**	**NWWQB-1**	5.44	24	5.49	1.04	5.03	0.666	3.74	0.643	1.67	0.212	1.4	0.182	0.914	1.1	0.816	95.1	0.659	4.89	2.86
**125°C | 20 min**	**BCR 667**	2.94	12.7	3.2	0.697	3.26	0.451	2.59	0.453	1.16	0.151	0.96	0.122	0.681	0.127	0.236	33.6	0.507	5.15	0.846
**125°C | 20 min**	**BCR 667**	3.46	14.8	3.9	0.749	3.85	0.526	3.04	0.528	1.36	0.165	1.03	0.143	0.627	0.146	0.308	34.6	0.512	5.99	0.947
**125°C | 20 min**	**BCR 667**	3.07	12.9	3.25	0.69	3.25	0.446	2.59	0.448	1.16	0.152	0.946	0.122	0.659	0.124	0.259	32.3	0.483	5.48	0.857
**125°C | 40 min**	**CRM-MS-S**	6.14	23.3	4.78	0.998	4.05	0.538	3.16	0.56	1.5	0.205	1.33	0.172	0.288	0.128	0.323	78.1	0.274	7.71	3.41
**125°C | 40 min**	**CRM-MS-S**	5.77	21.5	4.62	0.959	3.94	0.525	3.08	0.548	1.47	0.2	1.25	0.17	0.258	0.12	0.293	96	0.269	7.07	3.12
**125°C | 40 min**	**CRM-MS-S**	6.1	22.9	4.95	0.989	4.19	0.558	3.27	0.577	1.55	0.207	1.31	0.178	0.268	0.123	0.304	93.1	0.269	7.61	3.2
**125°C | 40 min**	**Metranal-1**	4.91	18.8	4.14	0.711	3.59	0.458	2.54	0.432	1.12	0.14	0.863	0.118	3.14	1.26	0.319	95.6	0.713	5.49	2.07
**125°C | 40 min**	**Metranal-1**	5.49	20.7	4.61	0.77	3.96	0.504	2.78	0.472	1.21	0.151	0.945	0.129	3.42	1.53	0.352	95.8	0.718	6.74	2.25
**125°C | 40 min**	**Metranal-1**	4.85	18.5	4.18	0.716	3.58	0.462	2.58	0.437	1.14	0.14	0.882	0.121	3.1	1.35	0.317	94	0.7	5.78	2.03
**125°C | 40 min**	**NWWQB-1**	6.16	24.9	6.16	1.15	5.62	0.736	4.2	0.722	1.89	0.238	1.52	0.21	0.935	1.06	0.908	97.8	0.664	6.33	3.12
**125°C | 40 min**	**NWWQB-1**	6.01	24.6	6.01	1.13	5.47	0.716	4.09	0.706	1.84	0.235	1.51	0.202	0.924	1.04	0.923	96.2	0.656	6.18	4.21
**125°C | 40 min**	**NWWQB-1**	6.46	26.2	6.34	1.18	5.71	0.757	4.31	0.75	1.97	0.25	1.61	0.22	0.969	1.05	0.978	96.4	0.669	6.76	3.73
**125°C | 40 min**	**BCR 667**	3.46	13.9	3.72	0.75	3.65	0.501	2.89	0.499	1.29	0.163	1.02	0.135	0.694	0.134	0.291	34.3	0.516	6.03	0.926
**125°C | 40 min**	**BCR 667**	3.95	15.7	4.16	0.819	4.03	0.551	3.18	0.547	1.41	0.181	1.1	0.15	0.726	0.212	0.358	35.3	0.528	6.75	1.02
**125°C | 40 min**	**BCR 667**	3.51	14	3.65	0.761	3.56	0.487	2.83	0.487	1.25	0.167	1.02	0.134	0.697	0.136	0.302	34.3	0.51	6.16	0.949
**125°C | 60 min**	**CRM-MS-S**	6.04	23	4.75	0.975	4	0.525	3.08	0.542	1.46	0.198	1.28	0.166	0.261	0.145	0.319	72.2	0.277	7.34	3.06
**125°C | 60 min**	**CRM-MS-S**	6.24	24.3	4.93	1.02	4.18	0.554	3.23	0.572	1.53	0.206	1.34	0.179	0.201	0.135	0.308	83.3	0.278	7.73	3.42
**125°C | 60 min**	**CRM-MS-S**	6.18	24.2	5.08	1	4.33	0.573	3.34	0.597	1.59	0.204	1.34	0.197	0.195	0.127	0.3	82.1	0.295	7.66	3.26
**125°C | 60 min**	**Metranal-1**	4.71	18.8	3.98	0.677	3.43	0.437	2.45	0.413	1.07	0.133	0.861	0.115	3.17	1.29	0.317	90.9	0.672	5.52	1.98
**125°C | 60 min**	**Metranal-1**	5.14	20	4.3	0.727	3.69	0.468	2.6	0.442	1.13	0.139	0.882	0.12	3.31	1.34	0.34	93.3	0.691	6.31	2.08
**125°C | 60 min**	**Metranal-1**	5.22	20.6	4.51	0.701	3.86	0.485	2.68	0.451	1.16	0.137	0.867	0.122	2.96	1.27	0.333	89.3	0.672	6.49	2.15
**125°C | 60 min**	**NWWQB-1**	6.22	25.9	6.11	1.17	5.54	0.735	4.14	0.722	1.88	0.244	1.56	0.207	0.953	1.07	0.944	97.7	0.671	5.93	3.03
**125°C | 60 min**	**NWWQB-1**	6.77	28.4	6.39	1.22	5.67	0.753	4.32	0.754	1.97	0.256	1.69	0.219	0.837	1.05	1.04	96.5	0.675	7.31	3.24
**125°C | 60 min**	**NWWQB-1**	6.73	28.2	6.6	1.2	5.96	0.78	4.46	0.775	2.04	0.254	1.72	0.227	0.934	1.09	1.07	99.1	0.684	7.36	3.08

Table S11 continued.

**125°C | 60 min**	**BCR 667**	3.23	13.4	3.66	0.703	3.61	0.496	2.89	0.495	1.28	0.154	0.977	0.135	0.625	0.121	0.293	32.5	0.497	5.74	0.891
**125°C | 60 min**	**BCR 667**	3.82	15.3	3.89	0.757	3.69	0.502	2.9	0.501	1.29	0.165	1.03	0.137	0.657	0.119	0.388	31.8	0.47	6.43	0.976
**125°C | 60 min**	**BCR 667**	3.64	15.3	4.03	0.791	3.99	0.545	3.16	0.545	1.41	0.172	1.08	0.15	0.742	0.134	0.323	36.4	0.542	6.38	0.989
**150°C | 40 min**	**CRM-MS-S**	7.26	27.2	5.11	1.06	4.2	0.54	3.2	0.55	1.56	0.209	1.36	0.182	0.239	0.159	0.373	80.9	0.286	8.14	3.39
**150°C | 40 min**	**CRM-MS-S**	7.92	30.3	5.58	1.19	4.64	0.6	3.61	0.625	1.78	0.24	1.54	0.21	0.274	0.179	0.419	90.9	0.301	8.8	3.9
**150°C | 40 min**	**CRM-MS-S**	6.27	23.4	4.51	0.974	3.75	0.486	2.92	0.501	1.44	0.197	1.26	0.171	0.249	0.153	0.321	72.4	0.242	7.17	3.07
**150°C | 40 min**	**Metranal-1**	6.62	24.8	4.76	0.849	3.96	0.489	2.78	0.457	1.26	0.168	1.04	0.137	2.77	1.49	0.41	96.8	0.65	7.47	2.42
**150°C | 40 min**	**Metranal-1**	5.63	22	4.09	0.749	3.44	0.433	2.46	0.411	1.13	0.151	0.942	0.124	2.83	1.51	0.364	87.6	0.64	6.56	2.13
**150°C | 40 min**	**Metranal-1**	5.71	21.8	4.23	0.766	3.56	0.447	2.54	0.421	1.16	0.151	0.944	0.127	3.75	1.44	0.361	91.6	0.702	6.83	2.23
**150°C | 40 min**	**NWWQB-1**	6.96	26.8	6.06	1.23	5.42	0.702	4.16	0.709	1.97	0.259	1.65	0.223	0.912	1.13	1.05	97.2	0.632	7.16	4.65
**150°C | 40 min**	**NWWQB-1**	8.22	32	6.81	1.34	5.82	0.76	4.51	0.776	2.2	0.292	1.87	0.257	0.813	1.12	1.23	94.2	0.62	8.56	3.14
**150°C | 40 min**	**NWWQB-1**	7.13	26.9	6.17	1.26	5.46	0.71	4.19	0.713	1.99	0.263	1.67	0.227	0.846	1.12	1.05	94.5	0.613	7.33	3.63
**150°C | 40 min**	**BCR 667**	4.09	16.1	3.86	0.825	3.71	0.497	2.95	0.498	1.37	0.181	1.13	0.151	0.676	0.198	0.433	34.7	0.488	6.66	1.04
**150°C | 40 min**	**BCR 667**	4.72	18.5	4.24	0.88	3.97	0.531	3.15	0.53	1.47	0.192	1.19	0.161	0.602	0.171	0.564	35.6	0.488	7.12	1.15
**150°C | 40 min**	**BCR 667**	3.54	13.9	3.41	0.729	3.3	0.444	2.64	0.443	1.23	0.162	1.01	0.134	0.585	0.15	0.357	30.7	0.426	5.89	0.894
**150°C | 60 min**	**CRM-MS-S**	7.09	26.2	5.53	1.08	4.57	0.599	3.47	0.605	1.61	0.213	1.38	0.184	0.129	0.137	0.379	85.8	0.286	9.12	3.52
**150°C | 60 min**	**CRM-MS-S**	5.85	21.6	4.59	0.91	3.84	0.505	2.94	0.517	1.38	0.185	1.17	0.159	0.15	0.104	0.309	62.6	0.261	6.96	2.81
**150°C | 60 min**	**CRM-MS-S**	6.13	22.6	5.06	0.969	4.22	0.558	3.24	0.571	1.53	0.194	1.25	0.176	0.223	0.113	0.334	74.3	0.263	7.4	3.03
**150°C | 60 min**	**Metranal-1**	5.52	21	4.73	0.715	3.95	0.497	2.75	0.46	1.18	0.141	0.873	0.125	2.8	1.17	0.348	81.4	0.674	6.53	2.06
**150°C | 60 min**	**Metranal-1**	6.04	22.3	4.93	0.795	4.14	0.523	2.9	0.491	1.26	0.16	0.984	0.135	3.19	1.42	0.389	92.2	0.718	7.15	2.26
**150°C | 60 min**	**Metranal-1**	6.22	23.2	4.95	0.819	4.18	0.525	2.93	0.496	1.26	0.164	1.03	0.135	2.5	1.53	0.4	97.3	0.773	7.48	2.31
**150°C | 60 min**	**NWWQB-1**	8.09	34	7.76	1.36	6.71	0.888	5.13	0.903	2.38	0.296	1.86	0.273	0.853	1.11	1.21	99.2	0.683	8.82	3.74
**150°C | 60 min**	**NWWQB-1**	7.85	29.9	6.85	1.31	5.99	0.784	4.51	0.799	2.1	0.283	1.82	0.242	0.883	1.05	1.21	96.7	0.667	8.33	3.07
**150°C | 60 min**	**NWWQB-1**	7.4	30.7	6.66	1.21	5.71	0.754	4.35	0.768	2.04	0.261	1.7	0.232	0.682	0.98	1.15	88.4	0.612	8.03	2.72
**150°C | 60 min**	**BCR 667**	4.48	16.8	4.45	0.861	4.2	0.567	3.28	0.565	1.46	0.187	1.14	0.157	0.581	0.118	0.497	34.2	0.508	7.17	1.09
**150°C | 60 min**	**BCR 667**	4.99	18.9	4.9	0.955	4.61	0.622	3.59	0.62	1.61	0.205	1.27	0.17	0.558	0.136	0.638	37.4	0.557	7.82	1.19
**150°C | 60 min**	**BCR 667**	4.27	16.1	3.99	0.833	3.8	0.514	2.96	0.512	1.34	0.179	1.13	0.141	0.628	0.115	0.509	33.7	0.497	6.88	1.06
**175°C | 20 min**	**CRM-MS-S**	7.47	26.8	5.21	1.06	4.3	0.555	3.33	0.567	1.61	0.22	1.4	0.19	0.12	0.157	0.389	70.5	0.258	8.43	3.66
**175°C | 20 min**	**CRM-MS-S**	7.95	28.8	5.59	1.17	4.55	0.592	3.51	0.598	1.72	0.233	1.5	0.201	0.214	0.186	0.406	108	0.284	9	3.76

Table S11 continued.

**175°C | 20 min**	**CRM-MS-S**	8.5	30.9	6.04	1.29	4.98	0.646	3.88	0.666	1.91	0.261	1.66	0.226	0.181	0.191	0.448	88.8	0.316	9.46	3.96
**175°C | 20 min**	**Metranal-1**	7.3	28.1	5.35	0.95	4.41	0.547	3.13	0.517	1.42	0.186	1.18	0.156	2.62	1.52	0.47	101	0.735	8.56	2.59
**175°C | 20 min**	**Metranal-1**	6.42	23.7	4.65	0.824	3.84	0.476	2.71	0.449	1.23	0.163	1.01	0.135	2.86	1.42	0.412	87.7	0.618	7.45	2.3
**175°C | 20 min**	**Metranal-1**	9.52	27.7	5.3	0.976	4.46	0.556	3.2	0.529	1.45	0.19	1.19	0.158	4.15	1.68	0.627	143	0.99	8.06	3.56
**175°C | 20 min**	**NWWQB-1**	7.81	29.5	6.34	1.26	5.41	0.701	4.2	0.718	2.05	0.269	1.73	0.238	0.606	1.04	1.17	87.1	0.572	7.98	2.9
**175°C | 20 min**	**NWWQB-1**	9.42	35.1	7.4	1.47	6.35	0.816	4.91	0.842	2.38	0.315	2.02	0.276	0.56	1.21	1.41	106	0.672	9.36	3.39
**175°C | 20 min**	**NWWQB-1**	8.22	32.9	6.66	1.33	5.71	0.738	4.4	0.758	2.14	0.288	1.86	0.253	0.684	1.09	1.22	92	0.601	8.47	3.06
**175°C | 20 min**	**BCR 667**	5.81	22.5	5.16	1.07	4.81	0.643	3.81	0.643	1.78	0.229	1.44	0.193	0.662	0.196	0.701	43.4	0.603	8.61	1.41
**175°C | 20 min**	**BCR 667**	4.46	17.8	3.97	0.835	3.71	0.498	2.96	0.498	1.38	0.177	1.12	0.151	0.499	0.159	0.557	32.7	0.457	6.66	1.07
**175°C | 20 min**	**BCR 667**	4.92	19.4	4.39	0.915	4.13	0.552	3.27	0.557	1.54	0.199	1.25	0.167	0.458	0.19	0.589	36.9	0.505	7.39	1.17
**175°C | 40 min**	**CRM-MS-S**	7.48	27.9	5.15	1.06	4.23	0.546	3.24	0.554	1.58	0.216	1.37	0.186	0.0588	0.192	0.388	74.3	0.269	8.1	3.5
**175°C | 40 min**	**CRM-MS-S**	8.23	30.1	5.67	1.19	4.67	0.599	3.57	0.614	1.74	0.238	1.52	0.204	0.0664	0.19	0.417	81.4	0.298	8.84	3.69
**175°C | 40 min**	**CRM-MS-S**	8.92	33.6	6.24	1.31	5.14	0.665	3.98	0.681	1.94	0.267	1.71	0.23	0.118	0.205	0.472	93.2	0.336	10	4.22
**175°C | 40 min**	**Metranal-1**	7.52	26.4	5.3	0.916	4.32	0.533	3.01	0.494	1.34	0.177	1.1	0.148	2.17	1.52	0.47	93.1	0.662	8.49	2.51
**175°C | 40 min**	**Metranal-1**	7.76	29.1	5.48	0.929	4.46	0.556	3.14	0.516	1.42	0.188	1.2	0.158	2.24	1.54	0.503	99.9	0.703	8.82	2.71
**175°C | 40 min**	**Metranal-1**	6.94	25.8	4.91	0.811	3.98	0.489	2.77	0.454	1.25	0.166	1.03	0.138	2.4	1.36	0.424	88.1	0.865	8.37	2.38
**175°C | 40 min**	**NWWQB-1**	10.9	42.9	7.98	1.56	6.65	0.866	5.19	0.9	2.57	0.348	2.22	0.306	0.38	1.2	1.57	107	0.696	10.3	5.31
**175°C | 40 min**	**NWWQB-1**	9.81	37.8	7.56	1.49	6.4	0.826	4.96	0.854	2.41	0.324	2.06	0.283	0.31	1.19	1.46	106	0.683	9.52	3.47
**175°C | 40 min**	**NWWQB-1**	11	45.9	8.23	1.62	6.87	0.89	5.35	0.931	2.66	0.359	2.3	0.321	0.443	1.21	1.59	108	0.707	10.5	5.21
**175°C | 40 min**	**BCR 667**	4.28	16.4	3.69	0.772	3.43	0.457	2.72	0.458	1.27	0.164	1.04	0.139	0.242	0.155	0.544	30.8	0.425	6.04	0.984
**175°C | 40 min**	**BCR 667**	5.06	20.3	4.35	0.916	4.04	0.54	3.22	0.543	1.51	0.196	1.23	0.164	0.345	0.167	0.651	35.6	0.492	7.21	1.17
**175°C | 40 min**	**BCR 667**	5.54	22.1	4.73	0.99	4.39	0.588	3.5	0.587	1.65	0.215	1.35	0.178	0.394	0.184	0.718	39.2	0.548	7.78	1.29
**175°C | 60 min**	**CRM-MS-S**	7.39	28.2	5.53	1.14	4.64	0.617	3.62	0.644	1.73	0.241	1.58	0.206	0.0569	0.191	0.423	78.8	0.305	8.8	4.29
**175°C | 60 min**	**CRM-MS-S**	7.54	29.7	6.03	1.12	4.98	0.657	3.8	0.663	1.78	0.226	1.51	0.204	0.128	0.225	0.41	90.1	0.304	9.19	3.54
**175°C | 60 min**	**CRM-MS-S**	7.53	29.1	5.98	1.17	4.97	0.657	3.82	0.672	1.8	0.232	1.53	0.207	0.0875	0.168	0.419	108	0.307	8.9	3.62
**175°C | 60 min**	**Metranal-1**	7	27.8	5.36	0.902	4.46	0.564	3.16	0.539	1.41	0.186	1.22	0.157	2.05	1.4	0.511	99.5	0.743	8.3	2.55
**175°C | 60 min**	**Metranal-1**	7.57	29.8	6	0.941	5	0.633	3.5	0.593	1.52	0.191	1.25	0.166	2.04	1.43	0.516	106	0.791	9.22	2.7
**175°C | 60 min**	**Metranal-1**	6.86	26.9	5.3	0.868	4.4	0.556	3.06	0.518	1.34	0.173	1.12	0.148	2.5	1.39	0.471	96.5	0.736	8.49	2.46
**175°C | 60 min**	**NWWQB-1**	10	42.6	7.95	1.48	6.69	0.885	5.17	0.921	2.49	0.338	2.24	0.291	0.423	1.13	1.49	102	0.707	10.4	4.29

Table S11 continued.

**175°C | 60 min**	**NWWQB-1**	9.41	40.3	7.8	1.38	6.55	0.867	5.09	0.908	2.44	0.313	2.13	0.289	0.354	1.06	1.39	93.5	0.651	9.73	3.24
**175°C | 60 min**	**NWWQB-1**	9.32	40.4	7.77	1.41	6.54	0.863	5	0.89	2.39	0.308	2.08	0.276	0.274	1.08	1.4	97.7	0.668	9.88	5.43
**175°C | 60 min**	**BCR 667**	4.78	18.9	4.45	0.891	4.2	0.569	3.29	0.572	1.49	0.19	1.19	0.159	0.537	0.13	0.64	34.9	0.507	7.26	1.11
**175°C | 60 min**	**BCR 667**	5.18	20.1	4.57	0.942	4.27	0.583	3.34	0.577	1.51	0.204	1.29	0.161	0.443	0.14	0.705	36.8	0.533	7.81	1.2
**175°C | 60 min**	**BCR 667**	5.15	20.8	4.73	0.966	4.46	0.6	3.47	0.6	1.56	0.207	1.3	0.165	0.241	0.143	0.688	38.2	0.563	7.99	1.21

Table S 12: Concentrations in mg/kg of all elements measured > LOQ and with a RSD < 10 % in simulated whole water digests with a $2HNO_3$:1HCl (2:1) mixture (optimised digestion protocol) using different temperature/time-programs with the microwave.

Temperature | Time	CRM	Li	Be	Na	Mg	Al	Si	P	S	K	Ca	Sc	Ti	V	Cr	Mn	Fe	Co	Ni	Cu	Zn
2:1 | 150°C | 20 min	CRM-MS-S	41.4	/	15300	7670	40800	55600	1040	11100	3890	33800	7.45	1190	71.2	51.8	316	27900	6.7	15	32.2	/
2:1 | 150°C | 20 min	CRM-MS-S	37.4	/	14900	6920	37000	53700	1050	11300	3430	33000	7.55	1080	71	53.4	281	25000	6.22	13.7	29.3	/
2:1 | 150°C | 20 min	Metranal-1	/	/	916	3590	20200	/	2660	3290	4520	21300	4.58	703	43.9	75.3	942	19700	9.83	30.4	84.1	360
2:1 | 150°C | 20 min	Metranal-1	/	/	1170	3590	23000	/	2580	3220	5510	21200	4.86	839	47.3	/	937	19300	9.61	27.8	82	344
2:1 | 150°C | 20 min	NWWQB-1	40	1.64	1980	10200	52700	83100	1260	2190	16700	12100	10.7	756	92.9	66.2	1880	40200	15.1	53.9	79	222
2:1 | 150°C | 20 min	NWWQB-1	39.4	1.56	2110	10000	54000	92800	1280	2260	17900	13000	11.7	822	100	71.4	1840	40100	14.7	53.1	78.6	212
2:1 | 150°C | 20 min	BCR 667	44.5	/	12400	18200	50400	80400	1560	8160	14100	46800	10.5	804	93.3	154	845	41800	20.8	132	71.7	137
2:1 | 150°C | 20 min	BCR 667	/	/	12600	18400	53500	89100	1680	8580	14800	49700	11.3	887	101	164	853	42800	21.3	133	71.8	134
2:1 | 150°C | 20 min	SdAR-M2	/	4.65	3430	2070	18300	/	301	1040	9470	6320	2.82	562	19.3	8.14	851	14400	11	45.2	256	749
2:1 | 150°C | 20 min	SdAR-M2	/	4.46	4880	2190	24000	47000	298	1020	13700	6940	2.98	671	19.8	8.89	885	14500	11.3	45.5	256	730
2:1 | 150°C | 40 min	CRM-MS-S	42.7	/	14600	7530	46100	63500	1010	9720	4480	27600	7.24	1450	68.7	51.8	316	28600	6.94	18.9	31.1	160
2:1 | 150°C | 40 min	CRM-MS-S	43.9	/	15200	8040	49600	76900	1060	10700	5150	29400	8.61	1900	78.9	60.9	351	30400	7.58	87.8	34.4	/
2:1 | 150°C | 40 min	Metranal-1	/	1.56	1350	3920	26200	40900	2640	3370	6900	18000	5.33	1010	53.2	84.2	976	21300	10.5	31.6	86.6	425
2:1 | 150°C | 40 min	Metranal-1	/	1.54	1270	4060	26600	39300	2660	3250	6550	18300	5.37	968	52	82.8	1010	21600	10.6	33.5	95.7	410
2:1 | 150°C | 40 min	NWWQB-1	42.3	/	2830	10500	63300	121000	1310	2380	22000	11800	13	1000	112	78.5	1850	41400	15.2	59.7	80.5	276
2:1 | 150°C | 40 min	NWWQB-1	47.2	/	3400	11500	72600	135000	1470	2440	24800	11000	14.6	1220	124	85.5	2120	45900	17.3	64.5	86.7	256
2:1 | 150°C | 40 min	BCR 667	50.3	/	12700	18100	55400	89300	1470	7250	15800	41700	10.4	876	92.6	149	840	42200	20.9	137	70	137
2:1 | 150°C | 40 min	BCR 667	48.2	/	12800	18200	57600	111000	1690	8310	16700	48200	12.3	1120	109	175	841	42200	20.5	132	70.5	170
2:1 | 150°C | 40 min	SdAR-M2	/	4.58	5650	2080	27100	64700	305	1100	15500	4970	3.4	740	21.2	8.92	853	14300	10.8	43.1	254	774
2:1 | 150°C | 40 min	SdAR-M2	/	4.26	5060	1960	25700	56900	261	983	14500	3630	2.84	622	18.6	/	803	13400	10.3	43	241	657
2:1 | 175°C | 20 min	CRM-MS-S	/	1.1	14800	7240	42800	69600	1100	10400	4370	28800	7.83	1600	73.9	98	300	27500	6.95	16.5	33.9	/
2:1 | 175°C | 20 min	CRM-MS-S	/	1.15	14900	7690	49900	81000	1090	11100	5010	32100	8.94	1900	80.3	60.9	328	29400	7.37	17.8	33.5	162
2:1 | 175°C | 20 min	Metranal-1	/	1.58	1480	4150	29300	48700	2840	3390	7590	18200	6.1	1280	57	88.8	1030	22000	11.1	34.4	87.8	435
2:1 | 175°C | 20 min	Metranal-1	/	/	1850	4530	32900	55800	2810	3400	9430	18500	6.29	1360	59.8	93.2	1140	23900	12.2	39.8	94.7	443
2:1 | 175°C | 20 min	NWWQB-1	/	1.77	3590	11200	73900	139000	1400	2430	25200	14500	14.4	1500	122	83.3	2040	44200	16.2	61.6	84.9	258
2:1 | 175°C | 20 min	NWWQB-1	43	/	2860	10700	68300	130000	1370	2370	23200	11100	13.8	1140	118	80.6	1960	42800	15.9	61.2	82.9	262
2:1 | 175°C | 20 min	BCR 667	44.8	/	12900	18100	56600	103000	1580	7780	16600	44500	11.4	1040	102	164	841	42500	21	134	71.6	139

Table S12 continued.

2:1 | 175°C | 20 min	BCR 667	50.2	/	12700	18600	56800	97800	1520	7610	16600	45600	10.9	966	98.1	155	868	43200	21.3	137	71.7	199
2:1 | 175°C | 20 min	SdAR-M2	/	4.54	6280	2130	30000	75300	303	1090	18000	8680	3.33	/	22	8.7	885	14800	11.3	46.4	270	745
2:1 | 175°C | 20 min	SdAR-M2	10.2	4.55	7170	2130	33000	83600	300	938	20800	5740	3.12	784	20.2	9.42	846	14100	11.1	43.1	246	851
**Temperature | Time**	**CRM**	**Ga**	**Ge**	**As**	**Se**	**Br**	**Rb**	**Sr**	**Y**	**Zr**	**Nb**	**Mo**	**Ag**	**Cd**	**In**	**Sn**	**Sb**	**Cs**	**Ba**	**La**	**Ce**
2:1 | 150°C | 20 min	CRM-MS-S	9.7	2.19	12.7	/	16500	30.7	158	13.9	/	2.91	/	0.193	/	/	/	/	2.79	73.7	25.5	55.2
2:1 | 150°C | 20 min	CRM-MS-S	9.1	/	12.4	/	/	27.8	158	13.7	/	/	1.74	0.189	/	/	5.29	1.45	2.45	65.2	24.5	52.1
2:1 | 150°C | 20 min	Metranal-1	6.18	2.11	20	1.06	/	38	117	12.1	/	/	1.08	2.74	2.56	/	6.83	2.43	4.88	280	23.2	47.6
2:1 | 150°C | 20 min	Metranal-1	6.78	2.13	19.4	/	/	40.7	119	13.3	/	3.5	/	2.56	2.52	/	7.92	2.39	4.86	308	25.7	52.2
2:1 | 150°C | 20 min	NWWQB-1	14.8	3.05	19.5	/	12100	96.4	69.6	20.7	/	/	1.39	0.842	1.61	/	6.99	1.55	6.27	432	26.6	58.5
2:1 | 150°C | 20 min	NWWQB-1	15.1	3.22	18.6	1.02	/	99.9	81.4	21.7	23.5	/	1.39	0.844	1.68	0.08	6.42	1.94	6.18	448	28.4	61.2
2:1 | 150°C | 20 min	BCR 667	13.4	/	16.5	/	13000	98.3	207	16.4	/	/	/	0.397	/	/	6.04	0.975	7.81	216	18.4	38.5
2:1 | 150°C | 20 min	BCR 667	14.4	2.81	16	/	14200	101	221	17.8	/	/	1.84	0.388	0.622	/	6.4	1.62	8.17	228	19.5	41.1
2:1 | 150°C | 20 min	SdAR-M2	5.87	2.31	67.2	/	/	46.1	50.7	22.7	/	/	12	13.5	/	2.34	2.05	105	1.36	326	46	102
2:1 | 150°C | 20 min	SdAR-M2	7.45	2.93	68.1	/	/	60.3	65.1	24.5	/	12.4	12.1	11	5.3	2.34	/	111	1.45	444	48.3	108
2:1 | 150°C | 40 min	CRM-MS-S	10.5	2.26	12.6	/	10900	33.7	145	13.4	/	4.07	1.72	0.16	/	/	4.64	/	3.02	94.9	28.2	59.9
2:1 | 150°C | 40 min	CRM-MS-S	11.4	2.05	12.4	/	11200	36.2	165	15.2	/	5.03	2.47	0.165	/	/	4.4	/	3.17	115	29.3	62.2
2:1 | 150°C | 40 min	Metranal-1	7.18	/	20.6	/	/	46.9	125	13.5	24.7	/	/	2.67	/	/	14.3	2.1	5.66	349	27.8	56.9
2:1 | 150°C | 40 min	Metranal-1	7.46	2.42	21.6	/	11000	48.2	124	12.9	26.1	3.77	1.2	3.63	/	/	6.72	2.31	6.07	368	24	49.3
2:1 | 150°C | 40 min	NWWQB-1	16.8	3.36	19.4	/	10300	114	107	23.6	29.6	/	/	0.894	/	/	6.24	1.68	7.14	558	36.7	77.3
2:1 | 150°C | 40 min	NWWQB-1	19.2	3.88	20.3	/	10500	128	122	26.4	41	3.81	1.52	0.977	1.93	/	7.31	1.81	7.85	635	43.2	90
2:1 | 150°C | 40 min	BCR 667	14.2	2.38	16.9	/	11000	105	203	16.4	/	/	1.08	0.413	/	0.07	5.39	1.13	8.43	257	21.8	45.6
2:1 | 150°C | 40 min	BCR 667	14.7	2.24	16.7	/	13200	111	240	18.8	/	3.27	/	0.414	0.678	/	5.73	1.23	8.46	280	21.5	45.1
2:1 | 150°C | 40 min	SdAR-M2	8.19	2.35	72.1	/	10900	67.4	74.2	25.3	/	12.1	11.1	14.3	5.83	2.26	/	106	1.45	496	48.6	108
2:1 | 150°C | 40 min	SdAR-M2	7.54	2.19	64.7	/	14100	62	67	21.7	/	11.3	10.8	13.9	5.22	2.15	/	99	1.45	463	47.2	104
2:1 | 175°C | 20 min	CRM-MS-S	9.86	2.02	12.4	/	11400	31.8	152	14.1	/	4.16	2.16	/	/	/	4.27	1.2	2.76	91.5	25.9	54.9
2:1 | 175°C | 20 min	CRM-MS-S	11.5	2.2	13.8	/	11500	35.6	167	15.6	/	5.07	1.84	0.192	/	/	6.4	2.65	3.11	108	29.2	61.6
2:1 | 175°C | 20 min	Metranal-1	8.13	3.76	22.4	/	10400	52.4	137	15.8	28.3	/	2.21	3.26	3.17	/	6.91	2.45	6.23	391	36.9	75.5
2:1 | 175°C | 20 min	Metranal-1	8.72	2.77	23.8	/	/	59	143	14.9	31.7	6.11	1.21	2.89	3.16	/	7.84	2.68	6.98	447	27.6	56
2:1 | 175°C | 20 min	NWWQB-1	19.4	5.07	19	/	40300	128	131	27.7	49.9	4.54	/	0.949	1.9	0.098	7.12	1.83	7.95	650	45.8	95.5

Table S12 continued.

2:1 | 175°C | 20 min	NWWQB-1	18	3.93	20.8	/	/	121	117	25.2	40.4	/	/	0.915	1.81	0.075	6.88	2.47	7.39	601	41.7	86.8
2:1 | 175°C | 20 min	BCR 667	14.4	/	16.2	/	12600	107	222	17.5	/	/	/	0.411	0.667	0.067	5.6	/	8.53	271	/	46.3
2:1 | 175°C | 20 min	BCR 667	14.3	2.4	16.1	/	11300	107	218	17	/	2.92	1.68	0.398	0.644	/	5.82	1.25	8.51	262	22.3	46.2
2:1 | 175°C | 20 min	SdAR-M2	8.61	2.48	69.4	1.46	/	74.4	82.7	26	/	12.3	12.2	11.3	5.84	2.29	/	108	1.65	561	47.5	105
2:1 | 175°C | 20 min	SdAR-M2	9.3	2.36	63.4	/	4770	83.1	88.3	27.2	/	13.3	11.6	11.3	5.3	2.21	2.77	105	1.6	625	42.2	94.2

**Temperature | Time**	**CRM**	**Pr**	**Nd**	**Sm**	**Eu**	**Gd**	**Tb**	**Dy**	**Ho**	**Er**	**Tm**	**Yb**	**Lu**	**W**	**Tl**	**Pb**	**Bi**	**Th**	**U**
2:1 | 150°C | 20 min	CRM-MS-S	6.31	23.7	4.65	0.993	3.83	0.486	3.1	0.51	1.41	0.2	1.26	0.17	20	2.47	58.6	0.308	6.34	3.07
2:1 | 150°C | 20 min	CRM-MS-S	5.93	22.6	4.32	0.971	3.63	0.461	2.92	0.493	1.29	/	1.18	0.161	15.9	2.42	55.2	0.298	5.96	2.75
2:1 | 150°C | 20 min	Metranal-1	5.59	20.5	4.03	0.747	3.42	0.404	2.48	0.405	1.08	0.143	0.967	0.125	15.2	0.477	80.4	0.658	5.9	1.96
2:1 | 150°C | 20 min	Metranal-1	6.03	22.8	4.47	0.853	3.85	0.465	2.88	0.444	1.15	0.162	1.02	0.139	14.1	0.478	80.7	0.656	6.51	2.37
2:1 | 150°C | 20 min	NWWQB-1	7.45	29.4	6.29	1.36	5.6	0.735	4.5	0.764	2.03	0.303	1.85	0.249	11.6	1.25	89.2	0.677	7.46	2.44
2:1 | 150°C | 20 min	NWWQB-1	7.75	30.1	6.33	1.34	5.64	0.733	4.55	0.755	2	0.291	1.84	0.236	11.2	1.27	86.8	0.674	7.44	2.76
2:1 | 150°C | 20 min	BCR 667	4.65	18.3	4.28	0.91	4.11	0.575	3.39	0.565	1.53	0.192	1.24	0.17	11.2	0.722	37.7	0.621	6.73	1.21
2:1 | 150°C | 20 min	BCR 667	5.01	19.4	4.42	0.943	4.24	0.551	3.56	0.563	1.49	0.22	1.36	0.174	10.9	0.723	37.8	0.605	6.98	1.23
2:1 | 150°C | 20 min	SdAR-M2	11.2	38.9	7.16	0.895	5.7	0.725	4.86	0.811	2.38	0.349	2.4	0.314	14.6	2.72	927	1.25	11.5	1.6
2:1 | 150°C | 20 min	SdAR-M2	11.7	40.1	7.23	1.01	5.82	0.749	5.24	0.885	2.53	0.412	2.63	0.377	10.8	2.79	936	1.26	11.2	1.97
2:1 | 150°C | 40 min	CRM-MS-S	6.9	25	4.78	1.1	3.9	0.495	3.19	0.537	1.42	0.193	1.4	0.179	7.43	0.388	62.1	0.299	6.52	3.14
2:1 | 150°C | 40 min	CRM-MS-S	7.25	25.9	4.96	1.13	4.2	0.502	3.36	0.537	1.45	0.225	1.52	0.195	7.6	0.414	63.1	0.291	7.04	3.38
2:1 | 150°C | 40 min	Metranal-1	6.72	24.1	4.68	0.94	3.92	0.473	2.83	0.454	1.21	0.183	1.1	0.144	9.72	0.523	86.8	0.694	6.9	2.47
2:1 | 150°C | 40 min	Metranal-1	5.77	21	4.21	0.912	3.64	0.466	2.77	0.478	1.25	0.18	1.12	0.149	10.3	0.516	84.6	0.742	5.54	2.27
2:1 | 150°C | 40 min	NWWQB-1	9.66	35.5	7	1.57	6.08	0.788	4.87	0.861	2.29	0.338	2.18	0.277	7.34	1.37	88.8	0.686	8.31	2.78
2:1 | 150°C | 40 min	NWWQB-1	11.1	39.8	8.02	1.69	6.8	0.833	5.59	0.9	2.49	0.396	2.49	0.319	7.95	1.58	96.2	0.792	9.44	2.79
2:1 | 150°C | 40 min	BCR 667	5.47	20.9	4.65	1.02	4.38	0.6	3.68	0.616	1.63	0.234	1.44	0.186	7.8	0.718	37	0.582	7.49	1.34
2:1 | 150°C | 40 min	BCR 667	5.32	20.2	4.53	1	4.24	0.603	3.61	0.614	1.65	0.236	1.45	0.183	8.99	0.81	37.6	0.676	7.32	1.27
2:1 | 150°C | 40 min	SdAR-M2	11.7	40.2	7.41	1.03	5.88	0.799	5.2	0.927	2.62	0.4	2.8	0.356	9.47	2.87	913	1.42	12	1.93
2:1 | 150°C | 40 min	SdAR-M2	11.1	37.3	6.55	0.942	5.32	0.707	4.56	0.805	2.27	0.355	2.4	0.31	9.25	2.64	875	1.23	10.7	1.63
2:1 | 175°C | 20 min	CRM-MS-S	6.28	22.8	4.44	0.929	3.71	0.506	3.06	0.527	1.42	0.194	1.31	0.172	6.37	0.38	57	0.285	6.01	3.23
2:1 | 175°C | 20 min	CRM-MS-S	7.07	25.4	4.84	1.09	4.2	0.544	3.36	0.576	1.56	0.225	1.47	0.196	6.17	0.431	81	0.324	7.03	3.27
2:1 | 175°C | 20 min	Metranal-1	8.64	30.5	5.92	1.07	4.72	0.583	3.42	0.549	1.42	0.196	1.26	0.167	9.8	0.571	86.5	0.728	8.87	3.14

Table S12 continued.

2:1 | 175°C | 20 min	Metranal-1	6.5	22.6	4.32	0.949	3.79	0.505	3.09	0.535	1.44	0.218	1.38	0.174	9.44	0.661	105	0.749	6.38	2.53
2:1 | 175°C | 20 min	NWWQB-1	11.7	40.6	7.97	1.66	6.71	0.84	5.69	0.94	2.61	0.39	2.65	0.346	6.69	1.58	94.3	0.732	9.7	3.76
2:1 | 175°C | 20 min	NWWQB-1	10.6	37.3	7.47	1.64	6.29	0.874	5.2	0.945	2.47	0.351	2.28	0.294	6.37	1.46	92.6	0.704	8.94	2.82
2:1 | 175°C | 20 min	BCR 667	5.54	20.2	4.49	1.07	4.26	0.57	3.46	0.612	1.6	0.214	1.41	0.18	6.89	0.769	37.6	0.588	7.33	1.27
2:1 | 175°C | 20 min	BCR 667	5.67	20.3	4.46	0.997	4.31	0.584	3.52	0.596	1.59	0.229	1.47	0.182	6.67	0.771	39	0.598	7.39	1.35
2:1 | 175°C | 20 min	SdAR-M2	11.3	38	7.04	1.05	5.65	0.797	5.23	0.93	/	0.405	2.92	0.374	7.84	2.95	924	1.29	11.2	1.76
2:1 | 175°C | 20 min	SdAR-M2	10	34.5	6.36	1.11	5.64	0.811	5.59	0.982	2.91	0.439	2.96	0.412	7.66	2.96	899	1.23	10.7	1.72

Table S 13: Concentrations in mg/kg of all elements measured > LOQ and with a RSD < 10 % in simulated whole water digests with an aqua regia (AR) mixture using different temperature/time-programs with the microwave.

**Temperature | Time**	**CRM**	**Be**	**B**	**Na**	**Mg**	**Al**	**Si**	**P**	**S**	**K**	**Ca**	**Sc**	**Ti**	**V**	**Cr**	**Mn**	**Fe**	**Co**	**Ni**	**Cu**	**Zn**	**Ga**	**Ge**	**As**	**Se**	**Br**	**Rb**	**Sr**	**Y**	**Zr**	**Nb**
AR | 150°C | 40 min	CRM-MS-S	/	/	17600	8220	39100	65600	971	7960	4920	32200	7.52	1500	62.8	36.3	307	23900	5.88	/	27.8	/	10.2	/	11.5	/	17300	28.7	130	9.62	/	3.89
AR | 150°C | 40 min	CRM-MS-S	/	/	13600	6600	33100	55800	795	6380	4440	26100	6.8	1470	52.9	34.1	297	20000	5.04	/	23.4	167	8.88	/	9.54	/	15200	24.8	107	8.09	/	/
AR | 150°C | 40 min	Metranal-1	1.44	/	1830	4120	22200	39800	2530	2090	7050	20600	5.27	948	42.7	63.8	956	17300	8.78	11.1	73.1	407	7.14	2.41	18.2	/	16300	40.1	98.7	8.81	/	3.56
AR | 150°C | 40 min	Metranal-1	/	/	1640	3490	19400	34800	1970	1820	6240	16300	4.31	819	37	49.8	808	14800	7.54	10.6	64.2	334	6.19	1.92	16.3	/	13300	34.9	87.8	7.53	/	/
AR | 150°C | 40 min	NWWQB-1	1.7	82.9	3520	12500	60700	1E+05	1330	1490	22700	11700	12.9	912	98.7	58.6	2100	40000	14.6	33.9	77.3	280	17.3	3.51	18.6	1.02	17800	103	86.7	16.1	23.1	2.47
AR | 150°C | 40 min	BCR 667	/	71.4	11300	15800	37400	69100	1200	4970	12400	35500	8.56	630	65.7	106	679	30900	15.2	75.4	52.7	165	11.1	1.89	12.2	/	/	71.6	135	9.36	/	1.72
AR | 150°C | 40 min	BCR 667	/	81.8	13600	19000	46200	83600	1430	5710	15500	43400	10.6	818	79.8	131	807	35400	17.7	87	60.2	201	13.1	2.34	14.1	/	17600	86.5	163	11.5	/	/
AR | 150°C | 40 min	SdAR-M2	4.36	/	4920	2120	19200	45400	244	462	12400	4510	2.69	529	14.9	/	795	11700	9.76	20.5	218	731	6.59	1.92	60.3	1.7	/	46.1	45.7	15	/	/
AR | 150°C | 40 min	SdAR-M2	4.71	/	6600	2460	24600	61600	280	416	16400	5190	3	618	16.7	/	894	12700	10.2	22.6	239	770	8.27	2.54	63.8	1.37	16400	60.3	58.2	16.4	/	/
AR | 175°C | 20 min	CRM-MS-S	/	/	16600	7100	32500	56600	852	6950	4240	30400	6.12	1290	52.5	28.7	265	20400	5.11	/	23.1	/	8.6	2.14	10.4	/	19100	23.7	117	8.14	/	3.29
AR | 175°C | 20 min	CRM-MS-S	1.01	/	13600	6640	33700	60400	802	6280	4570	26700	6.46	1590	52.1	31.8	251	20200	4.98	/	22.2	95.8	9.11	2.97	9.57	/	3920	25.2	107	8.79	/	/
AR | 175°C | 20 min	Metranal-1	1.37	/	1650	3660	21100	39800	2090	1800	6680	19400	4.63	907	38.1	52.5	815	15200	7.68	9.06	68.5	358	6.71	2.57	17.6	/	/	38.1	92.1	8.33	/	/
AR | 175°C | 20 min	Metranal-1	/	/	1600	3500	19500	37400	1940	1670	6340	17500	4.35	879	35.8	49.3	767	14500	7.25	8.84	61.8	360	6.24	2.08	15.7	0.82	12900	35.3	85.6	7.75	13.3	3.19
AR | 175°C | 20 min	NWWQB-1	1.93	72.8	3740	11900	60200	1E+05	1270	1290	23600	12000	12.8	1080	98.5	57.8	1980	37400	13.8	33.5	71.7	272	18.2	3.41	17.8	0.92	/	103	91.4	17.4	25.2	2.76
AR | 175°C | 20 min	NWWQB-1	1.69	74.6	3830	12200	59800	1E+05	1310	1330	23600	15000	13.6	1120	99.7	61.6	2020	38000	14	33.4	74.2	274	18.5	3.72	18.1	/	17800	105	95.1	17.2	25.2	3.03
AR | 175°C | 20 min	BCR 667	1.75	68.7	12400	16300	42300	78700	1220	4930	14400	38000	9.43	905	72.7	113	693	30500	15.3	74.3	50.8	156	12.4	2.94	12.2	/	/	79.1	148	10.4	/	/
AR | 175°C | 20 min	BCR 667	1.93	80.4	13600	18200	49400	92100	1330	5350	16200	40300	10.6	1120	82.5	126	768	35300	17.3	85.7	58.8	194	14.6	2.72	14.1	/	15500	91.1	166	11.9	/	3.41

Table S13 continued.

AR | 150°C | 20 min	CRM-MS-S	/	53.2	16500	8050	32100	58900	987	7980	4020	34100	/	972	59.7	/	315	25400	6.5	41.9	36	120	9.34	/	12.5	/	/	26.4	129	9.82	16.7	2.22
AR | 150°C | 20 min	CRM-MS-S	/	/	12600	5640	25300	45200	689	5630	3220	25000	5.47	898	42.6	57.2	216	16900	4.58	27.1	20.7	/	/	1.37	8.33	/	13900	18.8	98.1	6.68	/	2.29
AR | 150°C | 20 min	Metranal-1	1.35	/	1180	3600	15300	30000	2290	1880	4700	19000	4.1	619	35	91.1	895	16500	8.8	44.8	72.9	376	5.54	1.79	17.7	0.78	16400	31.1	85.7	7.47	14.5	/
AR | 150°C | 20 min	Metranal-1	1.34	/	1270	3260	15800	30100	1940	1640	5010	16800	3.85	624	33.1	77.5	795	13800	7.48	35.8	59.4	308	5.26	1.62	15.2	/	/	29.9	79.6	7.1	14.6	2.43
AR | 150°C | 20 min	NWWQB-1	1.73	62.3	2210	10200	44100	88400	1220	1210	16400	10300	11.1	768	78.2	91.4	1810	34900	13.7	67.4	70.4	245	14.4	/	17.3	0.97	17300	83.1	58.7	14	21.5	/
AR | 150°C | 20 min	NWWQB-1	2.13	75	3150	12100	55800	1E+05	1260	1310	21600	10700	12.5	896	96.3	98.8	2040	39600	15.2	72.5	76.8	269	18.2	3.61	18.9	1.2	13900	102	83.7	16.7	27.4	2.32
AR | 150°C | 20 min	BCR 667	1.52	62.5	10900	15500	35100	67900	1150	4680	11500	35500	7.97	600	63	136	674	29800	15.3	105	52.3	139	10.3	1.89	11.9	/	13600	67.9	133	9.27	/	1.77
AR | 150°C | 20 min	BCR 667	1.7	68.7	12300	17400	38400	75300	1300	5220	12600	40300	8.74	656	68.7	152	755	34200	17.6	121	59.8	159	11.3	/	13.6	/	17000	75.3	148	10.4	/	/
AR | 150°C | 20 min	SdAR-M2	/	/	3540	1930	13700	33800	256	290	8800	4610	2.39	454	13	33.2	745	10600	9.25	52.6	212	736	5.04	1.76	56.2	/	14600	34.7	34.5	12.4	/	7.79
AR | 150°C | 20 min	SdAR-M2	5.01	/	5480	2350	21300	54200	283	319	13800	6420	2.92	607	16.7	37	888	13000	11.2	63	234	742	7.35	/	62.9	1.71	/	51.9	51.3	15.6	/	/
AR | 175°C | 20 min	SdAR-M2	5.21	/	8170	2650	36000	98600	/	/	20300	8580	3.73	953	25.1	/	1010	18300	12.5	49.1	279	751	10.5	/	84.6	/	/	89.1	/	2.69	/	/
AR | 175°C | 20 min	SdAR-M2	5.36	/	7920	2330	33600	97800	311	938	19300	/	3.12	727	21.4	11.5	947	15800	12.2	46.9	259	706	10.1	/	81.1	/	/	85.8	80	2.31	/	/

**Temperature | Time**	**CRM**	**Mo**	**Ag**	**Cd**	**In**	**Sn**	**Sb**	**Cs**	**Ba**	**La**	**Ce**	**Pr**	**Nd**	**Sm**	**Eu**	**Gd**	**Tb**	**Dy**	**Ho**	**Er**	**Tm**	**Yb**	**Lu**	**W**	**Hg**	**Tl**	**Pb**	**Bi**	**Th**	**U**
AR | 150°C | 40 min	CRM-MS-S	1.01	0.11	/	/	3.16	0.881	2.02	71.2	19.3	40.9	4.59	19.7	3.22	0.84	2.72	0.422	2.17	0.44	1.23	0.17	1.03	0.14	/	/	1.8	45	0.22	5.16	2.63
AR | 150°C | 40 min	CRM-MS-S	/	0.1	/	/	3.38	0.769	1.74	67.6	16.5	34.6	3.93	16.5	2.78	0.67	2.32	0.345	1.85	0.37	1.06	0.15	0.93	0.12	2.53	/	1.5	37.2	0.18	4.64	2.05
AR | 150°C | 40 min	Metranal-1	0.59	1.99	/	0.032	5.29	2.1	3.97	248	20.3	42	4.71	19.9	3.36	0.72	2.79	0.402	1.95	0.38	1.03	0.13	0.93	0.11	5.77	1.26	0.373	60.1	0.49	5.58	1.78
AR | 150°C | 40 min	Metranal-1	0.52	1.8	1.99	/	4.38	1.45	3.33	221	15	30.4	3.45	14.8	2.52	0.58	2.12	0.32	1.65	0.32	0.9	0.12	0.73	0.1	4.67	1.15	0.319	52.4	0.42	4.04	1.52
AR | 150°C | 40 min	NWWQB-1	/	0.99	/	/	6	1.28	5.56	430	29.9	62.3	7.57	31.8	5.54	1.26	4.6	0.699	3.54	0.71	2.03	0.29	1.68	0.22	3.58	1.29	1.11	72.2	0.58	7.49	2.31
AR | 150°C | 40 min	BCR 667	/	0.26	0.455	/	4.16	0.681	5.09	144	12.9	26.3	3.15	13.5	2.67	0.66	2.61	0.385	2.02	0.39	1.04	0.14	0.91	0.11	2.65	/	0.46	23.4	0.38	5.06	0.81
AR | 150°C | 40 min	BCR 667	1.18	0.3	/	/	4.79	/	6.1	181	16.3	33.3	3.93	17	3.27	0.8	3.07	0.47	2.51	0.48	1.31	0.17	1.09	0.14	3.11	/	0.551	27.6	0.43	6.33	1
AR | 150°C | 40 min	SdAR-M2	8.5	9.23	5.13	1.58	1.57	82.7	0.99	285	32.9	73.8	7.78	30.9	5	0.75	3.92	0.598	3.34	0.67	2	0.29	1.87	0.24	4.36	1.46	1.89	640	0.91	9.31	1.28
AR | 150°C | 40 min	SdAR-M2	10.2	8.64	4.84	1.79	2.55	95.6	1.18	379	37.4	82	8.63	33.3	5.29	0.82	4.17	0.644	3.6	0.73	2.2	0.33	2.05	0.27	3.96	1.67	2.23	737	1	9.68	1.38
AR | 175°C | 20 min	CRM-MS-S	1.39	0.1	0.193	/	6.49	/	1.78	62	16.8	35.7	4.04	16.6	2.82	0.71	2.34	0.347	1.87	0.36	1.02	0.15	0.89	0.12	1.82	/	1.61	43.3	0.2	4.32	2.12
AR | 175°C | 20 min	CRM-MS-S	0.8	/	/	/	8.21	/	1.83	76.8	16.9	35.3	3.96	16.3	2.82	0.71	2.4	0.353	1.95	0.38	1.1	0.15	0.96	0.13	1.83	/	1.52	36.4	0.19	4.61	2.07
AR | 175°C | 20 min	Metranal-1	/	2	/	/	5.67	1.38	3.61	240	17.2	34.1	3.92	15.6	2.72	0.66	2.33	0.339	1.77	0.35	0.98	0.13	0.85	0.1	4.23	1.35	0.378	57.9	0.46	4.18	1.53
AR | 175°C | 20 min	Metranal-1	/	1.65	2	/	12.5	/	3.37	231	14.1	28.7	3.29	13.3	2.35	0.57	2.04	0.304	1.62	0.32	0.93	0.13	0.83	0.1	4.03	1.04	0.322	52.2	0.42	3.76	1.46
AR | 175°C | 20 min	NWWQB-1	0.9	0.71	1.5	/	6.02	3.08	5.25	442	32.2	66	7.83	33	5.46	1.29	4.65	0.716	3.73	0.77	2.16	0.32	1.88	0.25	/	1.29	1.1	69.3	0.53	7.94	2.73
AR | 175°C | 20 min	NWWQB-1	1.05	0.69	1.67	/	5.61	1.2	5.4	451	32.2	67	7.86	33.2	5.56	1.31	4.72	0.711	3.73	0.78	2.21	0.32	1.94	0.25	2.73	1.45	1.14	71.6	0.54	7.99	2.62
AR | 175°C | 20 min	BCR 667	0.53	0.24	/	/	4.1	/	5.35	177	15.7	32.2	3.73	15.7	2.89	0.7	2.7	0.412	2.21	0.43	1.21	0.18	0.98	0.13	2.56	/	0.501	24.6	0.37	5.35	0.94

Table S13 continued.

AR | 175°C | 20 min	BCR 667	/	0.3	/	/	5.29	/	6.15	213	19.4	39.8	4.55	18.6	3.49	0.85	3.16	0.467	2.55	0.48	1.36	0.18	1.11	0.14	3.01	/	0.555	27.5	0.44	6.21	1.03
AR | 150°C | 20 min	CRM-MS-S	1.59	0.16	0.225	/	4.09	1.31	2.18	54	20.5	43.9	4.94	20.4	3.49	0.86	2.84	0.426	2.27	0.45	1.25	0.18	1.08	0.14	1.66	/	1.92	53.9	0.25	5.9	2.47
AR | 150°C | 20 min	CRM-MS-S	1.08	0.08	/	/	2.82	/	1.46	45.8	13.9	29.5	3.28	14	2.34	0.6	2.01	0.29	1.53	0.3	0.83	0.12	0.75	0.1	1.24	/	1.32	32.6	0.15	3.95	1.69
AR | 150°C | 20 min	Metranal-1	1.03	2.14	2.3	/	4.62	1.51	3.55	208	15.4	31.4	3.54	14.7	2.56	0.6	2.21	0.313	1.59	0.32	0.88	0.12	0.75	0.09	4.05	/	/	59.3	1.01	4.57	1.59
AR | 150°C | 20 min	Metranal-1	/	1.83	1.96	/	4.13	1.49	3.16	189	17	34.6	3.95	16.8	2.89	0.59	2.41	0.335	1.64	0.3	0.83	0.1	0.69	0.09	3.68	0.87	0.283	50.2	0.4	4.84	1.61
AR | 150°C | 20 min	NWWQB-1	/	0.65	/	/	4.89	/	4.69	333	22.7	48.8	5.95	25.3	4.74	1.09	4.17	0.594	3.14	0.6	1.67	0.26	1.36	0.18	2.12	/	0.934	66.4	0.5	6.56	1.9
AR | 150°C | 20 min	NWWQB-1	1.47	0.75	1.84	/	5.63	1.17	5.61	421	30.2	63.3	7.66	31.7	5.72	1.34	4.74	0.692	3.69	0.71	1.99	0.3	1.74	0.23	2.24	/	1.14	78.5	0.54	7.78	2.52
AR | 150°C | 20 min	BCR 667	1.03	0.24	/	0.034	3.69	/	4.82	133	12.3	25.5	3.07	13.3	2.69	0.66	2.54	0.386	2	0.39	1.03	0.14	0.88	0.1	1.76	/	0.438	22.6	0.34	4.99	0.8
AR | 150°C | 20 min	BCR 667	/	0.29	/	/	4.13	0.568	5.51	145	13.5	28.1	3.38	14.3	2.9	0.75	2.86	0.426	2.26	0.42	1.16	0.16	0.99	0.12	/	/	0.489	26.2	0.41	5.52	0.87
AR | 150°C | 20 min	SdAR-M2	8.3	7.29	4.24	1.54	1.17	78.1	0.84	211	29.4	64.2	6.88	27.1	4.37	0.6	3.39	0.516	2.83	0.58	1.69	0.24	1.54	0.21	2.91	0.9	1.77	605	0.86	8.99	1.14
AR | 150°C | 20 min	SdAR-M2	9.95	10.8	4.84	1.8	/	93.5	1.03	327	34.8	77.2	8.01	31.4	5.14	0.84	4.03	0.618	3.51	0.71	2.12	0.31	2.11	0.27	3.89	1.03	2.1	721	0.97	9.91	1.4
AR | 175°C | 20 min	SdAR-M2	11.7	29.3	5.78	2.17	/	113	1.68	620	47.7	/	11.3	44	7.83	1.04	6.47	0.93	6.04	1.11	3.43	0.43	2.88	0.45	3.51	/	2.81	882	1.4	11.6	1.88
AR | 175°C | 20 min	SdAR-M2	11.8	11.1	5.72	2.11	/	113	1.39	594	43.1	/	10.6	40.1	7.28	1.02	5.76	0.81	5.15	0.96	2.88	/	2.6	0.38	3.36	/	2.71	869	1.19	11.7	1.77

9. Case study

9.1 Quality control

Table S 14: Recoveries in % of the simulated whole water digests used as quality control during the case study measurements. Both recoveries of referenced aqua regia extractable and total digestion values (see first column) were considered. Concentrations which were < LOQ or with a RSD > 10% are not included in the dataset. The following elements exhibited only indicative values for SdAR-M2 (marked with asterisks*): Na, P, K, Ti, V, Cr, Fe, Ga, Nb, Ag, Sb, Eu, Gd, Tb, Dy, Ho, Er, Th. Recoveries >120% are marked in italic font. Acceptable recoveries of between 80 and 120% are marked in bold font (n=15).

	CRM	Li	Be	Na*	Mg	Al	Si	P*	K*	Ca	Sc	Ti*	V*	Cr*	Mn	Fe*	Co	Ni	Cu	Zn	Ga*	As	Rb	Sr	Y	Nb*	Mo	Ag*
**Reference is aqua regia**	20221213_WQB-1_1	*134*	*199*	*1151*	*137*	*269*							*224*	*162*	**101**	**112**	**111**	**113**	*124*	**104**							*122*	
	20221213_WQB-1_2	**120**		*794*	*129*	*231*							*205*	*150*	**95**	**105**	**105**	**105**	**115**	**95**		**102**		*202*				**96**
	20221213_WQB-1_3	*127*		*1255*	*136*	*258*							*211*	*148*	**98**	**107**	**104**	**104**	**118**	**101**		**100**		*211*			*121*	**106**
	20221213_SdAR-M2_1	**114**	**104**	*942*				66			*145*	*149*			**101**	**101**	**96**	**99**	**114**	**88**	*227*	**88**		*284*	*127*		**84**	58
	20221213_SdAR-M2_2							70		*244*	*141*	*158*	*124*									**96**	*363*	*297*	*141*			
	20221213_SdAR-M2_3		*120*					71	*525*	*211*			*131*		**104**	**103**	**106**	**106**	**112**	**88**	*227*	**93**	*386*	*320*	*140*	*122*	**81**	**98**
	20221220_WQB-1_1	**115**		*876*	*125*	*214*				*165*					**93**	**99**	**98**	**101**	**111**	**100**								**92**
	20221220_WQB-1_2				*141*	*260*				*174*			*222*	*161*	**99**	**109**	**108**	**106**	**118**	**100**		**104**		*247*				**99**
	20221220_WQB-1_3	*157*		*1769*	*173*	*343*				*219*			*303*		*121*	*133*	*128*	*129*	*144*	**115**		*134*		*395*				*127*
	20221220_SdAR-M2_1		**111**	*849*				71	*448*		*130*	*138*	**111**		**102**	**99**	**91**	**105**	**114**	**91**	*194*	**86**	*309*		*128*		79	64
	20221220_SdAR-M2_2			*1167*				73	*577*		*130*	*158*	**108**		**101**	**96**	**97**	**100**	**110**	**87**	*251*	**84**	*409*	*322*	*144*		78	52
	20221220_SdAR-M2_3		**111**	*1168*				74	*552*		*141*	*155*	*125*		**103**	**99**	**93**	**99**	**110**	**90**	*234*	**92**	*383*	*340*	*139*	*238*	**81**	78
	20220926_WQB-1_1		*225*		*132*	*290*				*161*			*245*		**97**	**110**	**109**	**117**	**106**	**107**		**112**		*245*			*121*	**111**
	20220926_WQB-1_2				*137*	*271*				*163*			*246*	*170*	**99**	**109**	**105**	**109**	**103**	**115**		**114**		*247*				**101**
	20221011_WQB-1_1			*1002*	*136*	*283*				*170*			*244*	*184*	**100**	**111**	**114**	**118**	**108**	**108**		*122*		*263*				**108**
	20221011_WQB-1_2		*163*	*1063*	*135*	*281*				*159*			*251*	*177*	**101**	**112**	**114**	**108**	**108**	**117**		**113**		*255*				**113**
	20221019_WQB-1_1			*1239*	*151*	*298*							*248*		**100**	**112**	**114**	**110**	**109**	*149*								**113**
	20221019_WQB-1_2				*144*	*312*				*169*			*244*	*171*	**97**	**109**	**112**	**101**	**103**	*128*		**105**		*246*				**98**
	20221019_WQB-1_3			*1285*	*144*	*303*				*165*			*247*		**101**	**111**	**112**	**105**	**104**	**114**		*126*		*286*			**108**	**104**
	20220926_SdAR-M2_1			*1112*									*141*		**97**	**96**	**87**	**92**	**92**	**92**	*227*		*389*		*139*		78	*260*
	20221011_SdAR-M2_1		**112**	*859*								*139*	*127*		**101**	**96**	**97**	**103**	**101**	**89**	*218*		*332*		*126*		**82**	60
	20221011_SdAR-M2_2			*983*											**101**	**98**	**98**	**105**	**94**	**97**	*221*						78	64
	20221019_SdAR-M2_1			*984*						*222*	*154*	*146*	*134*		**99**	**99**	**95**	**96**	**98**	**93**	*216*		*340*		*132*		**86**	55
	20221019_SdAR-M2_2		*123*					**81**		*247*		*154*	*133*		**99**	**100**	**87**	**100**	**94**	**96**	*216*	**95**	*362*	*323*	*152*		**88**	66
	20221019_SdAR-M2_3			*1050*				79		*207*	*144*	*155*	*136*		**101**	**102**	**98**	**97**	**95**	**93**	*219*	**91**	*354*		*139*		**90**	80
	20221206_WQB-1_1				*152*	*317*				*135*			*270*	*192*	**111**	*122*	**119**		*130*	*132*		**116**		*275*				*123*
	20221206_WQB-1_2	*134*	*196*		*142*	*293*				*121*			*260*	*180*	**104**	**117**	**109**		*124*	*126*		**112**		*258*				**115**
	20221206_WQB-1_3	*131*	*211*		*133*	*257*				**114**			*240*	*172*	**99**	**108**	**107**		*120*	*121*		**108**		*238*				**108**

Table S14 continued.

	20221206_SdAR-M2_1		*133*						*651*		*173*	*183*	*147*		**109**	**111**	**100**		**113**	**99**	*259*	**91**	*424*	*339*	*145*		**87**	59
	20221206_SdAR-M2_2		*127*					71		*153*	*152*		*129*		**108**	**107**	**98**		**110**	**106**	*214*	**90**	*355*	*264*	*131*	*205*	**86**	58
	20221206_SdAR-M2_3							69	*506*	*169*	*146*		*129*		**106**	**105**	**104**		**110**	**101**	*219*	**86**	*358*	*282*	*126*		75	56
Reference is total digestion	20221213_SdAR-M2_1	**81**	74	24	77	34		58			68	32			**89**	**85**	**99**	**96**	**116**	**90**	41	**92**		37	60		**86**	
	20221213_SdAR-M2_2					36		61		**119**	66	34	73									**101**	38	39	66			
	20221213_SdAR-M2_3		**86**		**86**	35	18	62	31	**103**			77		**92**	**86**	**109**	**102**	**115**	**90**	41	**98**	41	42	66	17	**83**	
	20221220_SdAR-M2_1		79	22	**85**	26		62	27		61	29	66		**90**	**82**	**94**	**101**	**116**	**93**	35	**91**	33		60		**80**	
	20221220_SdAR-M2_2			30		35		64	35		61	34	64		**89**	80	**100**	**96**	**112**	**89**	46	**88**	43	42	68		80	
	20221220_SdAR-M2_3		79	30	**85**	35	18	65	33		66	33	74		**90**	**83**	**96**	**96**	**112**	**92**	43	**97**	41	44	65	33	**83**	
	20220926_SdAR-M2_1			29		35							**83**		**86**	80	**90**	**89**	**94**	**95**	41		41		66		80	
	20221011_SdAR-M2_1		80	22	**83**	32	11					30	75		**89**	**80**	**101**	**99**	**104**	**91**	40		35		59		**84**	
	20221011_SdAR-M2_2			25	76	33									**89**	**82**	**102**	**101**	**96**	**100**	40						**80**	
	20221019_SdAR-M2_1			25	**91**	33				**109**	72	31	79		**87**	**82**	**98**	**93**	**101**	**95**	39		36		62		**88**	
	20221019_SdAR-M2_2		**87**		**82**	34		71		*120*		33	79		**87**	**83**	**90**	**97**	**96**	**98**	39	**100**	38	42	71		**90**	
	20221019_SdAR-M2_3			27	**85**	36	12	69		**101**	68	33	**81**		**89**	**85**	**101**	**93**	**98**	**96**	40	**95**	38		65		**92**	
	20221206_SdAR-M2_1		**95**		**88**	43			39		**81**	39	**87**		**96**	**92**	**103**		**116**	**102**	47	**96**	45	44	68		**89**	
	20221206_SdAR-M2_2		**91**		**83**	37	10	62		75	71		76		**95**	**89**	**102**		**112**	**109**	39	**95**	38	34	62	28	**88**	
	20221206_SdAR-M2_3				**82**	33		60	30	**83**	68		76		**93**	**87**	**107**		**113**	**104**	40	**91**	38	37	59		76	
	**CRM**	**Cd**	**In**	**Sn**	**Sb***	**Cs**	**Ba**	**La**	**Ce**	**Pr**	**Nd**	**Sm**	**Eu***	**Gd***	**Tb***	**Dy***	**Ho***	**Er***	**Tm**	**Yb**	**Lu**	**W**	**Tl**	**Pb**	**Bi**	**Th***	**U**	
Reference is aqua regia	20221213_WQB-1_1	**108**					*184*																	**105**				
	20221213_WQB-1_2						*153*																*159*	**99**			72	
	20221213_WQB-1_3	**109**					*161*																	**102**			77	
	20221213_SdAR-M2_1		**94**		**103**		*299*	**91**	**91**			**98**	*126*	**100**	**107**					*136*	*134*	*991*	*131*	**97**	**103**	**95**	**101**	
	20221213_SdAR-M2_2											**106**			**115**	**112**	*126*				*147*							
	20221213_SdAR-M2_3		**98**		**106**		*305*	**92**	**91**					**107**	**116**	**116**				*123*		*856*	*131*	**100**	*123*	**89**	**103**	
	20221220_WQB-1_1	**99**					*146*																*152*	**93**			67	
	20221220_WQB-1_2						*184*																*182*	**103**				
	20221220_WQB-1_3						*250*																*213*	*122*			**118**	
	20221220_SdAR-M2_1		**100**		**107**	*150*	*268*	**94**	**92**			**93**	*123*	**91**	**98**	**98**	**105**	**106**		**120**	*125*	*454*	*128*	**98**	**101**	**91**	**89**	
	20221220_SdAR-M2_2		**96**		**103**	*176*	*352*	**94**	**91**			**95**	*123*	**93**	**106**	**105**	*128*	**112**		*126*	*135*	*487*	*126*	**97**	**95**	**94**	**94**	
	20221220_SdAR-M2_3	**95**	**95**	**120**	**104**	*158*	*317*	**104**	**102**				*132*	**114**	*123*	**117**		*121*		*126*	*140*	*458*	*125*	**97**	**97**	**104**	**91**	
	20220926_WQB-1_1						*175*																*171*	**109**				
	20220926_WQB-1_2						*172*																	**113**			**93**	
	20221011_WQB-1_1						*181*																*170*	**110**				
	20221011_WQB-1_2	**118**					*186*																*185*	**115**			*137*	
	20221019_WQB-1_1						*192*																*168*	**109**				
	20221019_WQB-1_2						*182*																	**113**			**106**	
	20221019_WQB-1_3						*189*																	**109**			**102**	

Table S14 continued.

	20220926_SdAR-M2_1		**92**		**96**		*310*	**95**	**93**											*128*		*937*	**118**	**98**	**104**		**95**
	20221011_SdAR-M2_1		**99**		**104**		*280*	**96**	**95**												*151*		*125*	**103**	**99**	**90**	**91**
	20221011_SdAR-M2_2		**100**		**99**	*165*	*294*	**96**	**95**											**113**			*128*	**103**	**98**	**98**	**98**
	20221019_SdAR-M2_1	**84**	**110**		**105**	*160*	*296*	**99**	**98**								*127*	**120**		*140*			**119**	**103**	**106**	**98**	**103**
	20221019_SdAR-M2_2	**88**	**100**		**101**		*298*	**108**	**104**			*121*	*136*	*123*	*130*	*139*	*157*	*146*		*150*			*136*	**104**	**97**	**113**	**114**
	20221019_SdAR-M2_3		**99**		**105**		*307*	**111**	**108**			**119**	*139*	*123*	**117**		*146*	*135*		*143*	*162*		*128*	**106**	**93**	**93**	**98**
	20221206_WQB-1_1	*126*					*194*																*179*	**114**			**94**
	20221206_WQB-1_2	**94**					*186*																*178*	**109**			**93**
	20221206_WQB-1_3	**102**					*165*																*172*	**104**			77
	20221206_SdAR-M2_1	**99**	**98**		**106**	*172*	*359*	**97**	**96**			**109**	*140*	**117**	*122*	*126*	*141*	*139*		*152*	*155*		*128*	**103**	**107**	**105**	
	20221206_SdAR-M2_2	**99**	**97**		**106**		*270*	**105**	**103**			**115**	*137*	**114**		*122*	*124*	*131*		*131*	*136*		*128*	**103**	**108**	**101**	**113**
	20221206_SdAR-M2_3	**86**	**102**		**103**		*289*	**93**	**92**			**105**	*126*	**107**		**116**		*124*		*131*				**101**	**102**	**92**	
**Reference is total digestion**	20221213_SdAR-M2_1				**92**		33	**81**	**82**	78	**84**	79	51	73	68					61	52	*316*	**87**	**96**	**101**	75	59
	20221213_SdAR-M2_2										**92**	**85**			73	67	64				57						
	20221213_SdAR-M2_3				**94**		34	**81**	**82**	**81**	**87**			78	73	69				55		*273*	**88**	**99**	*121*	71	60
	20221220_SdAR-M2_1				**96**	66	30	**83**	**83**	**81**	**82**	75	49	66	62	58	54	54		53	48	*145*	**85**	**97**	**99**	73	52
	20221220_SdAR-M2_2				**92**	77	39	**82**	**82**	**80**	78	76	50	68	67	63	66	57	65	56	52	*155*	**84**	**96**	**93**	75	54
	20221220_SdAR-M2_3	**95**		61	**92**	69	35	**92**	**92**	**88**	**98**		53	**83**	78	70		62	62	56	54	*146*	**84**	**96**	**95**	**82**	52
	20220926_SdAR-M2_1				**86**		34	**83**	**84**	**84**	**94**								63	57		*299*	79	**97**	**102**		55
	20221011_SdAR-M2_1				**93**		31	**85**	**85**	**86**	**89**								55		58		**84**	**102**	**97**	71	53
	20221011_SdAR-M2_2				**88**	73	32	**84**	**85**	**87**									62	50			**86**	**102**	**96**	78	57
	20221019_SdAR-M2_1	**85**			**94**	70	33	**87**	**88**	**89**	**94**						65	61		62			80	**102**	**104**	78	60
	20221019_SdAR-M2_2	**88**			**90**		33	**95**	**94**	**92**	**97**	**98**	55	**89**	**82**	**83**	**80**	74	65	67			**91**	**103**	**95**	**90**	66
	20221019_SdAR-M2_3				**94**		34	**98**	**97**	**99**	**102**	**96**	56	**90**	74		75	69		64	62		**86**	**105**	**91**	74	57
	20221206_SdAR-M2_1	**100**			**94**	75	40	**86**	**86**	**83**	**91**	**88**	56	**85**	77	75	72	71		68	60		**85**	**102**	**105**	**84**	
	20221206_SdAR-M2_2	**99**			**95**		30	**93**	**93**	**92**	**97**	**93**	55	**83**		73	64	67		58	52		**86**	**102**	**106**	**81**	65
	20221206_SdAR-M2_3	**86**			**92**		32	**82**	**83**	**81**	**87**	**85**	51	78		69		63	57	58				**100**	**100**	73	

Table S 15: LOQs and blank levels of the digestion blanks, if detected > LOQ, in µg/l during the measurement of the case study samples.

	Li	Be	B	Na	Mg	Al	Si	P	S	K	Ca	Sc	Ti	V	Cr	Mn	Fe
LOQ	*0.21*	*0.00162*	*2.11*	*69.4*	*1.5*	*0.503*	*5.33*	*0.383*	*23.4*	*112*	*23.2*	*0.0125*	*0.0219*	*0.0129*	*0.196*	*0.0541*	*0.669*
LOQ	*0.0238*	*0.00402*	*0.805*	*6.09*	*0.482*	*NA*	*2.02*	*0.175*	*NA*	*65*	*11.4*	*0.0092*	*0.0796*	*0.000684*	*0.0971*	*0.0222*	*0.134*
LOQ	*0.0709*	*0.0042*	*2.08*	*30.4*	*1.49*	*0.37*	*4.45*	*0.788*	*4.03*	*211*	*26*	*0.0219*	*0.139*	*0.0217*	*0.163*	*0.39*	*0.682*
20221213_Blank1	<LOQ	<LOQ	<LOQ	<LOQ	RSD>10%	12.04525	RSD>10%	RSD>10%	RSD>10%	<LOQ	761.7733	<LOQ	<LOQ	RSD>10%	<LOQ	<LOQ	49.84793
20221213_Blank2	<LOQ	<LOQ	<LOQ	<LOQ	RSD>10%	61.00436	RSD>10%	<LOQ	RSD>10%	<LOQ	1076.984	<LOQ	2.999094	RSD>10%	<LOQ	<LOQ	3.500238
20221213_Blank3	RSD>10%	<LOQ	<LOQ	RSD>10%	RSD>10%	RSD>10%	RSD>10%	<LOQ	RSD>10%	RSD>10%	353.3594	<LOQ	<LOQ	RSD>10%	<LOQ	<LOQ	10.27518
20221220_Blank1	<LOQ	<LOQ	<LOQ	RSD>10%	RSD>10%	RSD>10%	60.15351	<LOQ	RSD>10%	<LOQ	RSD>10%	<LOQ	<LOQ	RSD>10%	<LOQ	<LOQ	<LOQ
20221220_Blank2	<LOQ	<LOQ	<LOQ	RSD>10%	RSD>10%	RSD>10%	RSD>10%	<LOQ	RSD>10%	<LOQ	RSD>10%	<LOQ	<LOQ	RSD>10%	<LOQ	<LOQ	<LOQ
20221220_Blank3	<LOQ	<LOQ	<LOQ	<LOQ	RSD>10%	7.318797	<LOQ	<LOQ	RSD>10%	<LOQ	812.8846	<LOQ	<LOQ	RSD>10%	<LOQ	<LOQ	<LOQ
20221206_Blank2	RSD>10%	<LOQ	<LOQ	<LOQ	<LOQ	<LOQ	<LOQ	<LOQ	<LOQ	<LOQ	<LOQ	<LOQ	<LOQ	<LOQ	<LOQ	<LOQ	<LOQ
20221206_Blank3	<LOQ	NA	<LOQ	<LOQ	<LOQ	RSD>10%	<LOQ	<LOQ	<LOQ	<LOQ	<LOQ	<LOQ	<LOQ	<LOQ	<LOQ	<LOQ	<LOQ
20220926_Blank_1	<LOQ	NA	<LOQ	<LOQ	RSD>10%	<LOQ	395.0432	<LOQ	<LOQ	<LOQ	RSD>10%	<LOQ	<LOQ	<LOQ	<LOQ	<LOQ	45.33192
20220926_Blank_2	RSD>10%	NA	<LOQ	<LOQ	39.60098	<LOQ	265.347	<LOQ	<LOQ	<LOQ	RSD>10%	<LOQ	<LOQ	<LOQ	<LOQ	<LOQ	<LOQ
20220926_Blank_3	<LOQ	<LOQ	<LOQ	<LOQ	RSD>10%	<LOQ	249.1039	<LOQ	<LOQ	<LOQ	<LOQ	<LOQ	<LOQ	<LOQ	<LOQ	<LOQ	<LOQ
20221011_Blank_1	RSD>10%	<LOQ	<LOQ	<LOQ	<LOQ	<LOQ	<LOQ	<LOQ	<LOQ	<LOQ	<LOQ	<LOQ	<LOQ	<LOQ	<LOQ	<LOQ	<LOQ
20221011_Blank_3	RSD>10%	NA	<LOQ	<LOQ	<LOQ	<LOQ	162.9678	<LOQ	<LOQ	<LOQ	RSD>10%	<LOQ	<LOQ	<LOQ	<LOQ	<LOQ	136.4017

	Co	Ni	Cu	Zn	Ga	Ge	As	Se	Br	Rb	Sr	Y	Zr	Nb	Mo	Ru
LOQ	*0.00154*	*1.41*	*0.713*	*NA*	*0.000648*	*0.00437*	*0.0146*	*0.0517*	*16.9*	*0.00948*	*0.37*	*0.000325*	*2.09*	*0.0324*	*0.00358*	*0.00555*
LOQ	*0.00229*	*NA*	*NA*	*NA*	*0.00118*	*0.00965*	*0.00715*	*0.0247*	*17.3*	*0.00992*	*0.0432*	*0.000299*	*0.3*	*0.0143*	*0.00289*	*0.00117*
LOQ	*0.00603*	*0.0028*	*0.00843*	*0.218*	*0.00319*	*0.0135*	*0.032*	*0.198*	*46*	*0.0317*	*0.0498*	*0.00281*	*1.85*	*0.115*	*0.00475*	*0.00362*
20221213_Blank1	<LOQ	RSD>10%	RSD>10%	RSD>10%	RSD>10%	<LOQ	<LOQ	<LOQ	507.7237	<LOQ	<LOQ	0.007903	<LOQ	<LOQ	<LOQ	<LOQ
20221213_Blank2	<LOQ	RSD>10%	RSD>10%	6.898328	RSD>10%	<LOQ	<LOQ	<LOQ	RSD>10%	<LOQ	RSD>10%	RSD>10%	<LOQ	RSD>10%	<LOQ	<LOQ
20221213_Blank3	<LOQ	RSD>10%	RSD>10%	RSD>10%	RSD>10%	<LOQ	<LOQ	<LOQ	RSD>10%	<LOQ	<LOQ	<LOQ	<LOQ	<LOQ	<LOQ	<LOQ
20221220_Blank1	<LOQ	RSD>10%	RSD>10%	RSD>10%	RSD>10%	<LOQ	<LOQ	<LOQ	RSD>10%	<LOQ	<LOQ	<LOQ	<LOQ	<LOQ	<LOQ	RSD>10%
20221220_Blank2	<LOQ	RSD>10%	RSD>10%	14.74515	RSD>10%	<LOQ	<LOQ	<LOQ	RSD>10%	<LOQ	<LOQ	<LOQ	<LOQ	<LOQ	<LOQ	<LOQ
20221220_Blank3	<LOQ	RSD>10%	RSD>10%	RSD>10%	0.03904	<LOQ	<LOQ	<LOQ	<LOQ	<LOQ	<LOQ	<LOQ	<LOQ	RSD>10%	<LOQ	<LOQ
20221206_Blank2	<LOQ	<LOQ	RSD>10%	9.120799	0.031193	RSD>10%	<LOQ	RSD>10%	508.2933	<LOQ	<LOQ	<LOQ	<LOQ	<LOQ	<LOQ	<LOQ
20221206_Blank3	<LOQ	<LOQ	RSD>10%	13.40241	RSD>10%	RSD>10%	<LOQ	<LOQ	RSD>10%	<LOQ	<LOQ	<LOQ	<LOQ	<LOQ	<LOQ	<LOQ

Table S 15 continued.

20220926_Blank_1	<LOQ	RSD>10%	RSD>10%	RSD>10%	RSD>10%	RSD>10%	<LOQ	<LOQ	<LOQ	<LOQ	<LOQ	<LOQ	<LOQ	RSD>10%	<LOQ	NA	
20220926_Blank_2	RSD>10%	RSD>10%	0.501936	<LOQ	<LOQ	<LOQ	<LOQ	<LOQ	<LOQ	<LOQ	<LOQ	<LOQ	<LOQ	RSD>10%	RSD>10%	<LOQ	
20220926_Blank_3	<LOQ	RSD>10%	0.347021	<LOQ	<LOQ	<LOQ	<LOQ	<LOQ	<LOQ	<LOQ	<LOQ	<LOQ	<LOQ	RSD>10%	<LOQ	<LOQ	
20221011_Blank_1	<LOQ	RSD>10%	<LOQ	<LOQ	<LOQ	<LOQ	<LOQ	<LOQ	1248.125	<LOQ	<LOQ	<LOQ	<LOQ	RSD>10%	<LOQ	NA	
20221011_Blank_3	<LOQ	RSD>10%	0.278558	6.573998	<LOQ	<LOQ	<LOQ	<LOQ	<LOQ	<LOQ	<LOQ	<LOQ	<LOQ	RSD>10%	<LOQ	<LOQ	

	**Ag**	**Cd**	**In**	**Sn**	**Sb**	**Te**	**Cs**	**Ba**	**La**	**Ce**	**Pr**	**Nd**	**Sm**	**Eu**	**Gd**	**Tb**	**Dy**
LOQ	*0.000862*	*0.000735*	*0.000248*	*0.0468*	*0.0269*	*0.1*	*0.0017*	*0.0301*	*0.000507*	*0.00064*	*7.76E-05*	*0.000377*	*0.000546*	*0.000114*	*0.00169*	*6.85E-05*	*0.0002*
LOQ	*0.000897*	*0.00322*	*0.000227*	*6.67E-05*	*0.00382*	*0.0355*	*0.00194*	*0.0142*	*0.000325*	*0.000236*	*4.3E-06*	*0.000322*	*0.000328*	*0.000246*	*0.00151*	*8.48E-05*	*0.00025*
LOQ	*0.000596*	*0.00129*	*0.000496*	*0.0376*	*0.0217*	*0.0475*	*0.002*	*0.0224*	*0.00042*	*0.000685*	*0.00019*	*0.00062*	*0.00175*	*0.000314*	*0.00502*	*0.000194*	*0.00084*
20221213_Blank1	<LOQ	<LOQ	<LOQ	RSD>10%	<LOQ	<LOQ	<LOQ	<LOQ	RSD>10%	RSD>10%	RSD>10%	RSD>10%	<LOQ	<LOQ	<LOQ	<LOQ	<LOQ
20221213_Blank2	<LOQ	<LOQ	<LOQ	RSD>10%	<LOQ	<LOQ	<LOQ	<LOQ	<LOQ	RSD>10%	RSD>10%	RSD>10%	<LOQ	NA	<LOQ	<LOQ	<LOQ
20221213_Blank3	<LOQ	<LOQ	<LOQ	RSD>10%	<LOQ	<LOQ	<LOQ	<LOQ	<LOQ	RSD>10%	RSD>10%	<LOQ	RSD>10%	<LOQ	RSD>10%	<LOQ	<LOQ
20221220_Blank1	<LOQ	<LOQ	<LOQ	RSD>10%	RSD>10%	<LOQ	<LOQ	<LOQ	<LOQ	<LOQ	RSD>10%	<LOQ	<LOQ	NA	<LOQ	<LOQ	<LOQ
20221220_Blank2	RSD>10%	<LOQ	RSD>10%	RSD>10%	RSD>10%	<LOQ	<LOQ	<LOQ	<LOQ	<LOQ	RSD>10%	<LOQ	<LOQ	<LOQ	<LOQ	<LOQ	<LOQ
20221220_Blank3	<LOQ	<LOQ	<LOQ	RSD>10%	RSD>10%	<LOQ	<LOQ	<LOQ	RSD>10%	<LOQ	RSD>10%	<LOQ	<LOQ	<LOQ	<LOQ	<LOQ	<LOQ
20221206_Blank2	RSD>10%	RSD>10%	<LOQ	<LOQ	RSD>10%	RSD>10%	<LOQ	<LOQ	<LOQ	<LOQ	<LOQ	<LOQ	RSD>10%	<LOQ	<LOQ	<LOQ	<LOQ
20221206_Blank3	<LOQ	<LOQ	<LOQ	<LOQ	RSD>10%	RSD>10%	<LOQ	RSD>10%	RSD>10%	<LOQ	RSD>10%	RSD>10%	<LOQ	<LOQ	<LOQ	<LOQ	<LOQ
20220926_Blank_1	<LOQ	NA	<LOQ	<LOQ	<LOQ	<LOQ	<LOQ	<LOQ	<LOQ	<LOQ	<LOQ	<LOQ	<LOQ	<LOQ	<LOQ	RSD>10%	<LOQ
20220926_Blank_2	RSD>10%	NA	<LOQ	<LOQ	RSD>10%	<LOQ	<LOQ	<LOQ	<LOQ	<LOQ	<LOQ	<LOQ	<LOQ	<LOQ	RSD>10%	RSD>10%	RSD>10%
20220926_Blank_3	RSD>10%	NA	<LOQ	<LOQ	<LOQ	<LOQ	<LOQ	<LOQ	<LOQ	<LOQ	<LOQ	<LOQ	<LOQ	<LOQ	<LOQ	RSD>10%	RSD>10%
20221011_Blank_1	<LOQ	<LOQ	RSD>10%	<LOQ	RSD>10%	<LOQ	<LOQ	<LOQ	<LOQ	0.026197	<LOQ	<LOQ	<LOQ	<LOQ	<LOQ	<LOQ	<LOQ
20221011_Blank_3	<LOQ	NA	<LOQ	<LOQ	<LOQ	<LOQ	<LOQ	<LOQ	<LOQ	<LOQ	<LOQ	<LOQ	<LOQ	<LOQ	<LOQ	<LOQ	<LOQ

	**Ho**	**Er**	**Tm**	**Yb**	**Lu**	**Hf**	**Ta**	**W**	**Ir**	**Pt**	**Au**	**Hg**	**Tl**	**Pb**	**Bi**	**Th**	**U**
LOQ	*8.32E-05*	*0.000221*	*3.64E-05*	*0.000223*	*0.000144*	*0.101*	*0.0173*	*0.111*	*0.0062*	*0.00105*	*0.0128*	*0.0989*	*0.000214*	*0.0199*	*0.0017*	*0.00401*	*0.00054*
LOQ	*7.18E-05*	*0.000155*	*4.63E-05*	*0.000181*	*9.13E-05*	*0.0168*	*0.0181*	*0.03*	*0.000433*	*2.89E-05*	*0.00222*	*0.0259*	*0.00139*	*0.0109*	*NA*	*0.0028*	*0.00032*
LOQ	*0.00031*	*0.000518*	*9.09E-05*	*0.000176*	*0.00036*	*0.0962*	*0.0558*	*0.104*	*0.0087*	*0.00137*	*0.0138*	*0.0985*	*0.000995*	*0.00444*	*NA*	*0.00428*	*0.00036*
20221213_Blank1	<LOQ	<LOQ	NA	<LOQ	<LOQ	<LOQ	0.90947	3.904559	RSD>10%	RSD>10%	<LOQ	<LOQ	<LOQ	<LOQ	0.014145	<LOQ	<LOQ
20221213_Blank2	<LOQ	<LOQ	<LOQ	<LOQ	RSD>10%	<LOQ	RSD>10%	3.199884	<LOQ	RSD>10%	<LOQ	<LOQ	<LOQ	<LOQ	RSD>10%	<LOQ	<LOQ
20221213_Blank3	RSD>10%	<LOQ	<LOQ	<LOQ	<LOQ	<LOQ	RSD>10%	RSD>10%	<LOQ	RSD>10%	<LOQ	<LOQ	<LOQ	<LOQ	RSD>10%	<LOQ	<LOQ
20221220_Blank1	RSD>10%	RSD>10%	<LOQ	<LOQ	<LOQ	<LOQ	RSD>10%	RSD>10%	<LOQ	RSD>10%	RSD>10%	<LOQ	RSD>10%	<LOQ	RSD>10%	<LOQ	<LOQ

Table S 15 continued.

20221220_Blank2	RSD>10%	RSD>10%	NA	<LOQ	RSD>10%	<LOQ	RSD>10%	<LOQ	<LOQ	RSD>10%	RSD>10%	<LOQ	<LOQ	<LOQ	RSD>10%	<LOQ	<LOQ
20221220_Blank3	RSD>10%	RSD>10%	NA	<LOQ	RSD>10%	<LOQ	RSD>10%	RSD>10%	<LOQ	<LOQ	RSD>10%	<LOQ	<LOQ	<LOQ	RSD>10%	<LOQ	<LOQ
20221206_Blank2	<LOQ	<LOQ	0.010314	<LOQ	<LOQ	<LOQ	RSD>10%	2.89922	<LOQ	<LOQ	<LOQ	<LOQ	<LOQ	<LOQ	<LOQ	<LOQ	<LOQ
20221206_Blank3	<LOQ	<LOQ	<LOQ	<LOQ	<LOQ	<LOQ	RSD>10%	<LOQ	<LOQ	<LOQ	<LOQ	<LOQ	RSD>10%	<LOQ	<LOQ	<LOQ	RSD>10%
20220926_Blank_1	<LOQ	<LOQ	<LOQ	<LOQ	<LOQ	<LOQ	<LOQ	3.008513	<LOQ	<LOQ	<LOQ	<LOQ	<LOQ	<LOQ	RSD>10%	<LOQ	<LOQ
20220926_Blank_2	<LOQ	RSD>10%	<LOQ	NA	<LOQ	<LOQ	RSD>10%	2.70668	<LOQ	<LOQ	<LOQ	<LOQ	<LOQ	<LOQ	RSD>10%	<LOQ	<LOQ
20220926_Blank_3	<LOQ	<LOQ	<LOQ	<LOQ	RSD>10%	<LOQ	<LOQ	<LOQ	<LOQ	<LOQ	<LOQ	<LOQ	<LOQ	<LOQ	RSD>10%	<LOQ	<LOQ
20221011_Blank_1	<LOQ	RSD>10%	NA	NA	RSD>10%	<LOQ	<LOQ	<LOQ	<LOQ	<LOQ	<LOQ	RSD>10%	NA	<LOQ	RSD>10%	<LOQ	<LOQ
20221011_Blank_3	<LOQ	<LOQ	<LOQ	RSD>10%	<LOQ	<LOQ	<LOQ	<LOQ	<LOQ	<LOQ	<LOQ	RSD>10%	<LOQ	<LOQ	RSD>10%	<LOQ	<LOQ

9.2 Rhine in Koblenz

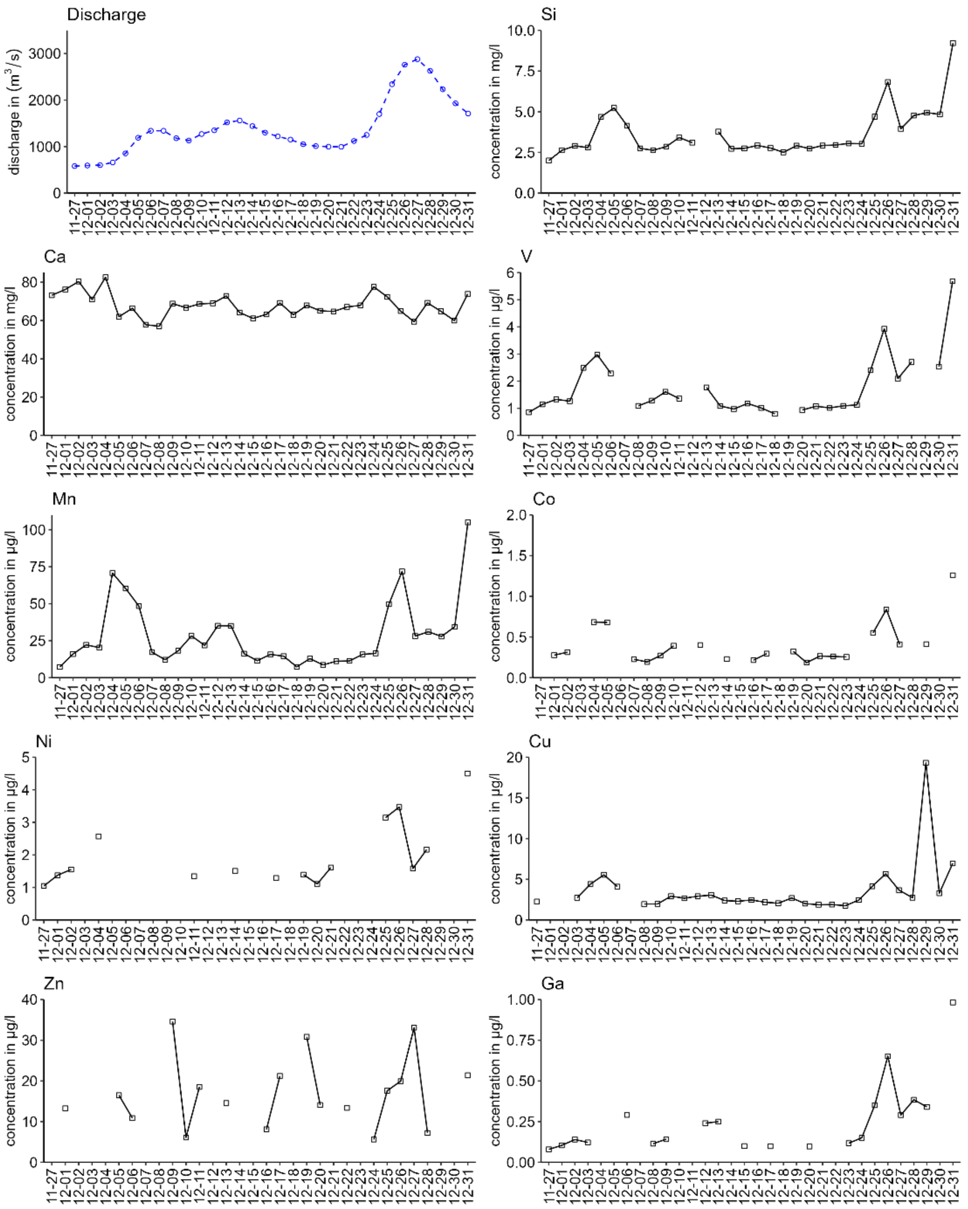

Figure S 6: Concentrations in µg/l in whole water samples digested with the optimised method in comparison to the discharge in m³/s from 27th November to 31st December 2018 at the River Rhine in Koblenz. All concentrations < LOQ and with RSD >10 % are not depicted. Only elements with data points n > 15 are depicted.

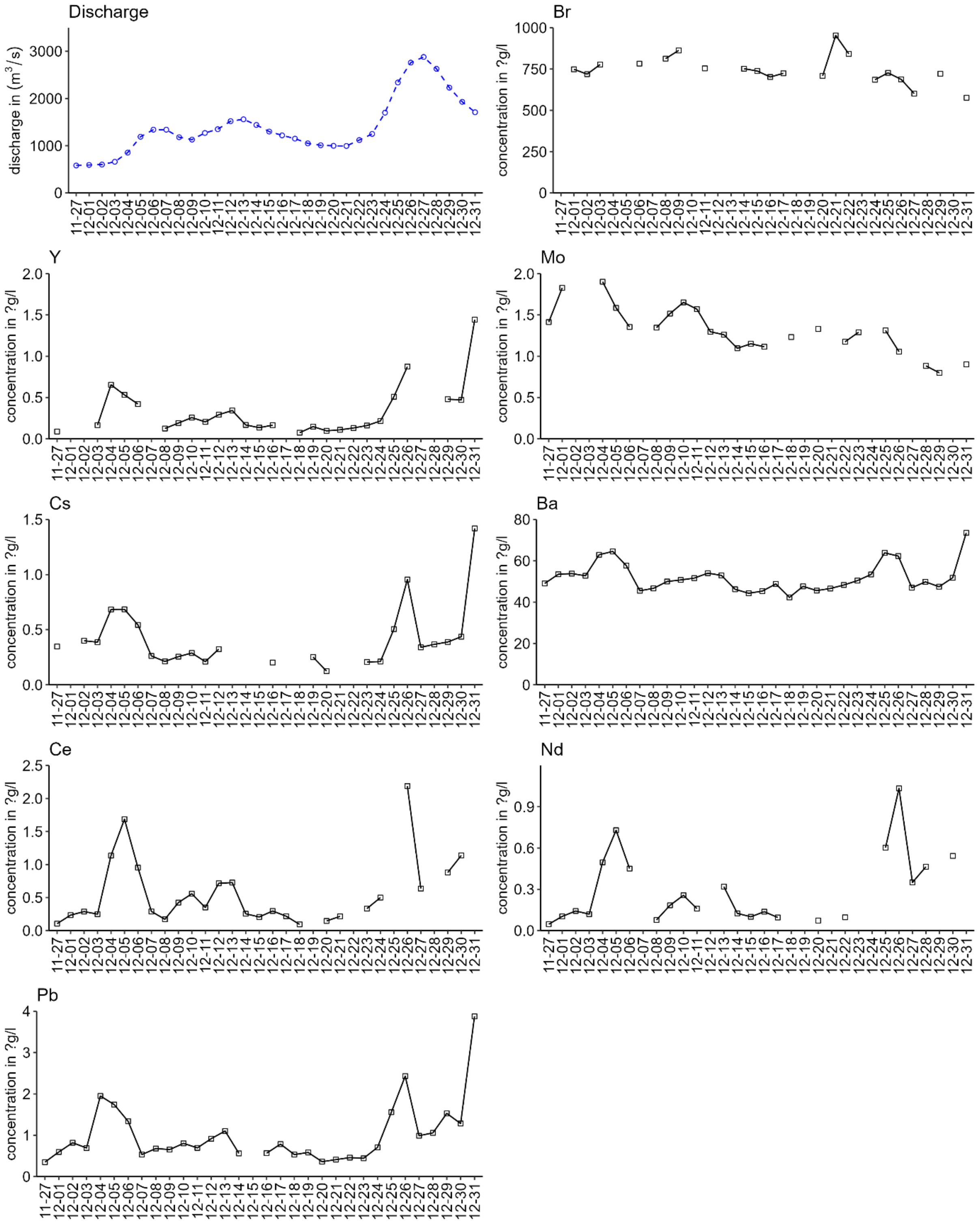

Figure S 7: Concentrations in µg/l in whole water samples digested with the optimised method in comparison to the discharge in m³/s from 27th November to 31st December 2018 at the River Rhine in Koblenz. All concentrations < LOQ and with RSD >10 % are not depicted. Only elements with data points n > 15 are depicted.

9.3 Moselle in Koblenz

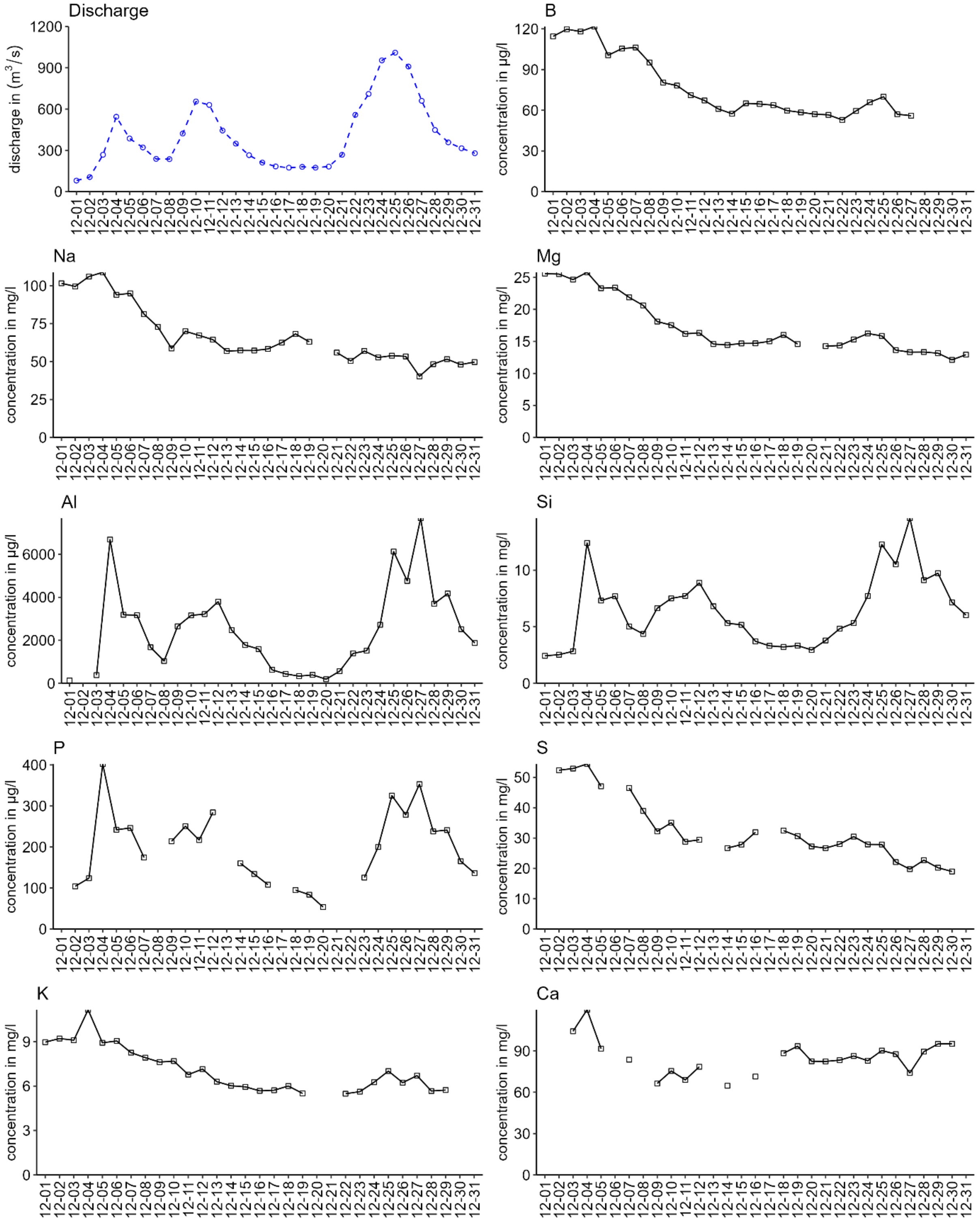

Figure S 8: Concentrations in mg/l and µg/l in whole water samples digested with the optimised method in comparison to the discharge in m³/s from 1st December to 31st December 2018 at the River Moselle in Koblenz. All concentrations < LOQ and with RSD >10 % are not depicted. Only elements with data points n > 15 are depicted.

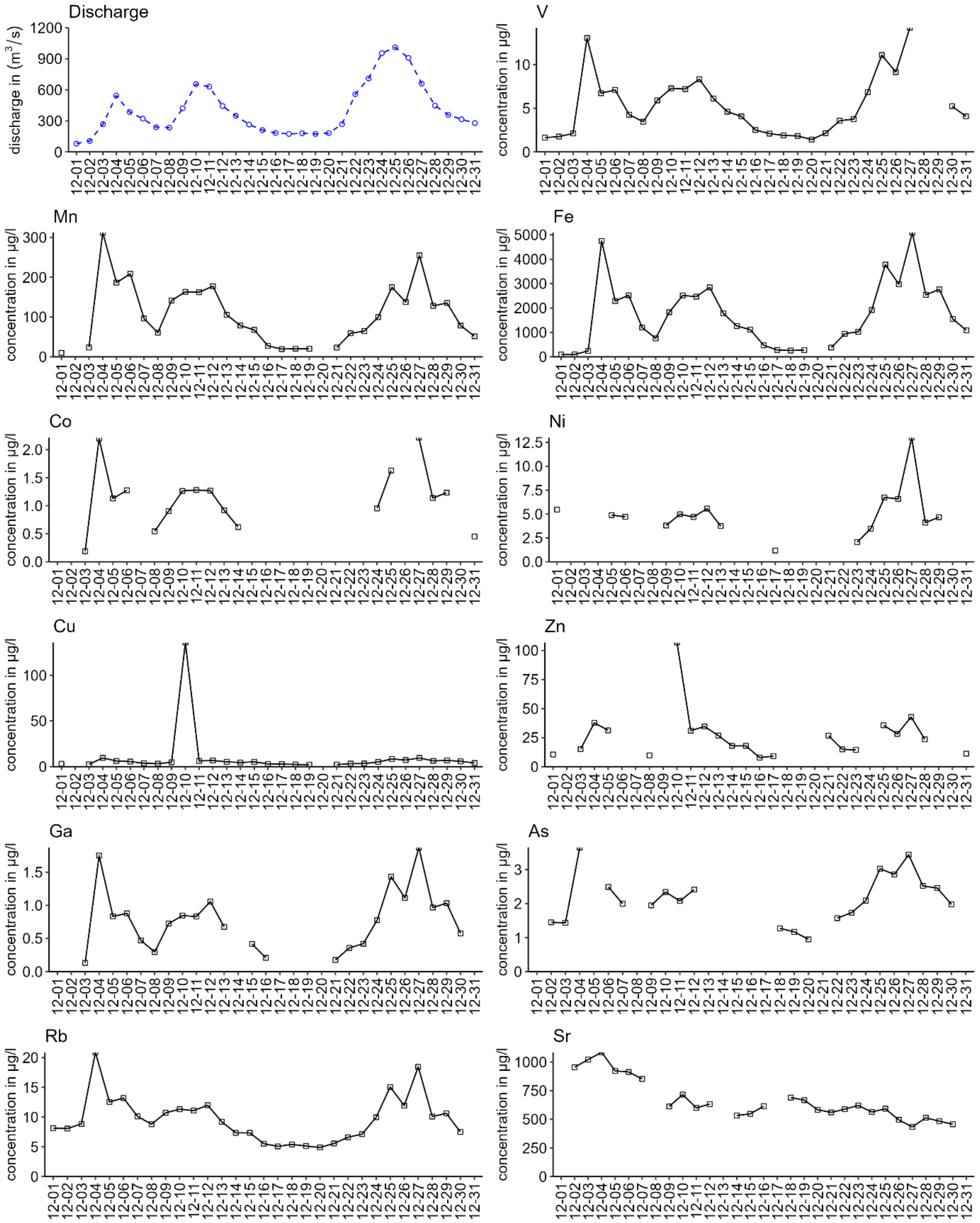

Figure S 9: Concentrations in µg/l in whole water samples digested with the optimised method in comparison to the discharge in m³/s from 1st December to 31st December 2018 at the River Moselle in Koblenz. All concentrations < LOQ and with RSD >10 % are not depicted. Only elements with data points n > 15 are depicted.

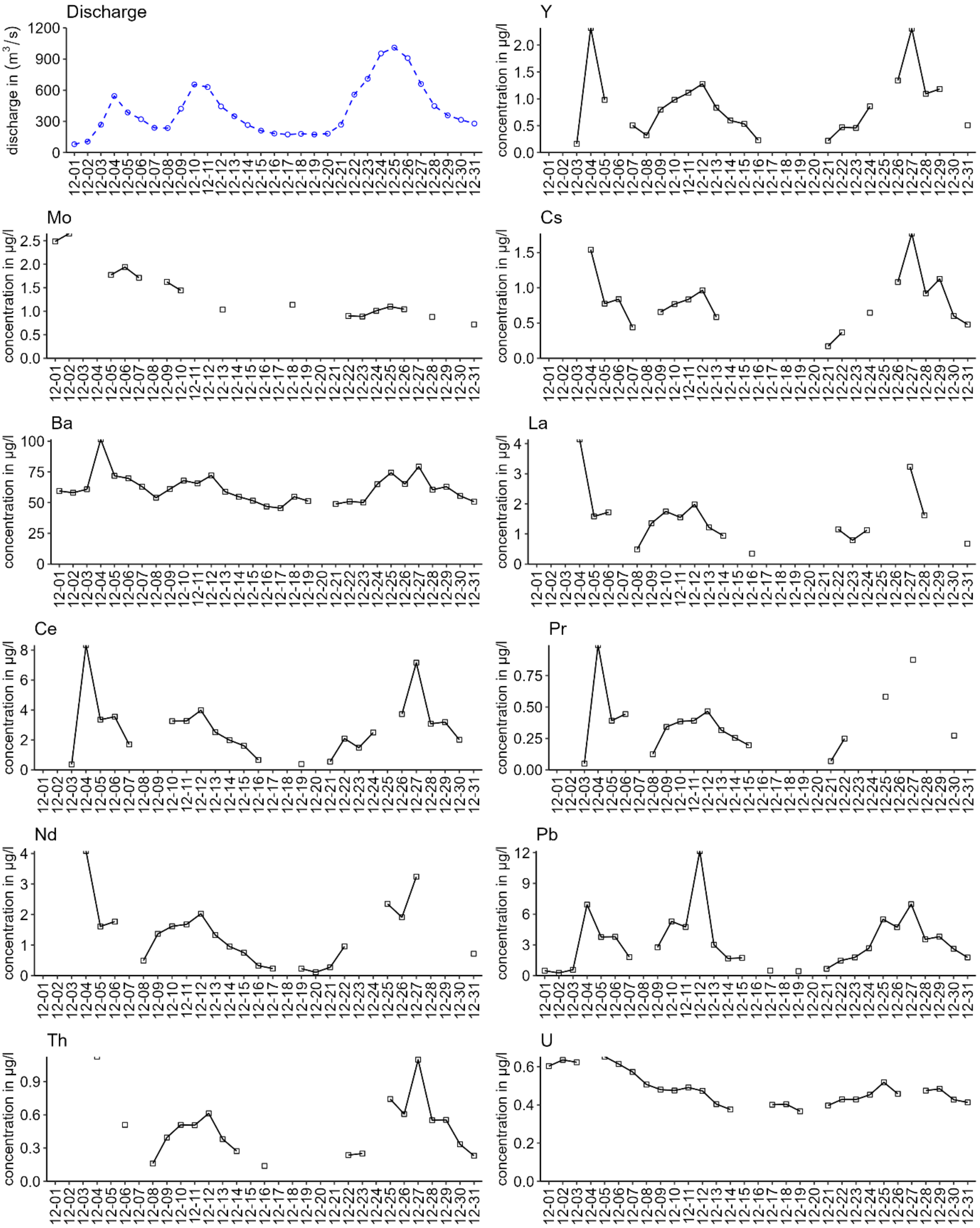

Figure S 10: Concentrations in µg/l in whole water samples digested with the optimised method in comparison to the discharge in m^3/s from 1st December to 31st December 2018 at the River Moselle in Koblenz. All concentrations < LOQ and with RSD >10 % are not depicted. Only elements with data points n > 15 are depicted.

9.4 Rhine in Wesel

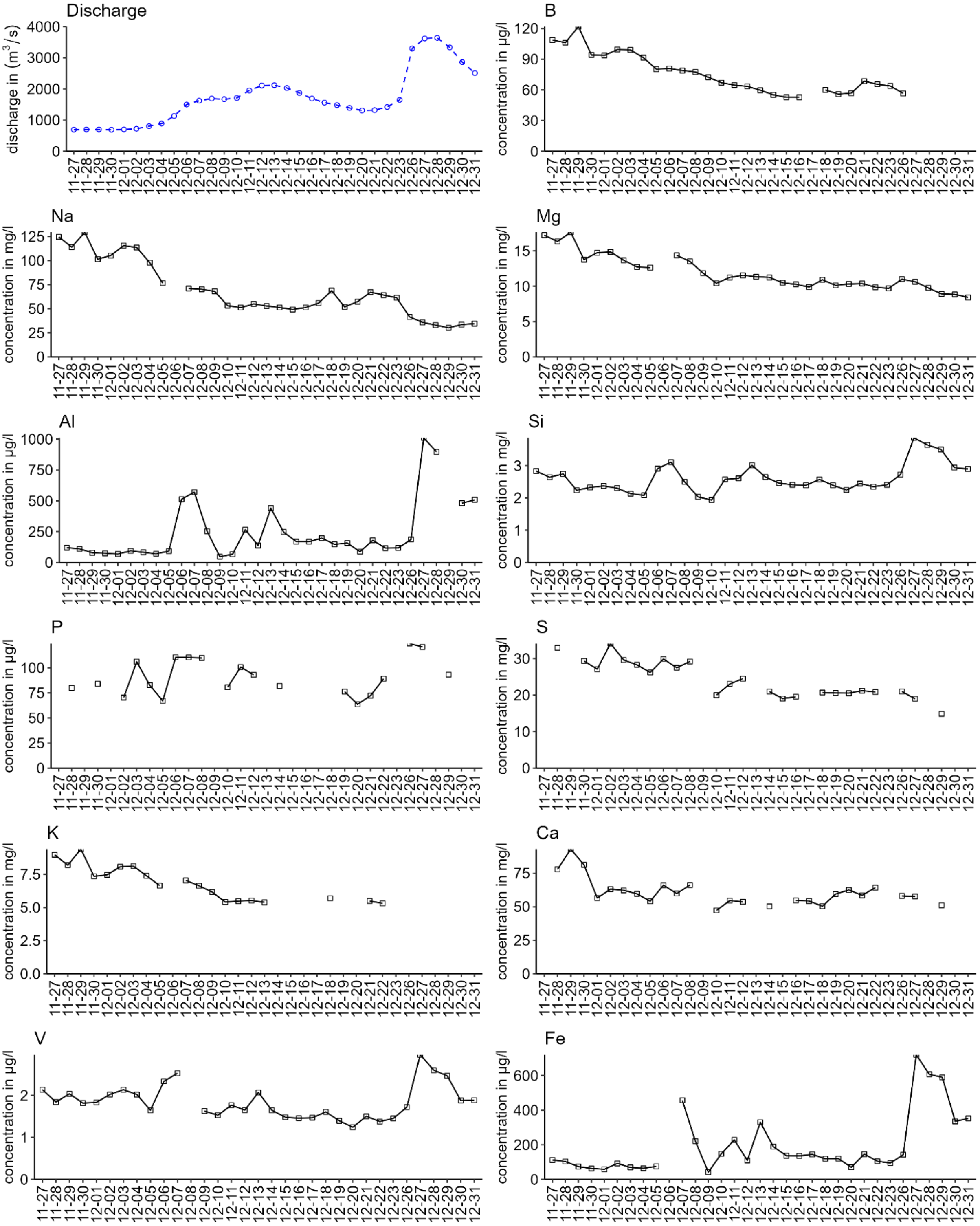

Figure S 11: Concentrations in mg/l and µg/l in whole water samples digested with the optimised method in comparison to the discharge in m³/s from 27th November to 31st December 2018 at the River Rhine in Wesel. All concentrations < LOQ and with RSD >10 % are not depicted. Only elements with data points n > 15 are depicted.

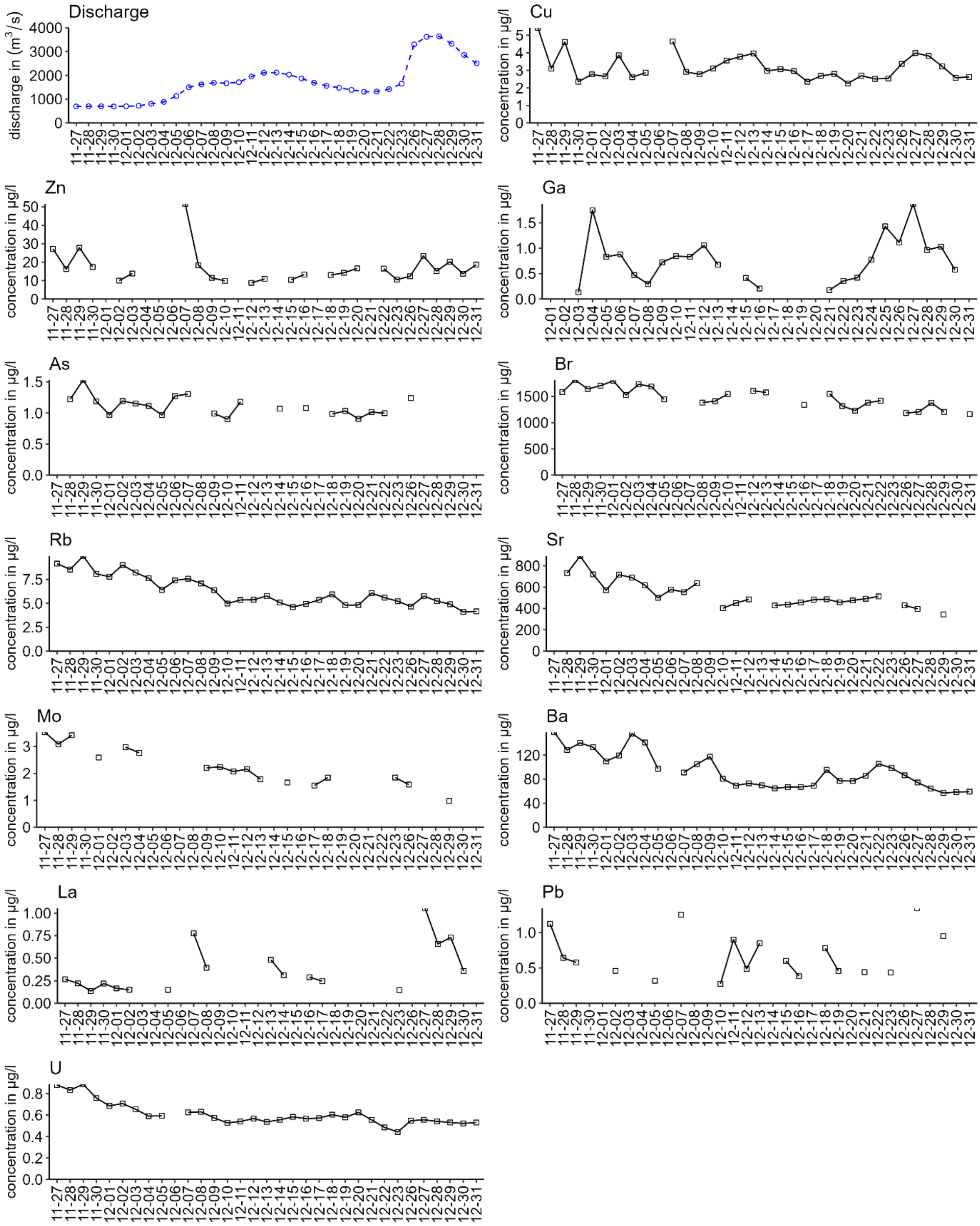

Figure S 12: Concentrations in µg/l in whole water samples digested with the optimised method in comparison to the discharge in m^3/s from 27th November to 31st December 2018 at the River Rhine in Wesel. All concentrations < LOQ and with RSD >10 % are not depicted. Only elements with data points n > 15 are depicted.

9.5 Rhine in Weil

Unlike the other sampling stations, Rhine/Weil does not show a highly varying discharge. However, peaks are observed for Al, Si, P, V, Mn, Ga, As, Rb, Y, Cs, Nd and Pb between 24th December and 29th December. This indicates that a higher particle load could be the reason, e.g. caused by a regional rain event which did not contribute significantly to the discharge, but which caused a significant wash-off effect. Elements which rather show a dilution effect at the other sampling stations such as Na, Mg or U due to their dissolved nature do not show the same peak.

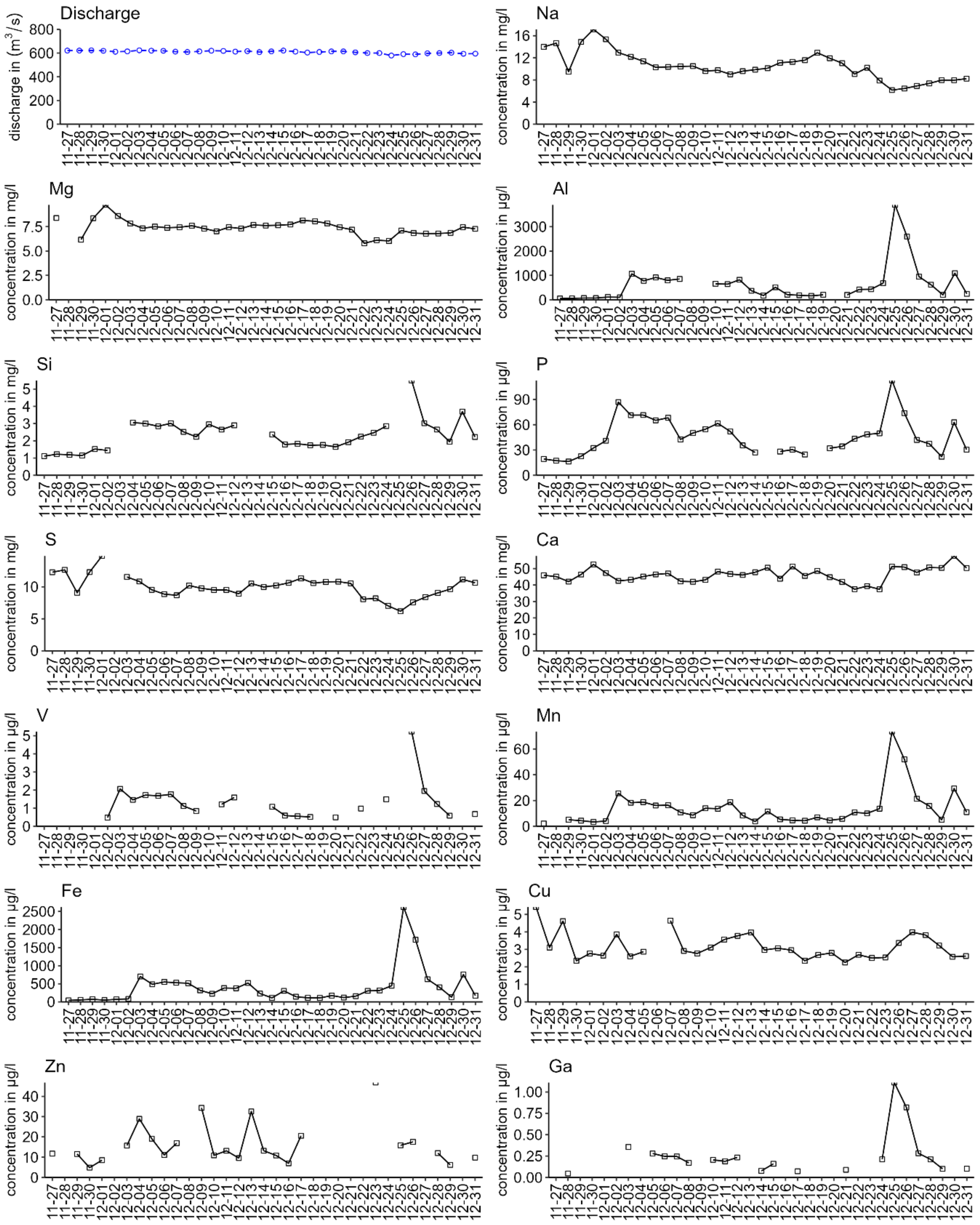

Figure S 13: Concentrations in mg/l and µg/l in whole water samples digested with the optimised method in comparison to the discharge in m³/s from 27th November to 31st December 2018 at the

River Rhine in Weil. All concentrations < LOQ and with RSD >10 % are not depicted. Only elements with data points n > 15 are depicted.

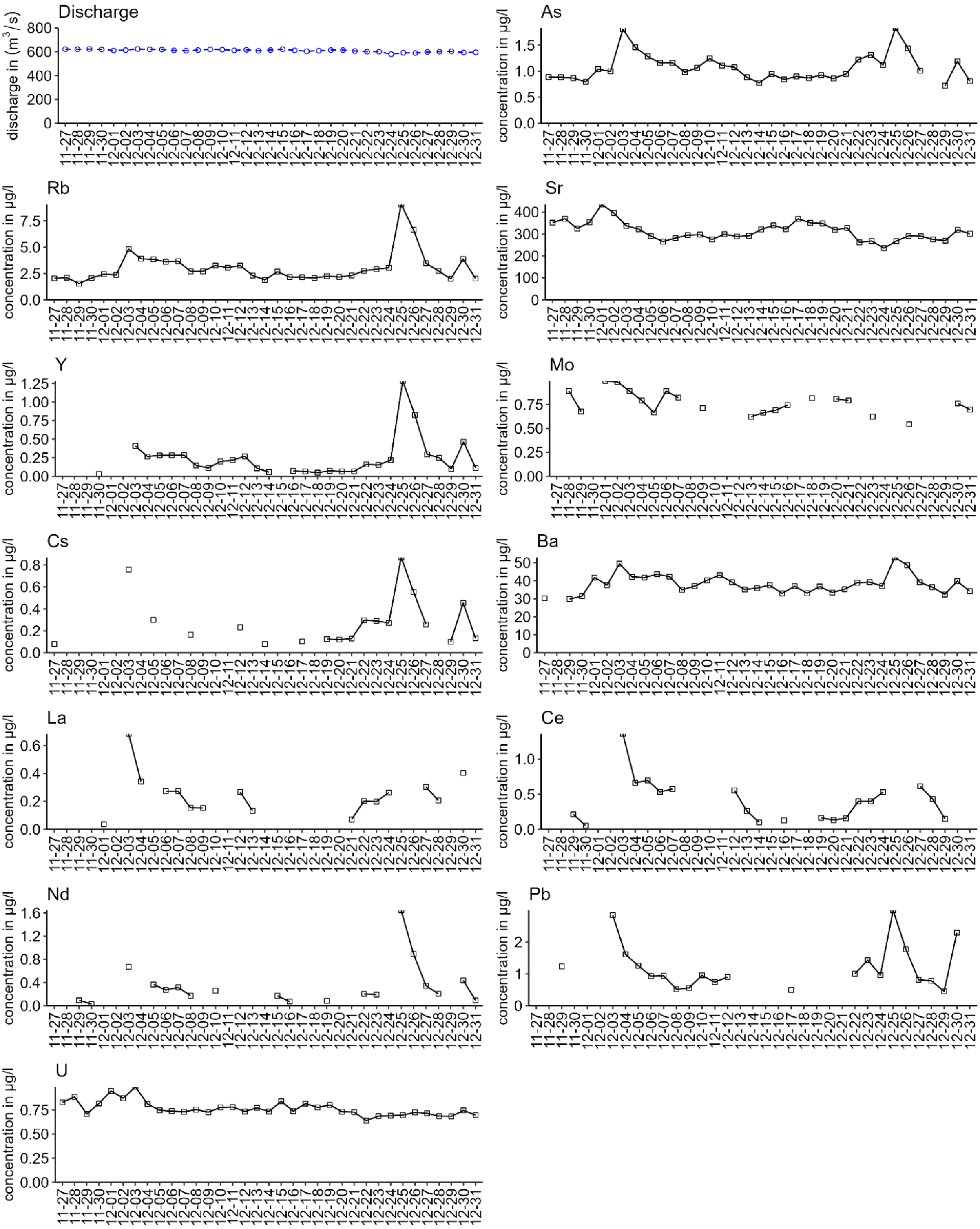

Figure S 14: Concentrations in µg/l in whole water samples digested with the optimised method in comparison to the discharge in m^3/s from 27th November to 31st December 2018 at the River Rhine in Weil. All concentrations < LOQ and with RSD >10 % are not depicted. Only elements with data points n > 15 are depicted.

10. References

Belkouteb, N., Schroeder, H., Arndt, J., Wiederhold, J.G., Ternes, T.A., Duester, L., 2023. Quantification of 68 elements in river water monitoring samples in single-run measurements. Chemosphere 320, 138053.

Sastre, J., Sahuquillo, A., Vidal, M., Rauret, G., 2002. Determination of Cd, Cu, Pb and Zn in environmental samples: microwave-assisted total digestion versus aqua regia and nitric acid extraction. Analytica Chimica Acta 462, 59-72.
